# Synergistic Anti-Tumor Efficacy Achieved by Reversing Drug Resistance through the Regulation of the Tumor Immune Microenvironment with IL-12 and Osimertinib Combination Therapy

**DOI:** 10.7150/jca.95407

**Published:** 2024-06-17

**Authors:** Huiqin Ding, Lijuan Wu, Huan Qin, Wenhui Fu, Yajun Wang, Mingyuan Wu, Jiangang Wang, Yantao Han

**Affiliations:** 1 School of Basic Medical Sciences, Qingdao University, Qingdao 266021, China.; 2 Marine Biomedical Research Institute of Qingdao, Ocean University of China Qingdao 266071, China.; 3 School of Pharmacy, Ocean University of China, Qingdao 266003, China.

**Keywords:** Interleukin 12 (IL-12), Osimertinib, tumor immune microenvironment (TIM), combination therapy, Reversal of Drug Resistance

## Abstract

The objective of this study was to investigate the role of IL-12 in enhancing the anti-tumor efficacy of the small molecule targeted drug osimertinib in resistant tumor models and reversing resistance mechanisms. We utilized paired non-small cell lung cancer H1975 tumor tissues, establishing mouse tumor models with diverse tumor immune microenvironments. Analytical methods including immunohistochemistry and immunofluorescence were employed to compare immune cell infiltration, cytokines, effector molecules, and protein changes in resistant signaling pathways in tumor tissues, shedding light on IL-12's mechanism of action in enhancing osimertinib efficacy and reversing resistance. Results showed that osimertinib monotherapy had limited tumor suppression, whereas IL-12 exhibited more significant anti-tumor effects. Combination therapy groups demonstrated even greater tumor suppression with increased immune cell infiltration, elevated immune-related factor secretion, reduced immunosuppressive MDSCs, and decreased resistance-related signaling pathway markers. In conclusion, IL-12 enhances anti-tumor efficacy and reverses osimertinib resistance through various mechanisms, including increased immune cell infiltration, reduced immunosuppressive MDSCs, enhanced immune cell granzyme and IFN-γ release, decreased PDL-1 expression, improved tumor microenvironment, restored immune surveillance, and heightened cancer cell sensitivity to osimertinib.

## Introduction

Osimertinib, a third-generation EGFR-TKI, is approved for treating NSCLC patients who progressed after first-line EGFR-TKI therapy with the development of the EGFR-T790M resistance mutation. However, resistance to osimertinib inevitably occurs, with the C797S mutation being the most common after 10-14 months of treatment 1. Currently, there is no targeted therapy specifically addressing the C797S mutation 2, and global efforts are underway to develop fourth-generation targeted therapies to overcome resistance. However, the iterative pace of EGFR-TKI development lags behind the emergence of resistance, necessitating exploration of alternative treatment approaches.

Given the significant aggregation of innate immune cells in the tumor microenvironment and their crucial roles in processes such as cytokine release, cell recruitment, and activation of acquired immune cells, targeting innate immune cells has become a key focus in the next generation of immunotherapy research. IL-12, as a multifunctional cytokine bridging specific and nonspecific immunity, has been shown to exert anti-tumor effects by modulating innate and acquired immunity and improving the tumor microenvironment.

In light of these considerations, we explore the potential benefits of combining IL-12 with osimertinib in the treatment of non-small cell lung cancer (NSCLC). Competitive resistance mechanisms to the EGFR C797S mutation in T790M-deficient patients include MET amplification, HER2 amplification, aberrant FGFR signaling, BRAF mutation, abnormal activation of IGF1R, and downstream signaling pathway activation (RAS/RAF/MEK/ERK and PI3K/AKT/mTOR) 3, 4. These pathways drive cellular survival, proliferation, motility, migration, invasion, angiogenesis, and epithelial-mesenchymal transition (EMT), providing bypass pathways in the presence of EGFR inhibitors 5, 6.

**Figure 1:**
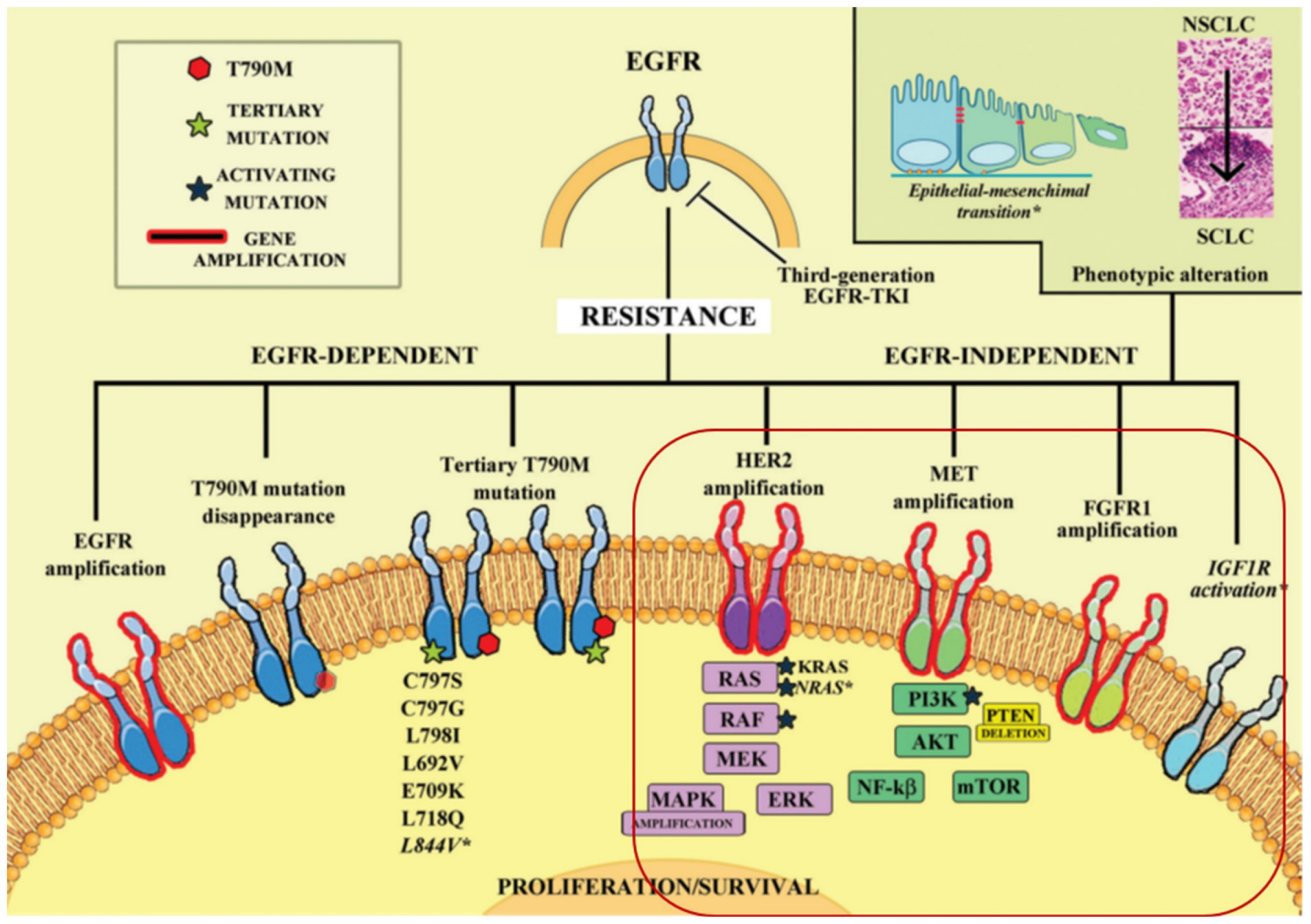
** The complex landscape of resistance mechanisms to third-generation EGFR-TKIs, such as osimertinib, in the treatment of NSCLC^3^, ^4^.** Osimertinib effectively targets EGFR-dependent resistance mechanisms, particularly those involving EGFR T790M and other tertiary mutations. However, its efficacy is limited by the emergence of mutations like C797S and by EGFR-independent pathways. IL-12's immunomodulatory effects could complement osimertinib by potentiating the immune system's ability to recognize and destroy cells that have developed these bypass resistance pathways.

We investigate IL-12's potential to modulate bypass pathways and enhance osimertinib's efficacy (Figure 1). This immunomodulatory cytokine could alter the tumor microenvironment from 'non-inflammatory' to 'inflammatory', driving immune cell infiltration and activity to reverse osimertinib resistance and improve tumor sensitivity.

EGFR mutations alter the immune microenvironment of lung adenocarcinoma. EGFR-mutant lung adenocarcinomas exhibit a unique "non-inflammatory" tumor microenvironment characterized by low immune cell infiltration, including fewer T cell infiltrates, increased Treg numbers, and elevated PD-L1 expression. Heterogeneity is observed between different mutation subtypes and EGFR mutation abundance (VAF) in the tumor microenvironment, reflected in the type and quantity of infiltrating immune cells 7. Osimertinib resistance due to EGFR mutations may be associated with mutation-induced immunosuppressive microenvironments. Studies suggest that EGFR-TKIs induce interferon response in EGFR-mutant NSCLC, highlighting the importance of immune activation for the therapeutic efficacy of EGFR-TKIs.

IL-12 can alter the local immune microenvironment of tumors, favoring the maintenance of a sustained immune response 8. IL-12 may particularly enhance the IFN-γ response and influence key survival pathways such as RAS/RAF/MEK/ERK and PI3K/AKT/mTOR, bolstering the immune attack against resistant tumor cells. The expected synergy between IL-12 and osimertinib aims not only to suppress EGFR-dependent growth but also to forge a potent immune response, targeting multiple resistance mechanisms for a sustained clinical benefit.

To address this, we selected osimertinib-resistant NSCLC (H1975(L858R/T790M/C797S)) as an immunosuppressive model and osimertinib-sensitive NSCLC (H1975(L858R/T790M)) as an immune-inflammatory model 9-11. Our research intends to clarify how IL-12 can recalibrate the immune landscape within tumors and amplify the effect of osimertinib against resistance, thereby advocating for a novel combinatory approach in NSCLC therapy.

## Materials and methods

### Antibodies and reagents

Osimertinib tablets (Tagrisso) was obtained from AstraZeneca AB, Murine IL-12 p70 was purchased from PeproTech. XenoLightTM D-Luciferin Potassium Salt was purchased from PerkinElmer. Antibodies against NCAM1, CD68, CD45, CD86, Ly6c, CD11b, IL-12A, and VEGFR1 were purchased from Abcam. Antibodies against CD206/MRC1, p-mTOR, mTOR, PI3K, AKT, NF-κB, MAPK, and TGF-β were purchased from Cell signaling. Antibodies against Granzyme B and IFN-γ were purchased from Immunoway. Antibody against β-actin was purchased from Proteintech. 0.25% Tripsin-EDTA and DAPI were purchased from ThermoFisher. 4% Paraformaldehyde Fix Solution, EDTA Antigen Retrieval solution (pH 8.0) were from Servicebio. Protein protease inhibitor and Phosphatase Inhibitor Cocktail were from Roche.

### Cancer cell lines and cell culture

The human non-small cell lung cancer cell line H1975 (L858R/T790M/C797S) was sourced from Nanjing KeyGen Biotech Co., Ltd., and H1975 (L858R/T790M) was obtained from Cell Bank of the Chinese Academy of Sciences. The cells were cultured in RPMI 1640 supplemented with 10% FBS, penicillin (100 IU/ml), and streptomycin (100 mg/ml) at 37°C in an incubator containing 5% CO_2_.

### Mice

Male BALB/c nu/nu mice (SPF degree, 6 weeks old, 18-20 g weight) were purchased from Beijing Vital River Laboratory Animal Technology Co., Ltd. Animals were housed with a cycle of 12-hours light and 12-hours dark. All procedures were approved by the Committee of Experimental Animals of School of Medicine and Pharmacy, Ocean University of China (OUCSMP-20200701) and conformed to Guide for Care and Use of Laboratory Animals published by the United States National Institutes of Health (NIH Publication No 85-23, revised 1996).

### Murine tumor models

Human non-small cell lung cancer cells, H1975 (L858R/T790M/C797S) or H1975 (L858R/T790M), were inoculated into the axilla of BALB/c Nude mice at a density of 0.2 mL per mouse (5*10^6 cells). When the tumors reached approximately 1000 mm³, the animals were sacrificed, and the tumor tissues were dissected. The tumors were then cut into 2-3 mm³ fragments and implanted subcutaneously into nude mice using a catheter for further passages. At the completion of tumor inoculation, the body weight of each mouse was measured, and the mice were randomly assigned group numbers, with 8 mice per group.

Osimertinib was administered orally by gavage five times per week (consecutively), while IL-12 was delivered by subcutaneous injection twice a week (with intervals); both treatments lasted for 3 weeks. The negative control group received physiological saline, administered in the same manner as the experimental group, with subcutaneous injections twice a week and oral gavage five times a week.

During the experiment, tumor growth, animal behavior, and diet were observed before each administration. The average tumor volume for each group started at around 100 mm³ before the first administration (Day 1). Subsequently, animal body weight and tumor volume (V=1/2×length×width²) were measured twice a week. At the end of the experiment, animals were dissected to measure tumor weight (IRTW (%) = (W _NC_ - W _treatment_) / W _NC_ × 100%) and spleen index (Spleen index (%) = Spleen weight (g) / Mouse body weight (g) × 100%).

### Immunofluorescence analysis

Paraffin-embedded tissue sections were deparaffinized in 2 changes of xylene and rehydrated in serial decreasing concentrations of ethanol (100%, 85% and 75%). The sections were then immersed in EDTA antigen retrieval buffer (pH 8.0) (Servicebio, G1206) and held at sub-boiling point temperature for 8 min twice. After washing three times with PBS (pH 7.4) in a Rocker device (Servicebio, TSY-B), the sections were further covered to block non-specific binding in 3% BSA for 30 min, followed by incubation overnight at 4°C in a wet box with primary antibody CD8 alpha or LAMP1 prepared in PBS at a certain ratio (1:500).

### Immunohistochemistry and HE analysis

IHC was performed by Bioscience Solutions Group using the avidin-biotin complex (ABC) method. Hematoxylin and eosin (H&E) staining was also performed.

### Western blot analysis

Cells were harvested in RIPA buffer. Protein lysates were separated by SDS-PAGE and transferred to nitrocellulose membrane (GE Healthcare). The membranes were probed with primary antibodies and then incubated with horseradish peroxidase-conjugated secondary antibodies. Immune complexes were detected with an Immobilon TM western chemiluminescence HRP substrate (Millipore) and photographed on a Tanon 5200 imaging system.

## Results

### Tumor Inhibition Validation

**Figure 2:**
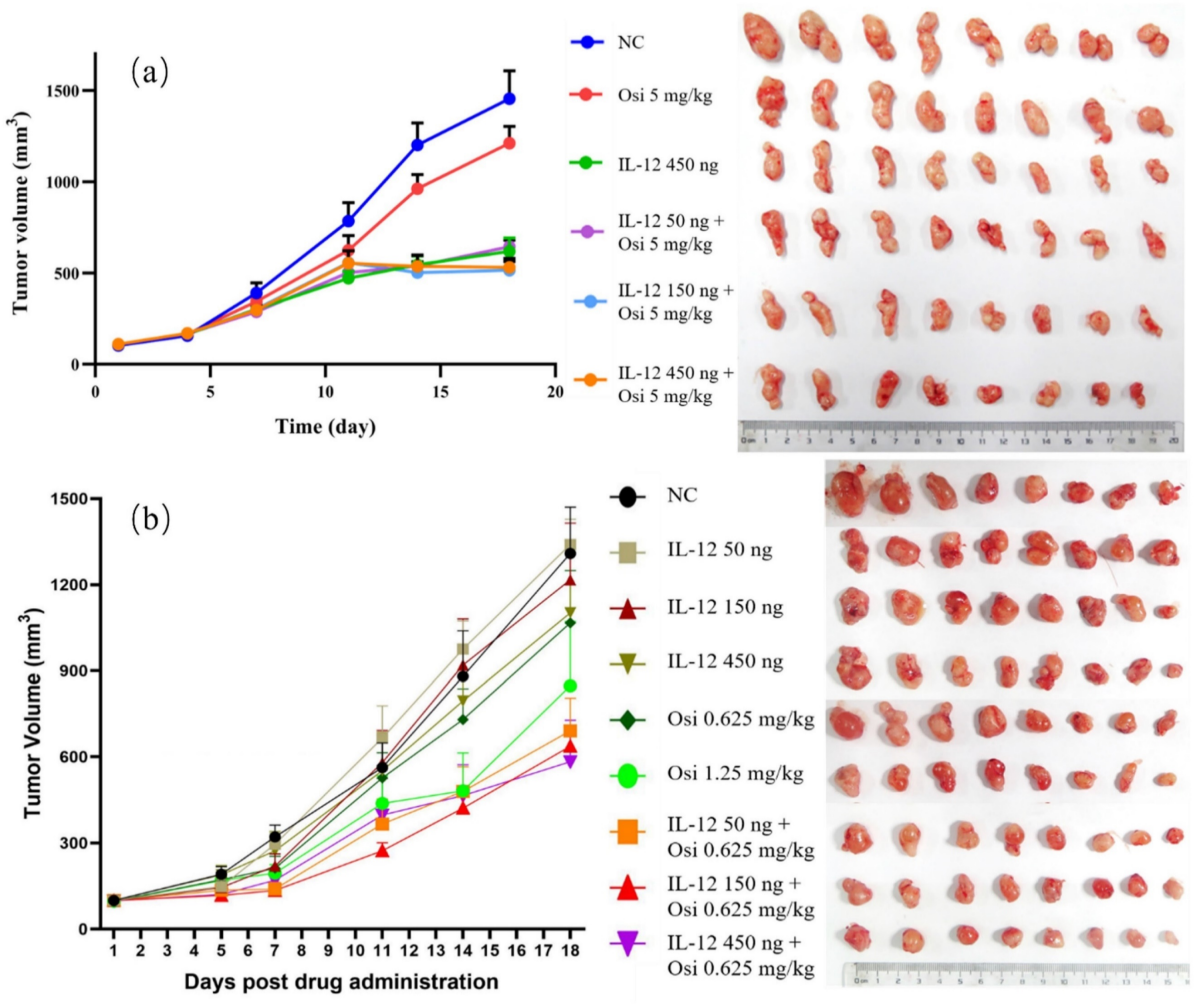
Growth Trends of Average Tumor Volume in Different Groups of Osi- Resistant NSCLC Models (a) and OSi- Sensitive NSCLC Models (b), Alongside Photographic Documentation of Tumor Progression in Each Group.

In the tumor immune suppression model, specifically the osimertinib-resistant strain (H1975(L858R/T790M/C797S)) of NSCLC, the monotherapy with osimertinib exhibited a tumor inhibition rate of 21.99%, showing limited antitumor effects. Monotherapy with rmIL-12 demonstrated a more pronounced antitumor effect with a tumor inhibition rate of 58.97%. The combination groups at low, medium, and high doses all exhibited significant antitumor effects, with inhibition rates of 55.99%, 51.57%, and 61.86%, respectively. The differences were statistically significant when compared to the osimertinib monotherapy group (P< 0.05). The tumor volume trends for each group are depicted in Figure 2, and the statistical data for average tumor weight and inhibition rates are summarized in Table 1.

In the immunoinflammatory model of NSCLC using Osimertinib-sensitive strain (H1975(L858R/T790M)), at Day 7, both the low-dose and medium-dose combination groups exhibited a significant reduction in tumor volume compared to the NC group (P< 0.05). After the experiment (Day 18), the combination therapy groups showed an increased tumor inhibition rate compared to the Osimertinib monotherapy group. The tumor volume inhibition rates for the combination low, medium, and high-dose groups were 46.8%, 52.6%, and 55.2%, respectively, while Osimertinib low and high-dose groups were 20.7% and 40.8%. Additionally, at the end of the experiment (Day 18), the IL-12 medium and high-dose groups demonstrated some tumor volume suppression in the sensitive H1975 strain, with inhibition rates of 19% and 25.7% compared to the negative control group. Notably, there was a significant synergistic effect when IL-12 was combined with Osimertinib. The tumor volume trends for each group are illustrated in Figure 2, and the average tumor weight and inhibition rates are summarized in Table 2.

Compared to the NC group, both the Osimertinib group and the combination therapy group showed a decrease in spleen index in mice (Table 2). The enlargement of spleens in the NC group is directly related to the increased tumor burden, which results in the retention of various immune cells in the spleen. Following combination therapy, a reduction in spleen index was observed in mice, indicating a potential restoration of immune cell retention in the spleens of tumor-bearing mice. This aligns with the consistent decrease in tumor burden observed in the combination treatment group of mice.

Under the given dosing frequency and dosage in this experiment, the anticancer efficacy of Osimertinib in combination with IL-12 is markedly superior to Osimertinib monotherapy. Notably, the low-dose combination of Osimertinib and IL-12 demonstrates superior efficacy compared to the high-dose Osimertinib group. This provides a theoretical basis for reducing the clinical dosage of Osimertinib, suggesting that the use of Osimertinib in combination with IL-12 may mitigate the toxicity and resistance issues associated with high-dose Osimertinib.

### IL-12 in combination with Osimertinib promotes immune cell infiltration in tumor tissues

**Figure 3:**
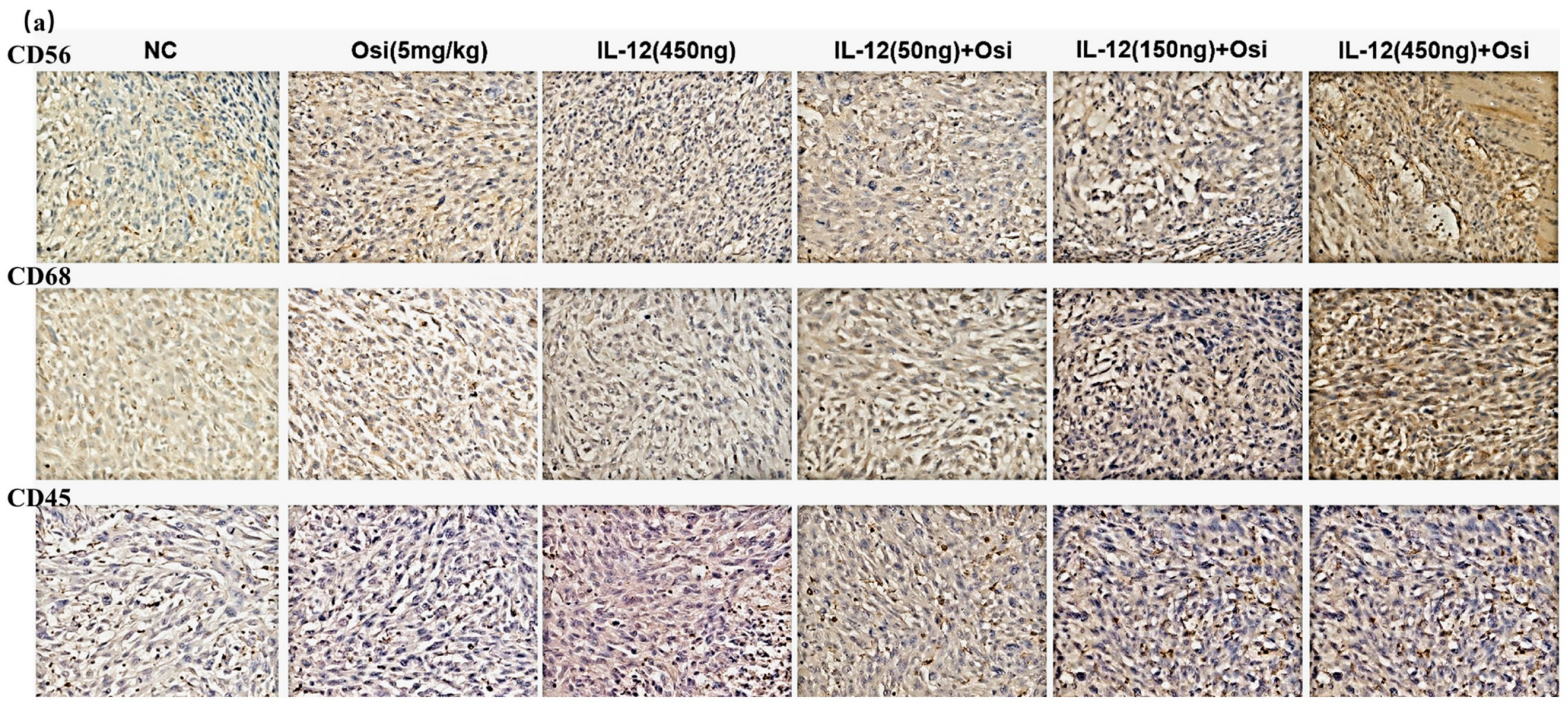

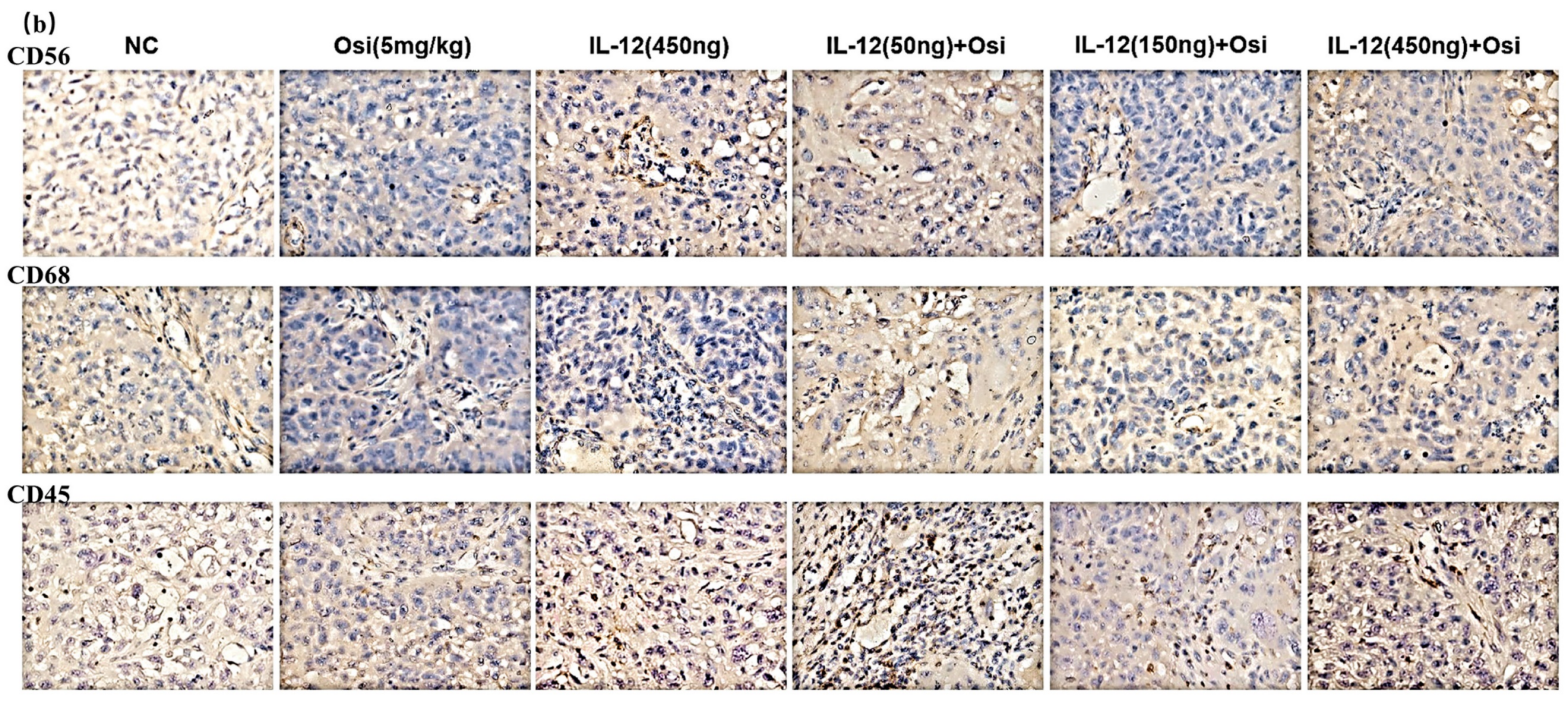

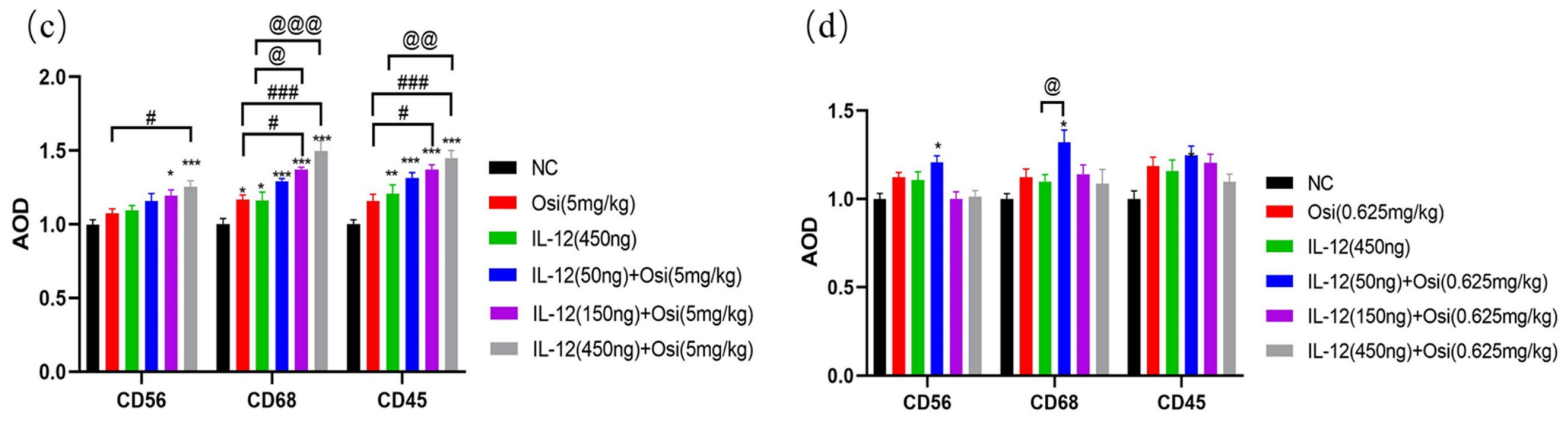
** Immunohistochemical analysis of immune cell infiltration in tumor tissues of H1975 (L858R/T790M/C797S) tumor-bearing mice (a, c) and H1975 (L858R/T790M) xenograft mice (b, d).** (a, b) Immunohistochemical staining images showing immune cell infiltration within tumor tissues. Hematoxylin (purple) stains the nuclei of all viable cells, while the brown staining represents the specific immune cell markers CD56, CD68, and CD45.CD56 is a marker for natural killer (NK) cells and a subset of T cells. CD68 identifies macrophages and monocytes. CD45 labels leukocytes in general. The images demonstrate varying expression of these markers across different treatment groups, including the control (NC), Osimertinib alone, different doses of IL-12, and combinations of IL-12 with Osimertinib. The immune cell infiltration is visibly higher in combination treatment groups, indicating enhanced immune activity within the tumor microenvironment. The images were analyzed using a Leica Aperio AT2 imaging system. (c, d) Quantitative bar charts representing the average optical density (AOD) of CD56, CD68, and CD45 markers. The data were obtained using the imaging system and quantify the immune cell infiltration seen in panels (a) and (b). Statistical significance: The bar charts show statistical comparisons between the treatment groups. Note: *, **, ***, Compared with the NC group, P < 0.05, P < 0.01, P < 0.001; #, ###, Compared with the osimertinib monotherapy group, P < 0.05, P < 0.001; @, @@, @@@, Compared with the IL-12 monotherapy group, P < 0.05, P < 0.01, P < 0.001.

Immunohistochemical analysis was used to assess the infiltration levels of NK cells (CD56+), macrophages (CD68+), and leukocytes (CD45+) in Osimertinib-resistant NSCLC tissues. As shown in Figure 3, the tumor tissues in the NC group exhibited minimal immune cell infiltration. Osimertinib monotherapy and IL-12 monotherapy each showed a certain degree of promotion of immune cell infiltration (CD68+, compared to the NC group, P < 0.05). Notably, the co-administration of Osimertinib and IL-12 demonstrated a robust enhancement of CD56+ NK cells, CD68+ macrophages, and CD45+ leukocytes infiltration, displaying a dose-dependent correlation. Particularly, the combination of IL-12 at medium and high doses exhibited strong activation of immune cells (CD56+, CD68+, and CD45+) infiltrating tumor tissues.

The comparison of CD56+ NK cell infiltration in tumor tissues revealed increased ratios in each treatment group compared to the NC group, with percentages as follows: Osimertinib group, 7.3%; IL-12 group, 9.4%; low-dose combination group, 15.7%; medium-dose combination group, 19.5%; high-dose combination group, 25.5%; P < 0.05. Additionally, when compared to the Osimertinib group, the high-dose combination group showed a significant increase (P < 0.05). The assessment of CD68+ macrophage infiltration in tumor tissues demonstrated increased ratios in each treatment group compared to the NC group, with percentages as follows: Osimertinib group, 16.6%; IL-12 group, 16%; low-dose combination group, 29.2%; medium-dose combination group, 37.2%; high-dose combination group, 49.7%; P < 0.001. Furthermore, compared to the Osimertinib group, the medium-dose, and high-dose combination groups showed significant increases (P < 0.05), and compared to the IL-12 group, the medium-dose, and high-dose combination groups exhibited significant increases (P < 0.05).The evaluation of CD45+ leukocyte infiltration in tumor tissues indicated increased ratios in each treatment group compared to the NC group, with percentages as follows: Osimertinib group, 15.8%; IL-12 group, 21%; low-dose combination group, 31.7%; medium-dose combination group, 37.2%; high-dose combination group, 45%; P < 0.01. Moreover, compared to the Osimertinib group, the medium-dose and high-dose combination groups showed significant increases (P < 0.05), and compared to the IL-12 group, the high-dose combination group exhibited a significant increase (P < 0.01).

Comparative analysis of immune cell infiltration in Osimertinib-sensitive NSCLC tissues, particularly NK cells (CD56+), macrophages (CD68+), and leukocytes (CD45+), was conducted, and the results are presented in Figure 3. Tumor tissues from the control group, Osimertinib monotherapy group, and IL-12 monotherapy group all exhibited minimal immune cell infiltration. Notably, the combined administration of Osimertinib and low-dose IL-12 demonstrated a substantial promotion of immune infiltration for NK cells, macrophages, and leukocytes in tumor tissues.

When compared to the NC group, the increase ratios of CD56+ NK cell infiltration for each treatment group were as follows: Osimertinib group 12.3%, IL-12 group 10.8%, combined low-dose group 20.8%, combined medium-dose group 0%, combined high-dose group 1.2%. The increase ratios for CD68+ macrophage infiltration were: Osimertinib group 12.4%, IL-12 group 9.7%, combined low-dose group 32%, combined medium-dose group 14%, combined high-dose group 8.7% (significant compared to the IL-12 group, P<0.05). The increase ratios for CD45+ leukocyte infiltration were: Osimertinib group 18.7%, IL-12 group 15.6%, combined low-dose group 24.7%, combined medium-dose group 20.3%, combined high-dose group 9.7%. This suggests that in Osimertinib-sensitive tumor tissues, IL-12 similarly promotes immune cell infiltration.

### IL-12 combined with Osimertinib promotes infiltration of myeloid-derived white blood cells and M1-type macrophages in immune-suppressive tumor tissue

**Figure 4:**
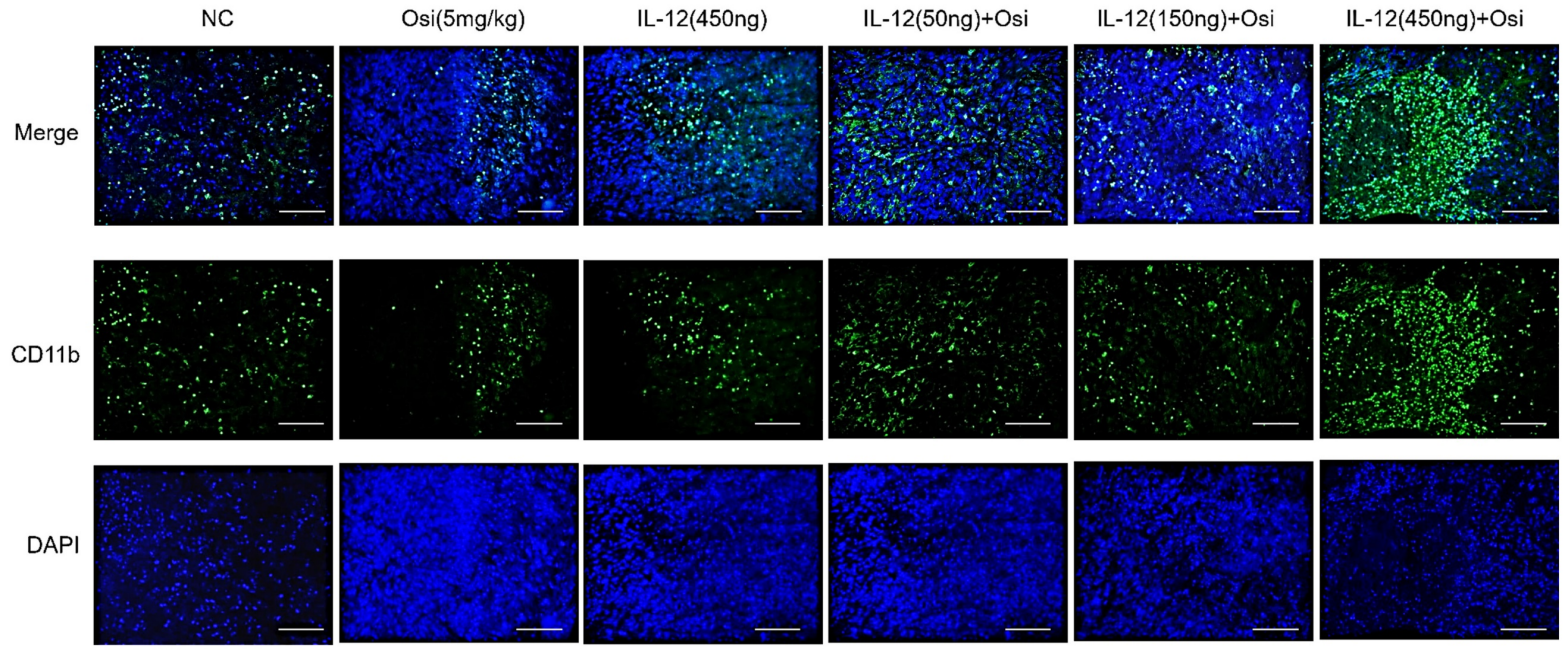

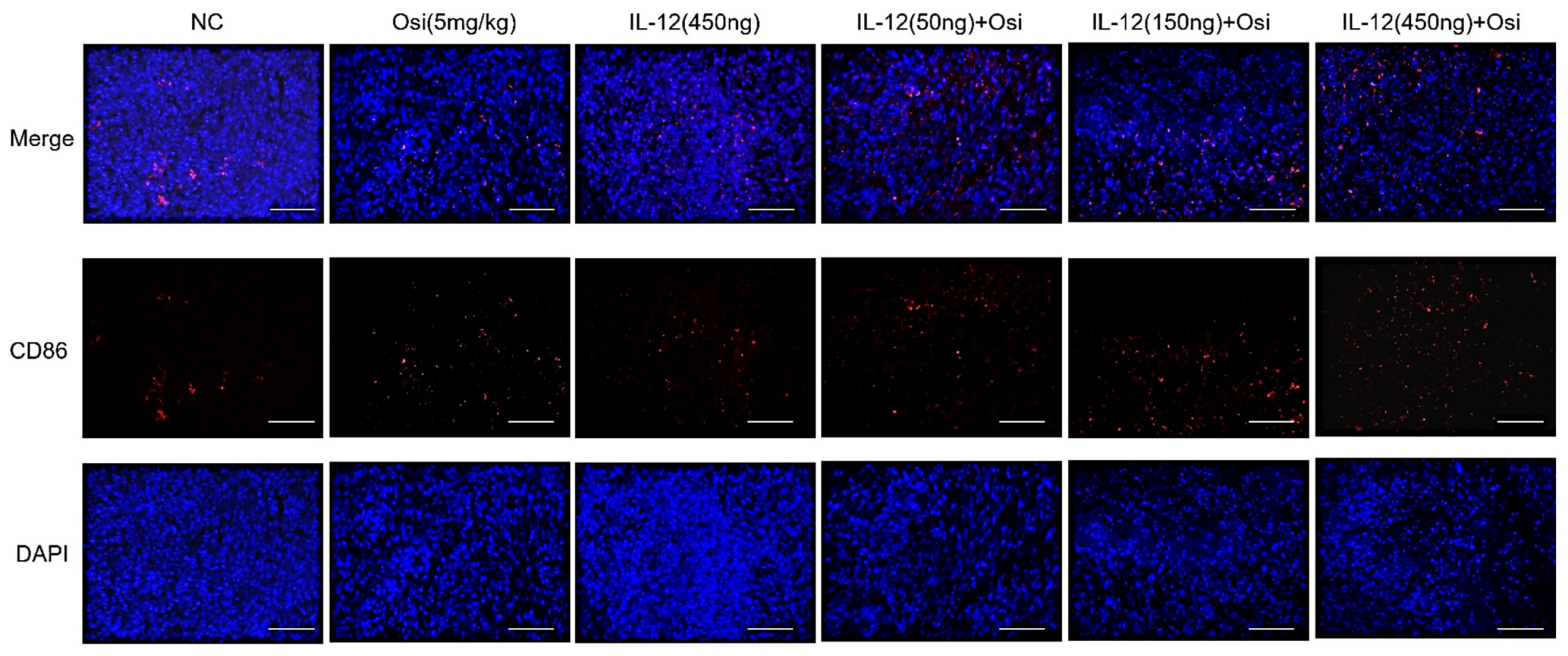

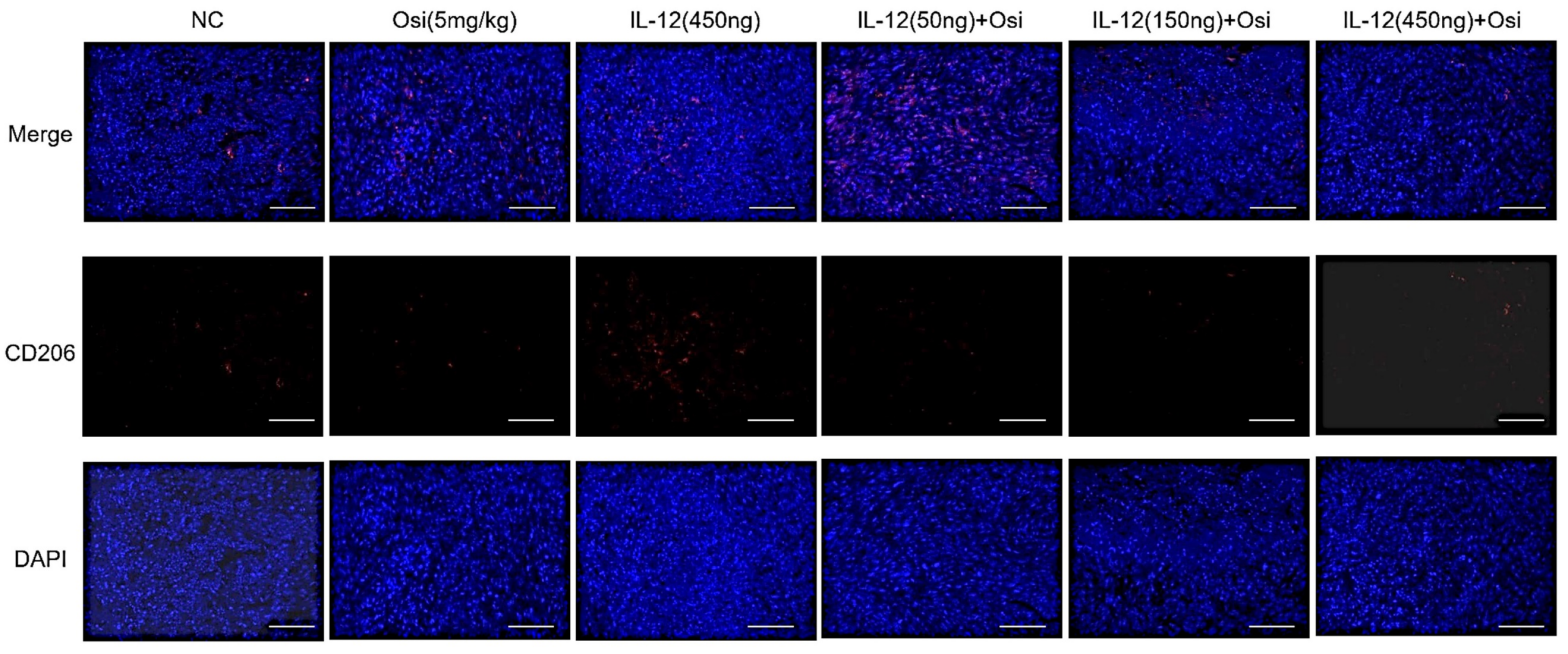

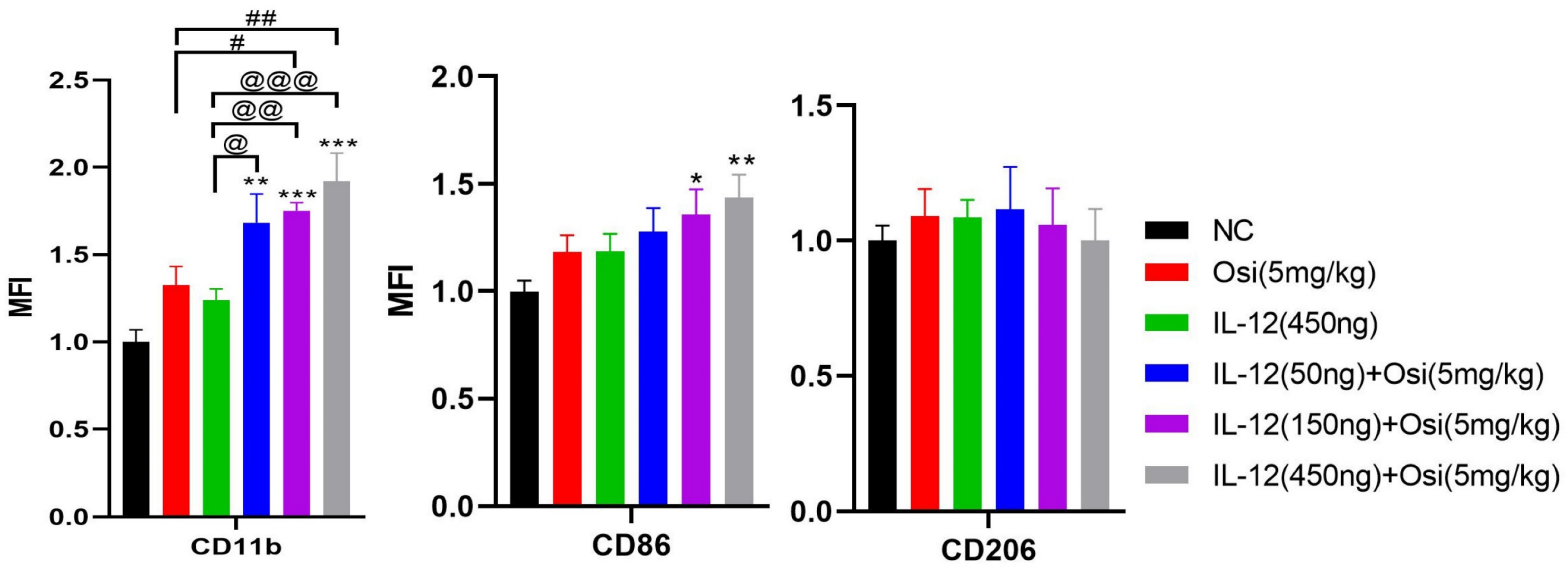
** Immunofluorescence analysis of immune cell infiltration in tumor tissues of H1975 (L858R/T790M/C797S) xenograft mice.** The staining illustrates the infiltration of CD11b+ myeloid-derived white blood cells, CD86+ M1 macrophages, and CD206+ M2 macrophages in different treatment groups. Cells positive for the CD11b, CD86, and CD206 markers appear in green (fluorescein isothiocyanate or FITC) or red (tetramethylrhodamine or TRITC), while nuclei are stained blue (DAPI). The "Merge" row displays the overlay of fluorescence signals with DAPI-stained nuclei. CD11b+ myeloid-derived cells are green, and nuclei are blue. The combination of Osimertinib and IL-12 leads to significantly increased infiltration compared to single-agent or control groups. The CD86 row shows red fluorescence representing M1 macrophages. Combination therapy enhances M1 macrophage infiltration, indicating an inflammatory response. The CD206 row represents M2 macrophages in red. M2 macrophages do not show significant increases across different treatment groups. The bar chart quantifies the mean fluorescence intensity (MFI) of CD11b+ cells using the ImageJ analysis system. Combination therapy shows significantly higher CD11b+ cell infiltration, indicating a strong immune response. The MFI bar chart for CD86+ cells also shows a dose-dependent increase, suggesting an enhanced presence of M1 macrophages, which contribute to a pro-inflammatory and tumoricidal environment. The bar chart of CD206 does not show significant differences, reflecting that M2 macrophage levels remain relatively stable across treatment groups. Note: Scale bar, 200μm. *, **, ***: Compared to the NC group, P < 0.05, P < 0.01, P < 0.001; #, ##: Compared to the Osimertinib group, P < 0.05, P < 0.01; @, @@, @@@: Compared to the IL-12 group, P < 0.05, P < 0.01, P < 0.001.

M1 macrophages possess robust antigen-presenting capabilities and induce strong Th1 responses 12; hence, the presence of M1-type TAMs is associated with anti-tumor activity. Conversely, M2 macrophages play a crucial role in limiting immune responses, inducing angiogenesis, and promoting tissue repair. Therefore, the presence of M2-type TAMs is associated with pro-tumor activity. The infiltration levels of myeloid-derived white blood cells (CD11b+), M1-type macrophages (CD86+), and M2-type macrophages (CD206+) in tumor tissue were assessed using immunofluorescence analysis, as shown in Figure 4. The tumor tissues in the NC group exhibited relatively low infiltration of myeloid-derived white blood cells (CD11b+) and M1-type macrophages (CD86+). Single-agent use of Osimertinib or IL-12 moderately promoted the infiltration of these immune cells (P > 0.05). However, co-administration of Osimertinib and IL-12 significantly increased the quantity of myeloid-derived white blood cells and M1-type macrophages in tumor tissues, exhibiting a dose-dependent relationship. The increase ratios of CD11b+ myeloid-derived white blood cell infiltration in each treatment group compared to the NC group were as follows: Osimertinib group, 32.6%; IL-12 group, 23.9%; co-administration low-dose group, 68.3%; co-administration medium-dose group, 75.2%; co-administration high-dose group, 92%, P < 0.01. When compared to the Osimertinib group, the co-administration medium-dose and high-dose groups showed significant differences, with P < 0.05. Compared to the IL-12 group, there were significant differences (P < 0.05). The increase ratios of CD86+ M1-type macrophage infiltration in each treatment group compared to the NC group were as follows: Osimertinib group, 18.2%; IL-12 group, 18.6%; co-administration low-dose group, 27.7%; co-administration medium-dose group, 35.5%; co-administration high-dose group, 43.7%. The co-administration medium-dose and high-dose groups showed significant differences compared to the IL-12 group (P < 0.05). The quantity of M2-type macrophages in the tumor tissue was relatively low, and there were no significant differences among the treatment groups compared to the NC group (P > 0.05).

### IL-12 combined with Osimertinib inhibits MDSCs and their subpopulations in tumor tissues

**Figure 5:**
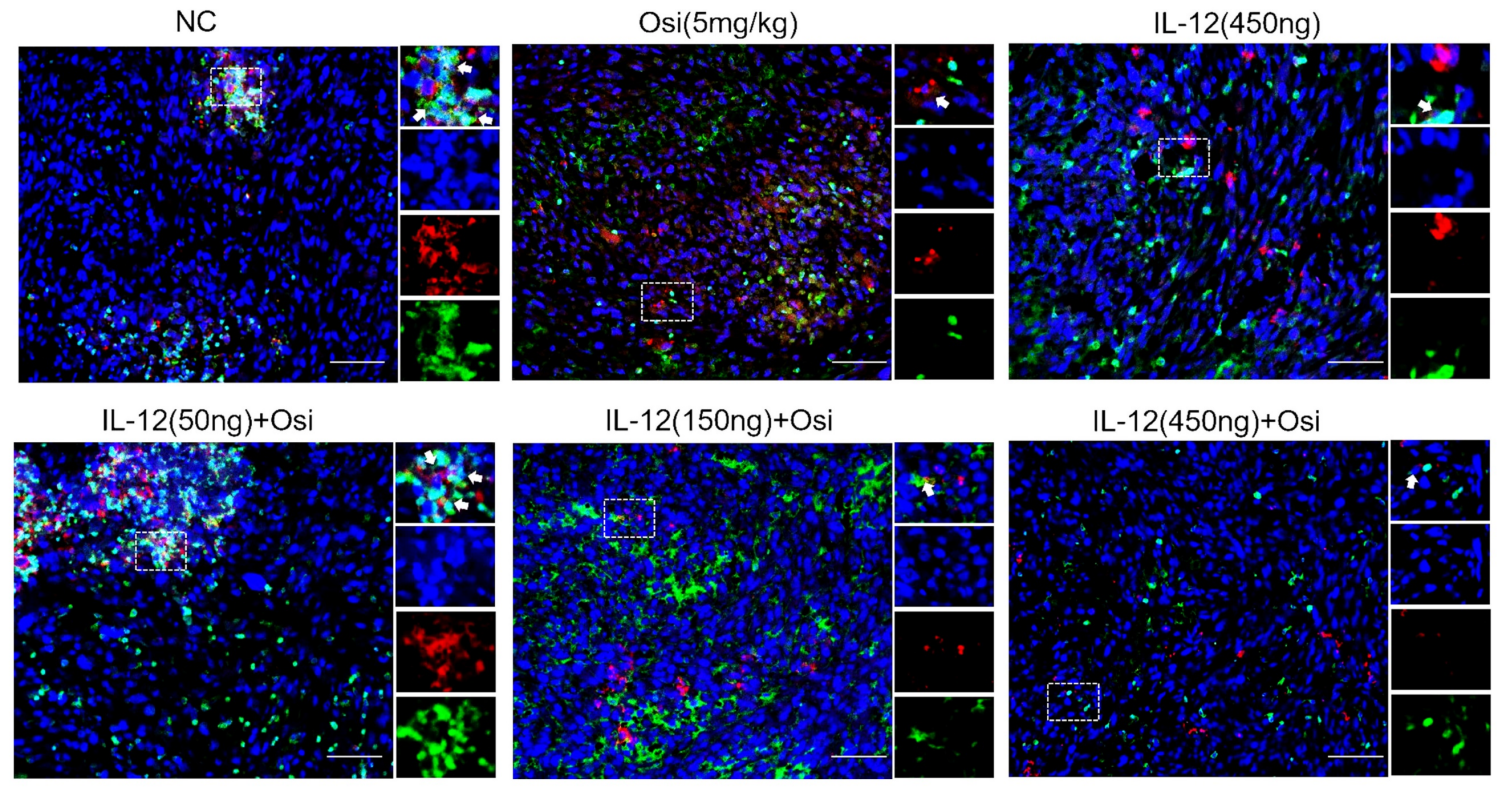
** Immunofluorescence analysis of MDSCs infiltration in tumor tissues of H1975 (L858R/T790M/C797S) xenograft mice.** CD11b+ myeloid-derived suppressor cells (MDSCs) are labeled with green fluorescence (FITC), while GR-1+ cells are labeled with red fluorescence (TRITC). In the control group, substantial infiltration of CD11b+GR-1+ MDSCs is observed (indicated by white arrows). Treatment with either Osimertinib or IL-12 alone shows a moderate reduction in MDSC infiltration. Combination therapy with Osimertinib and IL-12 significantly suppresses MDSC infiltration into tumor tissues, especially in the medium and high-dose combination groups, where MDSCs are almost entirely absent under immunofluorescence.

**Figure 6:**
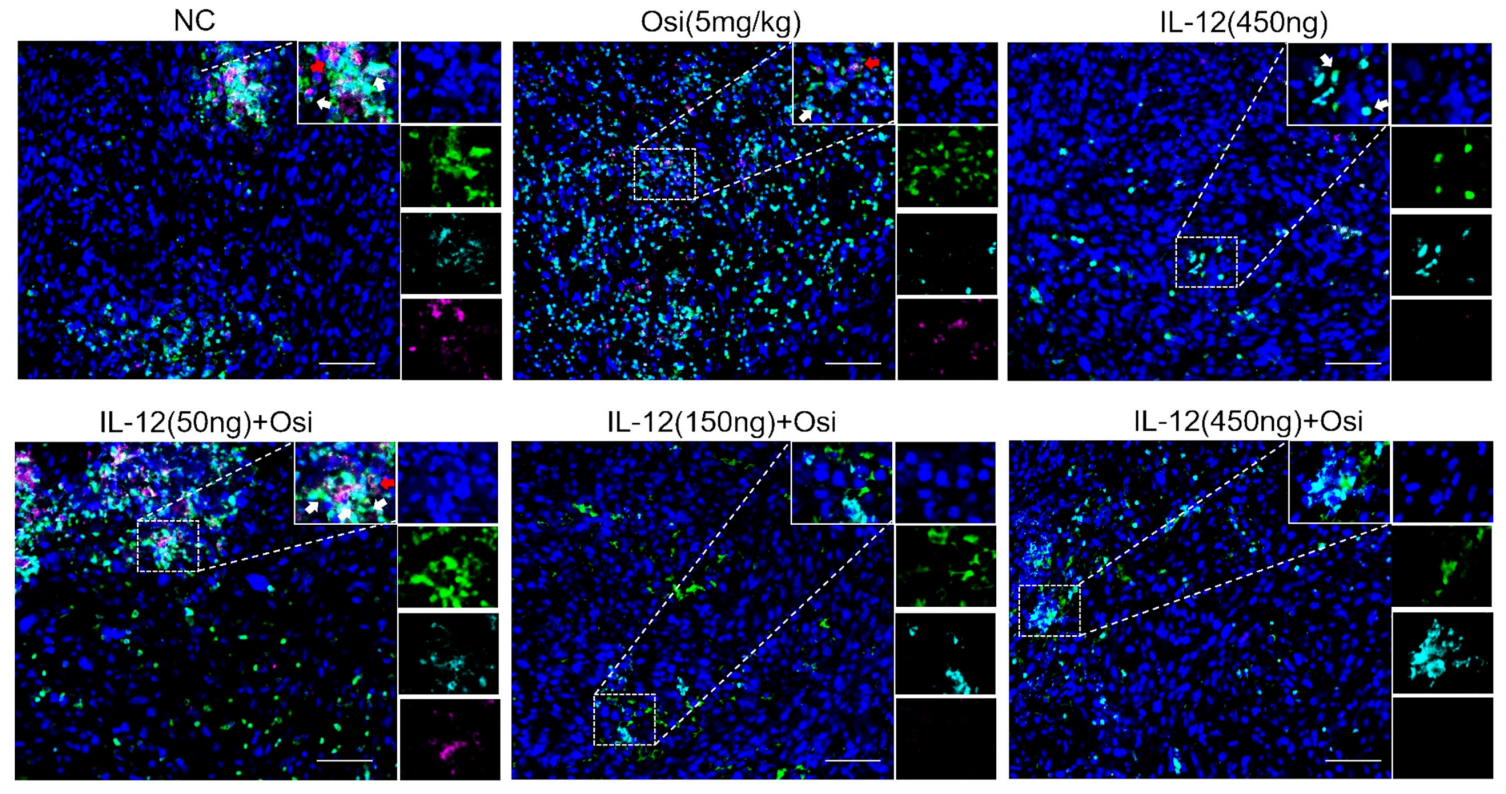
** Immunofluorescence analysis of infiltrated monocytic MDSCs (CD11b+Ly6C+Ly6G-, indicated by white arrows) and granulocytic MDSCs (CD11b+Ly6C-Ly6G+, indicated by red arrows) in tumor tissues of H1975 (L858R/T790M/C797S) xenograft mice.** CD11b+ cells are labeled with green fluorescence (FITC), Ly6C+ monocytic MDSCs with indigo fluorescence (Cy5), and Ly6G+ granulocytic MDSCs with magenta fluorescence (Cy3). In the control group, abundant infiltration of both monocytic and granulocytic MDSCs is seen (white and red arrows, respectively). Osimertinib or IL-12 alone shows some suppression of these MDSC subpopulations, but combination therapy significantly inhibits their infiltration into tumor tissues. Particularly in the medium and high-dose combination groups, no monocytic or granulocytic MDSCs are detected under immunofluorescence staining, indicating robust suppression.

Due to the enrichment and activation of MDSCs being a common feature of malignant tumors 13, we analyzed inhibitory cells of myeloid origin (MDSCs, CD11b+GR-1+) and their subgroups (monocytic MDSCs, CD11b+Ly6C+Ly6G- and granulocytic MDSCs, CD11b+Ly6C-Ly6G+) in tumor tissues. The immunofluorescence results are shown in Figures 5-6. Tumor tissues in the NC group showed a significant infiltration of the target cells (indicated by arrows). Monotherapy with Osimertinib and IL-12 exhibited some inhibitory effects, while the combined use of Osimertinib and IL-12 significantly inhibited the infiltration of myeloid-origin inhibitory cells (MDSCs, CD11b+GR-1+) and their subgroups into tumor tissues. Particularly in the combined medium-dose and high-dose groups, no MDSCs (Figure 5) and their subgroups (Figure 6, monocytic MDSCs, and granulocytic MDSCs) were observed using immunofluorescence.

In summary, EGFR mutations lead to alterations in the immune microenvironment of lung adenocarcinoma, resulting in a distinct "non-inflammatory" tumor microenvironment with low immune cell infiltration, contributing to Osimertinib resistance. IL-12, by increasing immune cell infiltration and reducing the suppressive effects of MDSCs in tumor tissues, improves the tumor microenvironment and restores the sensitivity of tumor cells to Osimertinib. Therefore, the combined use of IL-12 and Osimertinib demonstrates a potent inhibitory effect on H1975 (L858R/T790M/C797S) mouse tumors.

### The impact of IL-12 combined with osimertinib on immune cell cytokines and effector molecules in tumor tissue

**Figure 7:**
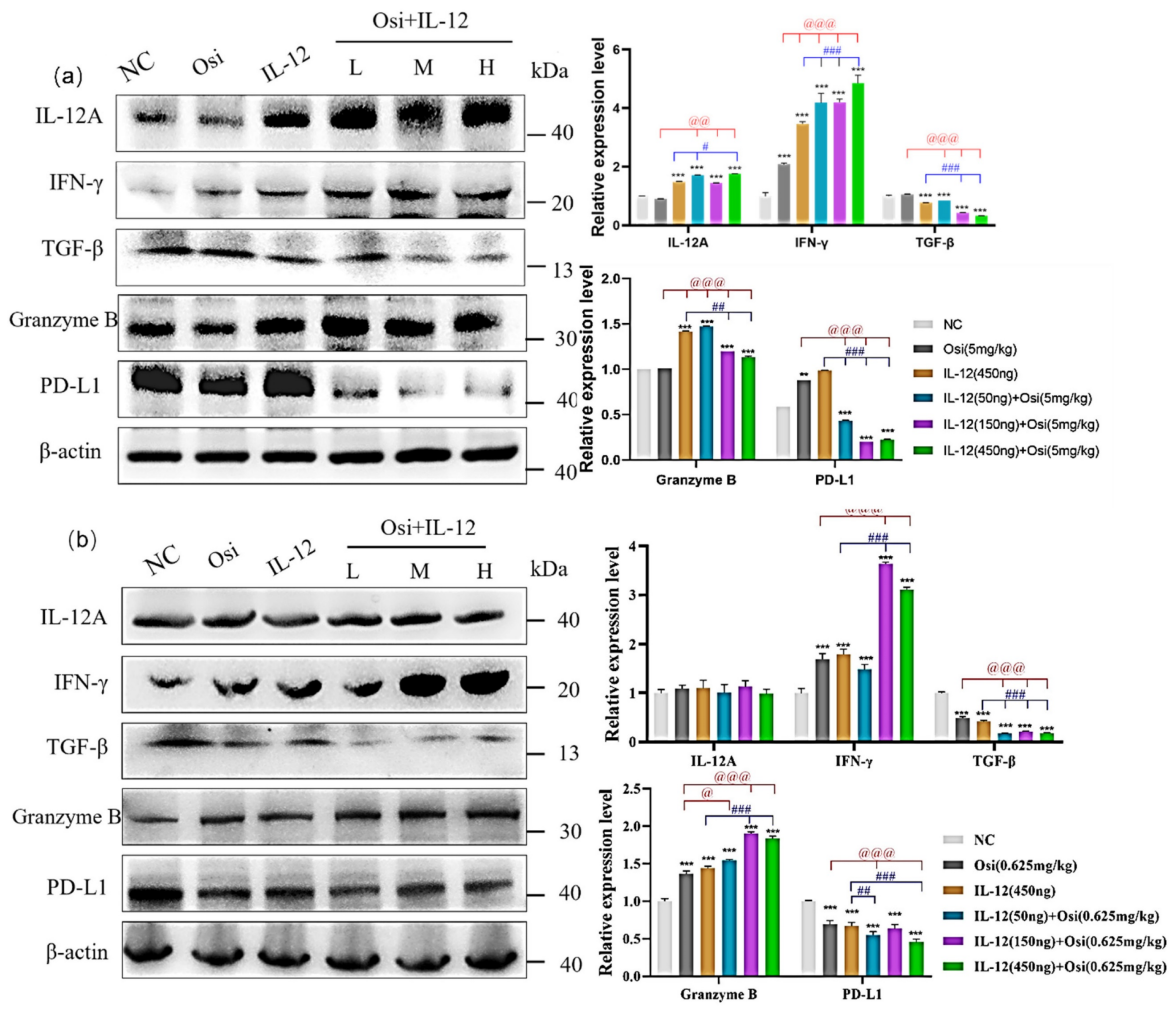
** Results of immune cell factor expression in tumor tissues of xenograft mice. (a) represent the results in the H1975 (L858R/T790M/C797S) tumor model, while (b) show the results in the H1975 (L858R/T790M/C797S) tumor model.** Note: NC, solvent control group; Osi, Osimertinib group; L, combination low-dose group; M, combination medium-dose group; H, combination high-dose group; **, ***: Compared to the solvent control group, p < 0.05, p < 0.001; #, ##, ###: Compared to the IL-12 monotherapy group, p < 0.05, p < 0.01, p < 0.001; @, @@, @@@: Compared to the Osimertinib monotherapy group, p < 0.05, p < 0.01, p < 0.001.

To further investigate the improvement of the immunosuppressive microenvironment by IL-12 combined with Osimertinib, we analyzed the expression changes of immune cell factors and effector molecules in tumor tissues. In the H1975 (L858R/T790M/C797S) immunosuppressive model (Figure 7-(a)), tumor tissues exhibited lower expression of IL-12A. Both IL-12 monotherapy and the combination treatment significantly increased IL-12A expression: compared to the Osimertinib group, the combination low, medium, and high-dose groups showed p < 0.01; compared to the IL-12 group, the combination low-dose group and high-dose group showed p < 0.05.

Tumor tissues expressed a certain amount of IFN-γ. Compared to the NC group, IL-12 or Osimertinib monotherapy, as well as the combination therapy, significantly promoted the expression of IFN-γ in tumor tissues (p < 0.001). Compared to the Osimertinib group, the IL-12 monotherapy group and the combination therapy group significantly promoted IFN-γ expression (p < 0.001). Compared to the IL-12 group, the combination therapy group also significantly increased IFN-γ expression (p < 0.001).

Additionally, a certain amount of TGF-β was expressed in cancer tissues. The IL-12 group significantly inhibited the expression of TGF-β in tumor tissues, compared to the NC group (p < 0.001). IL-12 combined with Osimertinib also significantly inhibited TGF-β: compared to the NC group or the Osimertinib group, all three combination therapy groups showed p < 0.001; compared to the IL-12 monotherapy group, the combination medium and high-dose groups showed p < 0.001.

The results in Figure 7(a) show that the tumor tissues of the NC group and the Osimertinib group expressed a certain amount of Granzyme B. The IL-12 group and the combination therapy group both significantly promoted the expression of Granzyme B in tumor tissues (compared to the NC group, p < 0.001; compared to the Osimertinib group, p < 0.001; compared to the IL-12 group, the combination medium and high-dose groups showed p < 0.001). At the same time, the tumor tissues of the NC group, Osimertinib group, and IL-12 monotherapy group all expressed a large amount of PD-L1. The combination of Osimertinib and IL-12 significantly inhibited the expression of PD-L1 (compared to the NC or monotherapy groups, p < 0.01).

IL-12 combined with Osimertinib also affected the expression of immune cell factors and effector molecules in H1975 (L858R/T790M) tumor tissues. In contrast to the results in Osimertinib-resistant tumors, we did not observe changes in IL-12A after combined treatment in Osimertinib-sensitive H1975 mouse tumors (Figure 7-(b)). However, IL-12 combined with Osimertinib significantly increased the expression levels of IFN-γ (combination medium and high-dose groups vs. Osimertinib or IL-12 monotherapy, all p < 0.001) and Granzyme B (combination three dose groups vs. Osimertinib or IL-12 monotherapy, all p < 0.001), and markedly reduced the expression levels of the immunosuppressive factors TGF-β and the tumor cell surface PD-L1 (Figure 7-(b)).

### IL-12 combined with Osimertinib inhibits drug-resistant signaling pathways in tumor tissues

**Figure 8:**
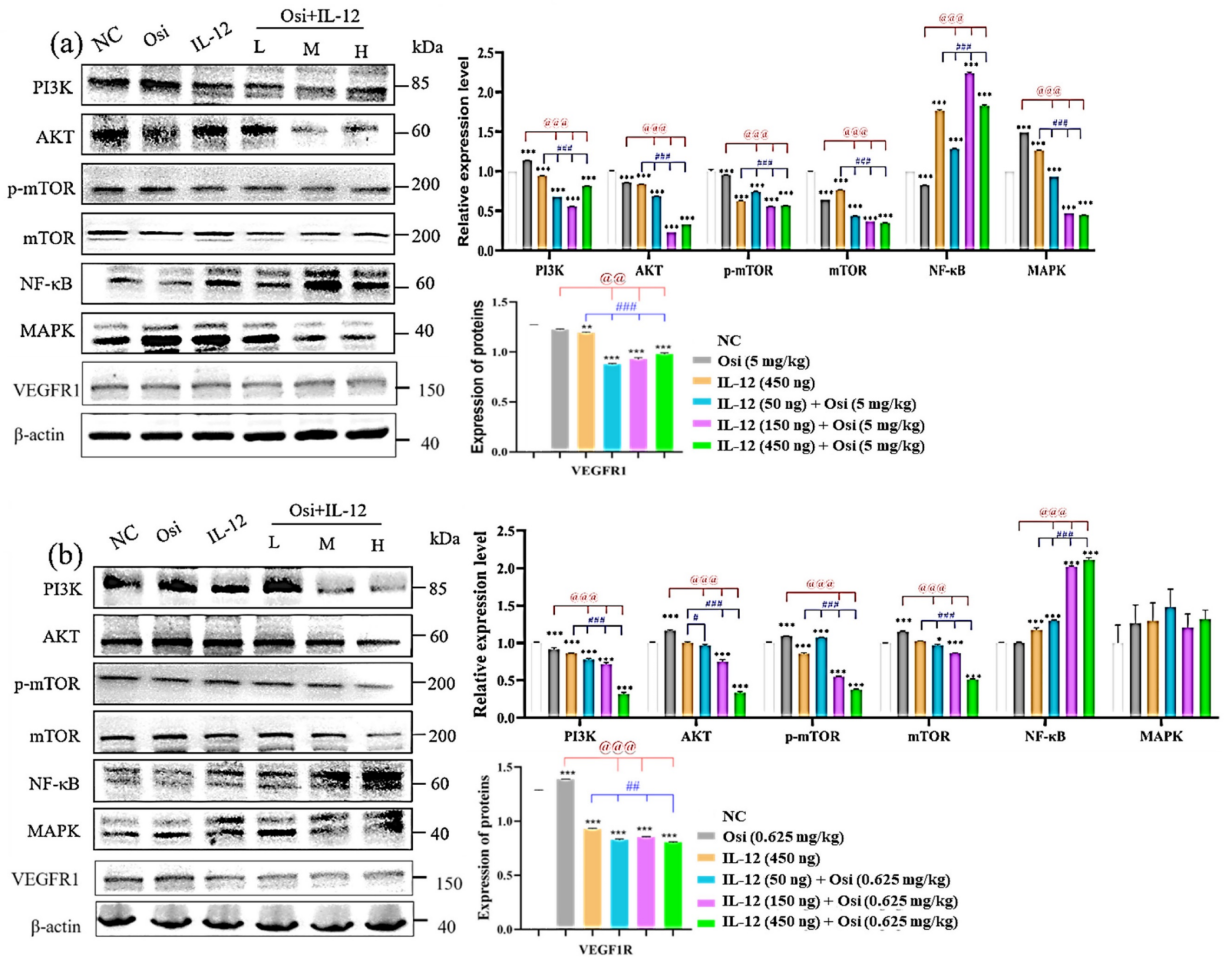
** Changes in the expression levels of key proteins in signaling pathways in tumor tissues of tumor-bearing mice.** (a) represent the H1975 (L858R/T790M/C797S) tumor model; (b) present the results in the H1975 (L858R/T790M) tumor model. Note: NC, solvent control group; Osi, Osimertinib group; L, combination low-dose group; M, combination medium-dose group; H, combination high-dose group; **, ***: Compared with the solvent control group, P<0.01, P<0.001; ##, ###: Compared with the IL-12 group, P<0.01, P<0.001; @@, @@@: Compared with the Osimertinib monotherapy group, P<0.01, P<0.001.

In Osimertinib-resistant NSCLC, western blot results in Figure 8-(a) reveal that the combination of IL-12 with Osimertinib significantly reduces the expression levels of p-mTOR, mTOR, PI3K, AKT, and MAPK in tumor tissues, while increasing NF-κB expression. The combination of IL-12 with Osimertinib may exert a reversal effect on Osimertinib resistance by inhibiting the PI3K/AKT/mTOR and RAF/MEK/MAPK signaling pathways.

Similarly, in Osimertinib-sensitive non-small cell lung cancer (Figure 8-(b)), the combination of IL-12 with Osimertinib significantly reduces the expression levels of p-mTOR, mTOR, PI3K, and AKT in tumor tissues, while increasing NF-κB expression. This suggests that the combination of IL-12 with Osimertinib may enhance efficacy by inhibiting the PI3K/AKT/mTOR signaling pathway.

### IL-12 combined with Osimertinib inhibits the expression of angiogenesis-related receptors in tumor tissues

Using the Western blot method to analyze the expression level of VEGFR1 (Figure 8), high levels of VEGFR1 expression were detected in both Osimertinib-sensitive and resistant H1975 tumor tissues. However, the application of IL-12 alone reduced the expression of VEGFR1 (compared to the NC group, P<0.01). The combination of low, medium, and high doses showed a significant inhibitory effect on VEGFR1 (compared to the NC group, P<0.001).

### IL-12 and Osimertinib Synergize to Modulate Apoptotic and Proliferative Responses in NSCLC Models

**Figure 9:**
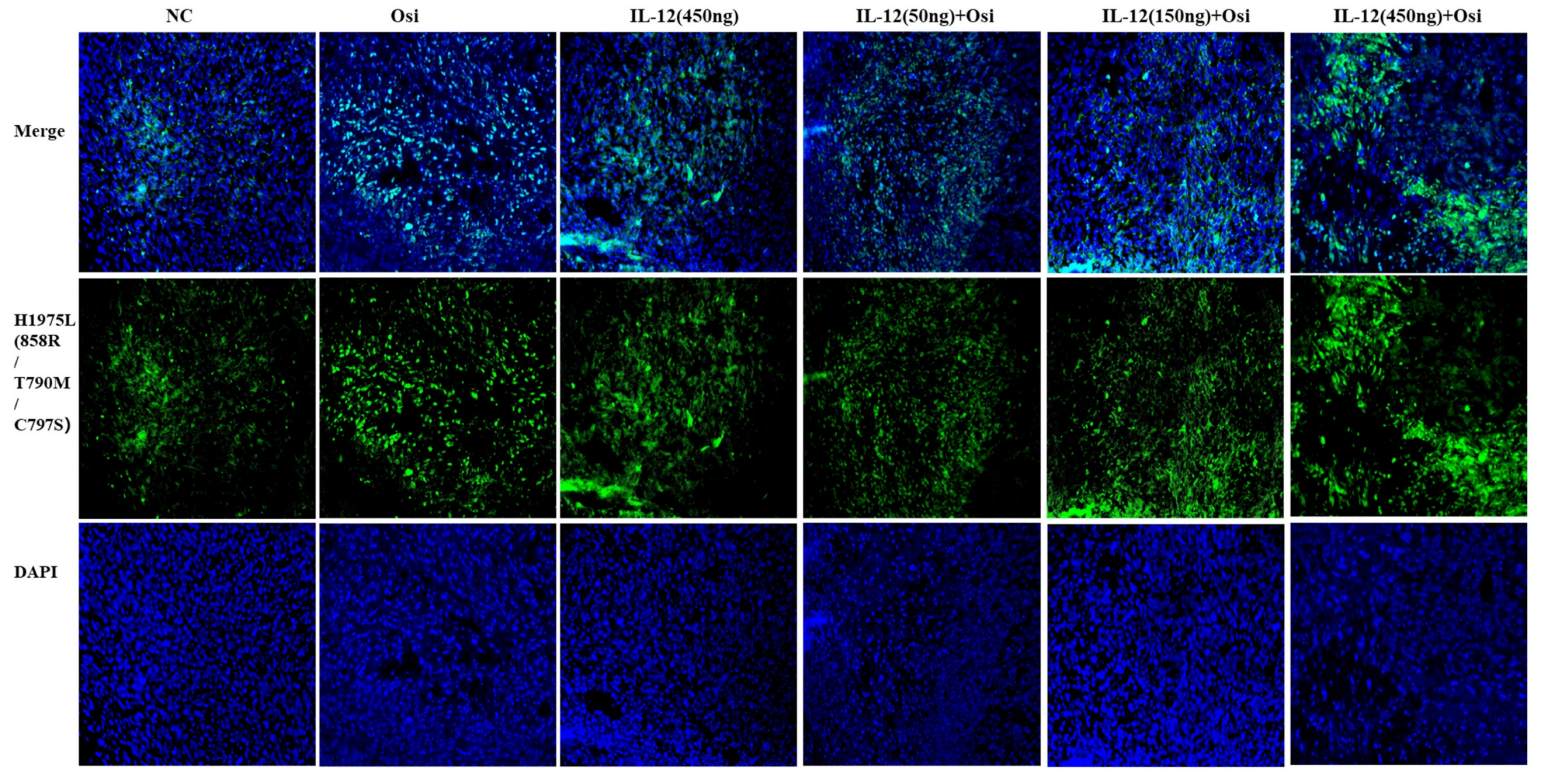

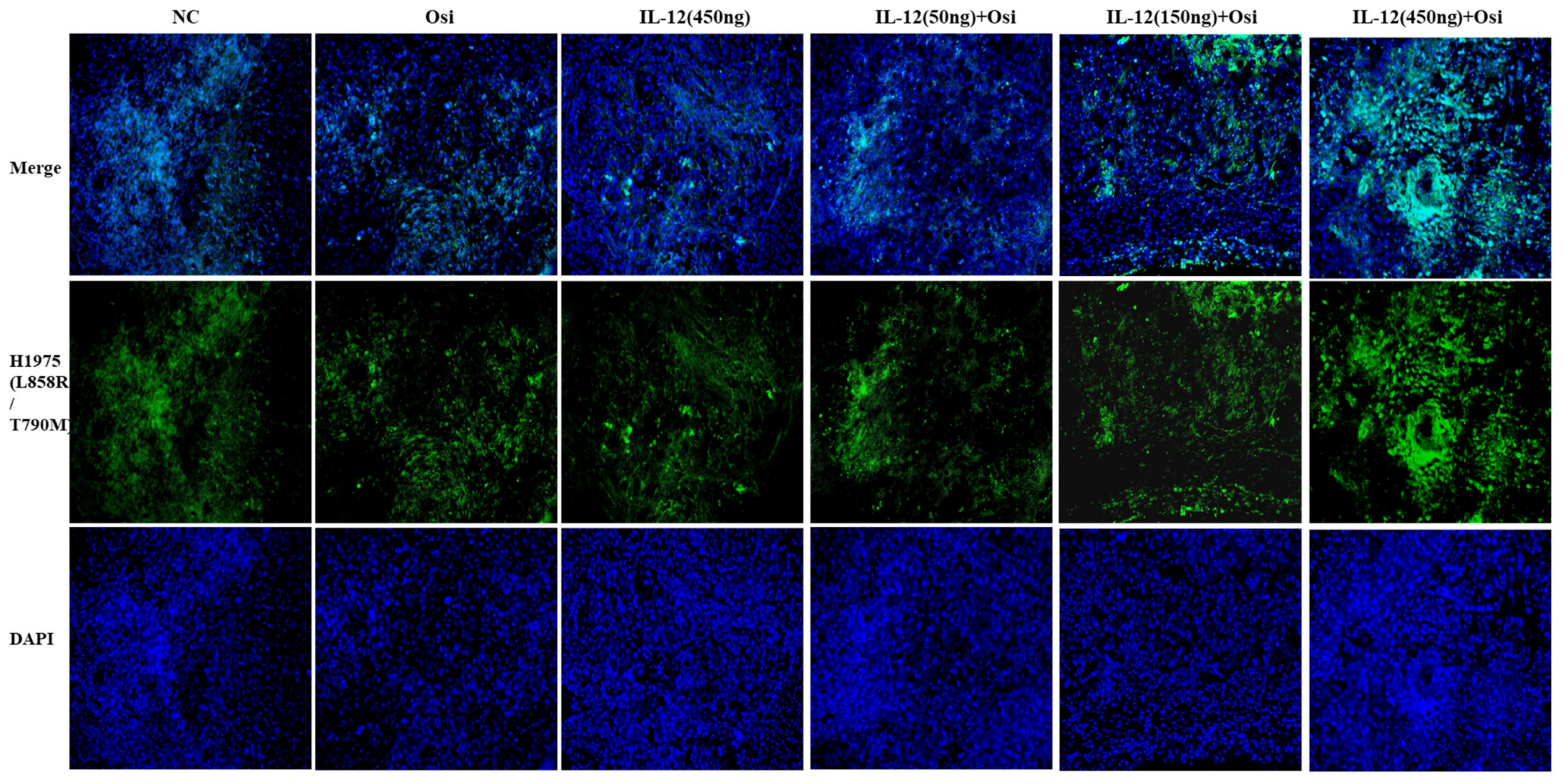

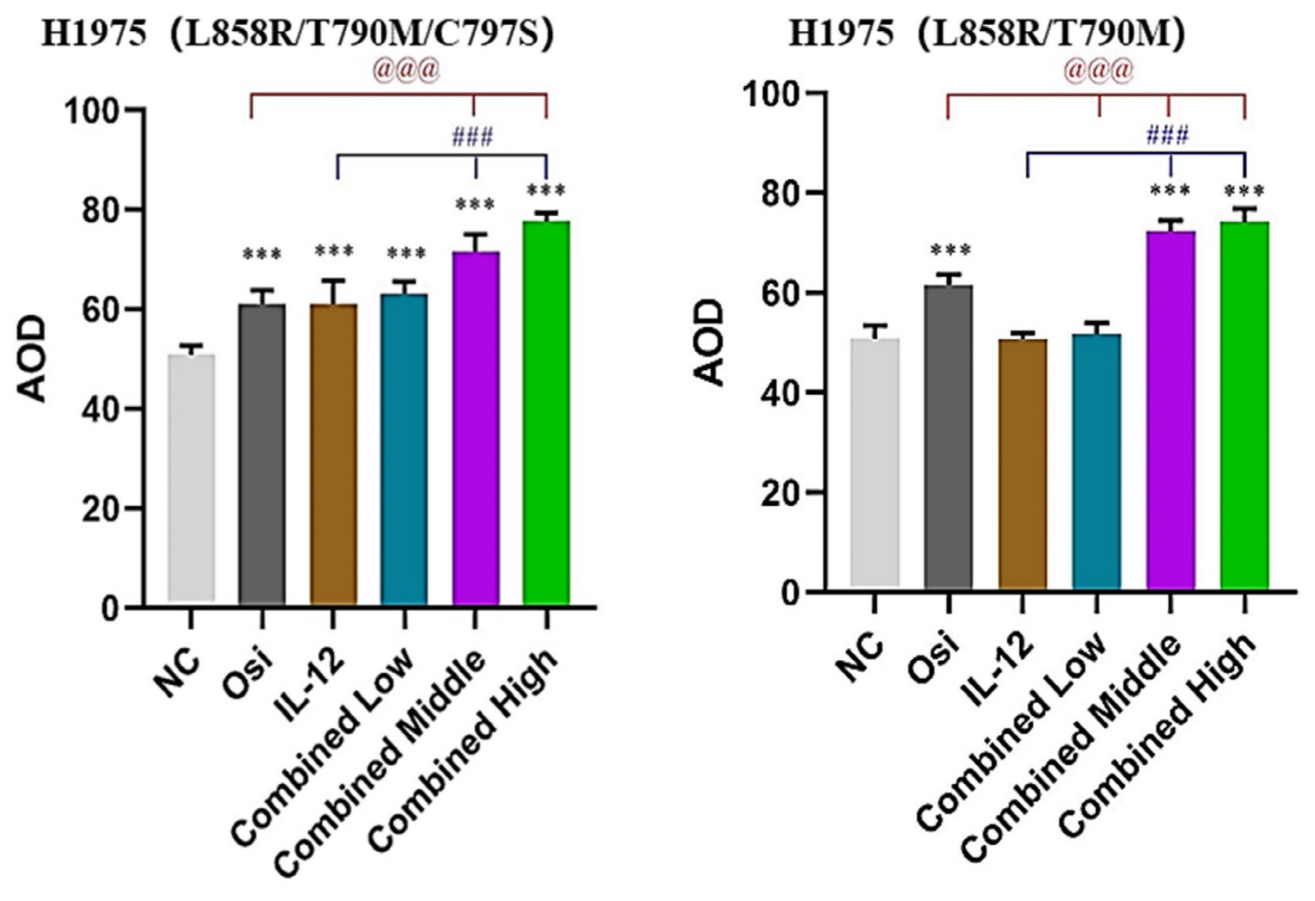
** TUNEL FITC green fluorescence and DAPI staining of NSCLC tumor tissues.** Top panels show the overlay of TUNEL staining (indicating apoptosis) and DAPI (indicating all nuclei) across different treatment groups. Middle panels present the TUNEL staining alone, while bottom panels display DAPI staining alone. Quantitative analysis of apoptotic cell density, represented as Average Optical Density (AOD), is summarized in the accompanying graphs to the right. The graphs depict a comparison of apoptosis levels in both osimertinib-resistant and sensitive H1975 cell models treated with NC (negative control), Osi (Osimertinib alone), IL-12 alone, and various combined dosages of IL-12 and Osimertinib. ***: Compared with the solvent control group, P<0.001; ###: Compared with the IL-12 group, P<0.001; @@@: Compared with the Osimertinib monotherapy group, P <0.001.

**Figure 10:**
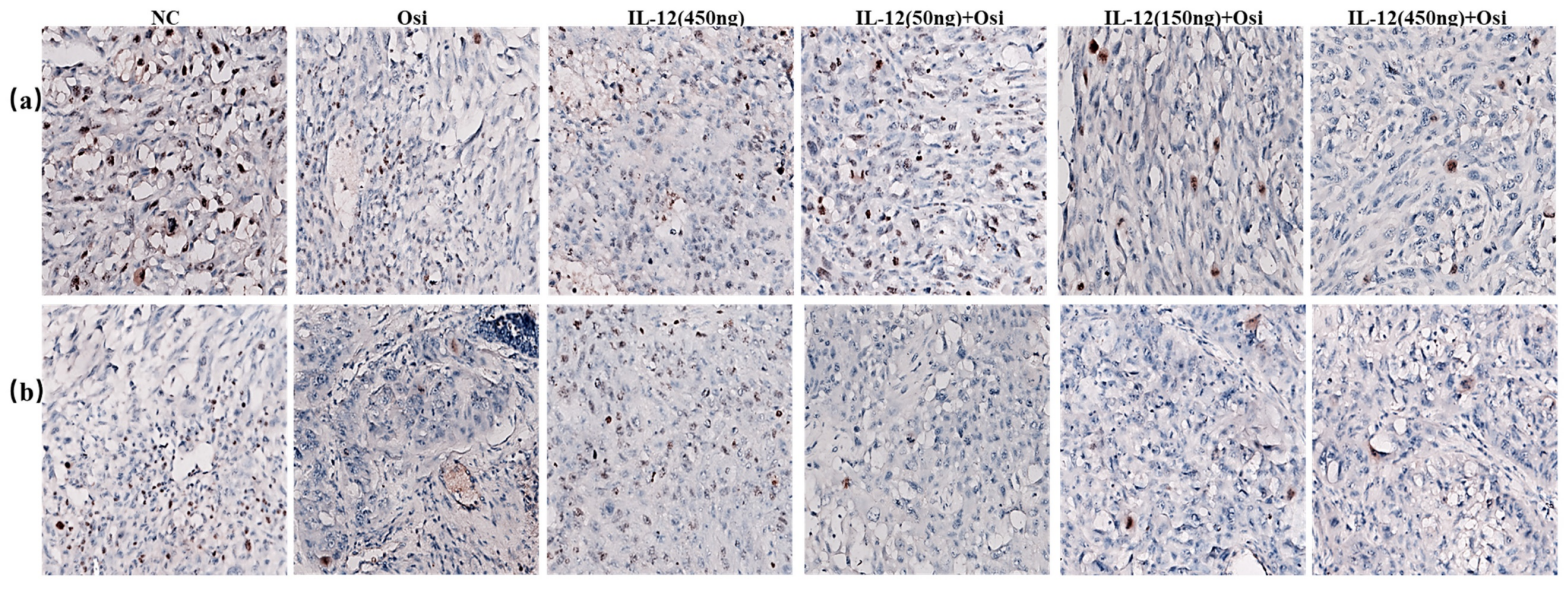

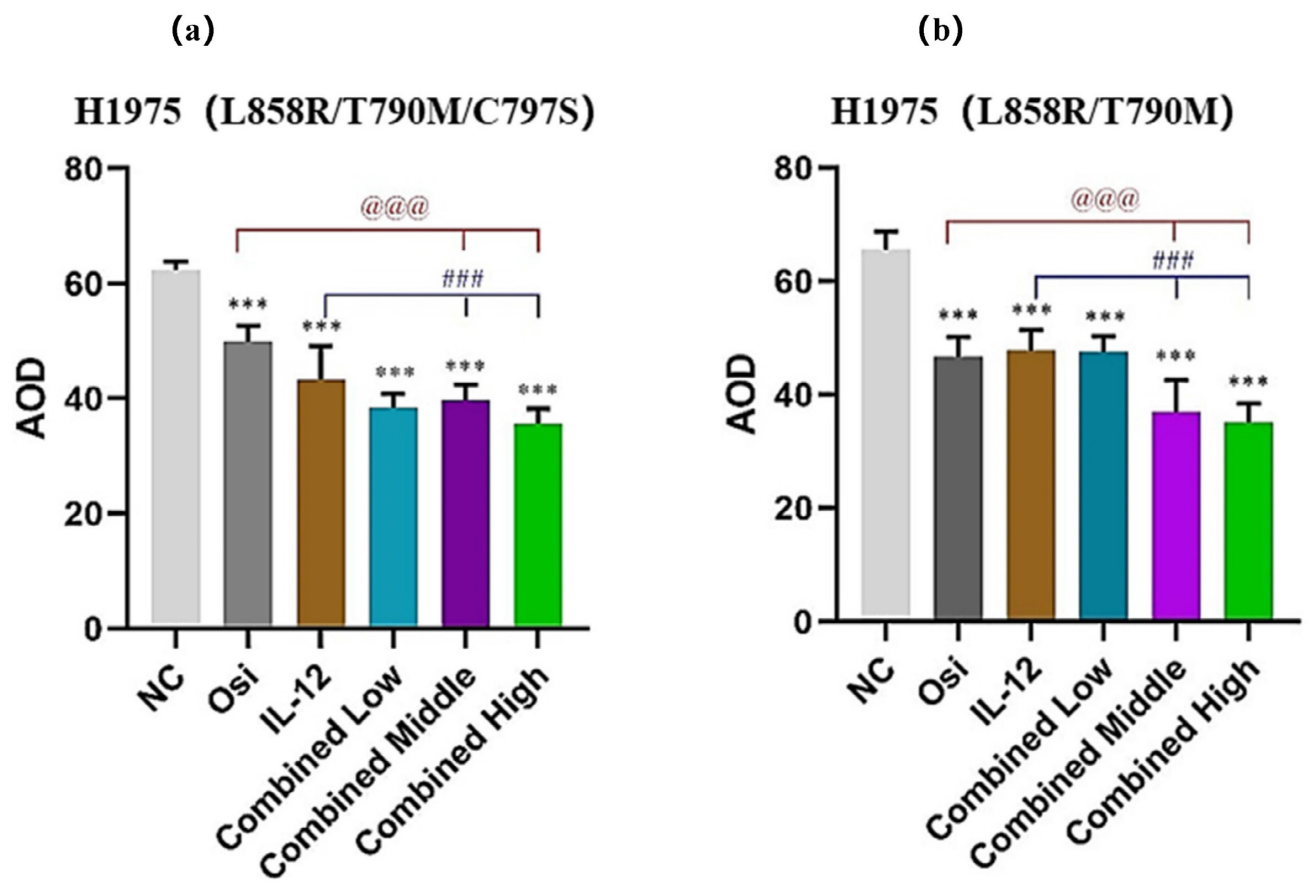
** Immunohistochemical staining for the proliferative marker Ki67 in tumor tissues from resistant and sensitive H1975 NSCLC cell line models.** Significant reductions in Ki67 positive cells, indicating decreased proliferation, were observed in combination therapy groups compared to monotherapy and control groups. The panels display the Ki67 staining across various treatment groups, with the corresponding quantitative analysis shown in the bar graphs. ***: Compared with the solvent control group, P<0.001; ###: Compared with the IL-12 group, P<0.001; @@@: Compared with the Osimertinib monotherapy group, P <0.001.

To thoroughly analyze and validate the impact of IL-12 combined with Osimertinib on the growth status of tumors, I conducted TUNEL and Ki67 assays to assess the effects of the combination on cell proliferation and apoptosis within tumor tissues. The results, depicted in Figures 9 and 10, represent a critical evaluation of the therapeutic impact on tumor cell dynamics.

From the TUNEL assay results, I observed a significant elevation in apoptotic cell counts in both the Osimertinib-resistant and sensitive H1975 cell lines when treated with the combination therapy, as compared to the monotherapies. This increment in apoptosis was particularly pronounced in the higher dosage groups, suggesting a dose-dependent enhancement of the pro-apoptotic effects due to the combination treatment.

Concurrently, Ki67 staining revealed a notable reduction in proliferation markers across both cell lines when subjected to the combined treatment. The reduction in Ki67 positive cells was in line with the observed increase in apoptosis, providing evidence of the comprehensive antitumor activity exerted by the combination of IL-12 and Osimertinib.

Collectively, these findings demonstrate that the combination of IL-12 and Osimertinib contributes to a robust antitumor response, characterized by diminished proliferation and escalated apoptosis rates. This synergistic interaction suggests an improvement in therapeutic outcomes for NSCLC patients, presenting a compelling case for integrating IL-12 with Osimertinib into clinical practice for combating tumor growth and resistance. These insights mark a significant advancement in understanding the multifaceted impact of immunomodulatory agents in synergy with targeted therapies for NSCLC management.

## Discussion

IL-12, a pivotal cytokine, plays a significant role in inducing cellular immunity with the ability to directly or indirectly eliminate tumors 14. By enhancing the differentiation of CD4+T cells into Th1 cells, promoting the development and proliferation of Th1 cells, IL-12 strengthens the effector functions of cellular immunity. Additionally, IL-12 facilitates the differentiation and proliferation of T cells and NK cells, enhances the cytotoxic activity of macrophages, and stimulates CTL and NK cells to release cytotoxic factors such as IFN-γ, crucial in the process of tumor clearance 15.

In our systematic study using the non-small cell lung cancer (NSCLC) H1975 model, comprising both Osimertinib-resistant and sensitive cell lines, we employed various analytical methods to investigate immune cell infiltration, immune factors, effector molecules, and resistance signaling pathway proteins. This aimed to elucidate the mechanisms of IL-12 in enhancing and reversing Osimertinib resistance.

**Figure 11:**
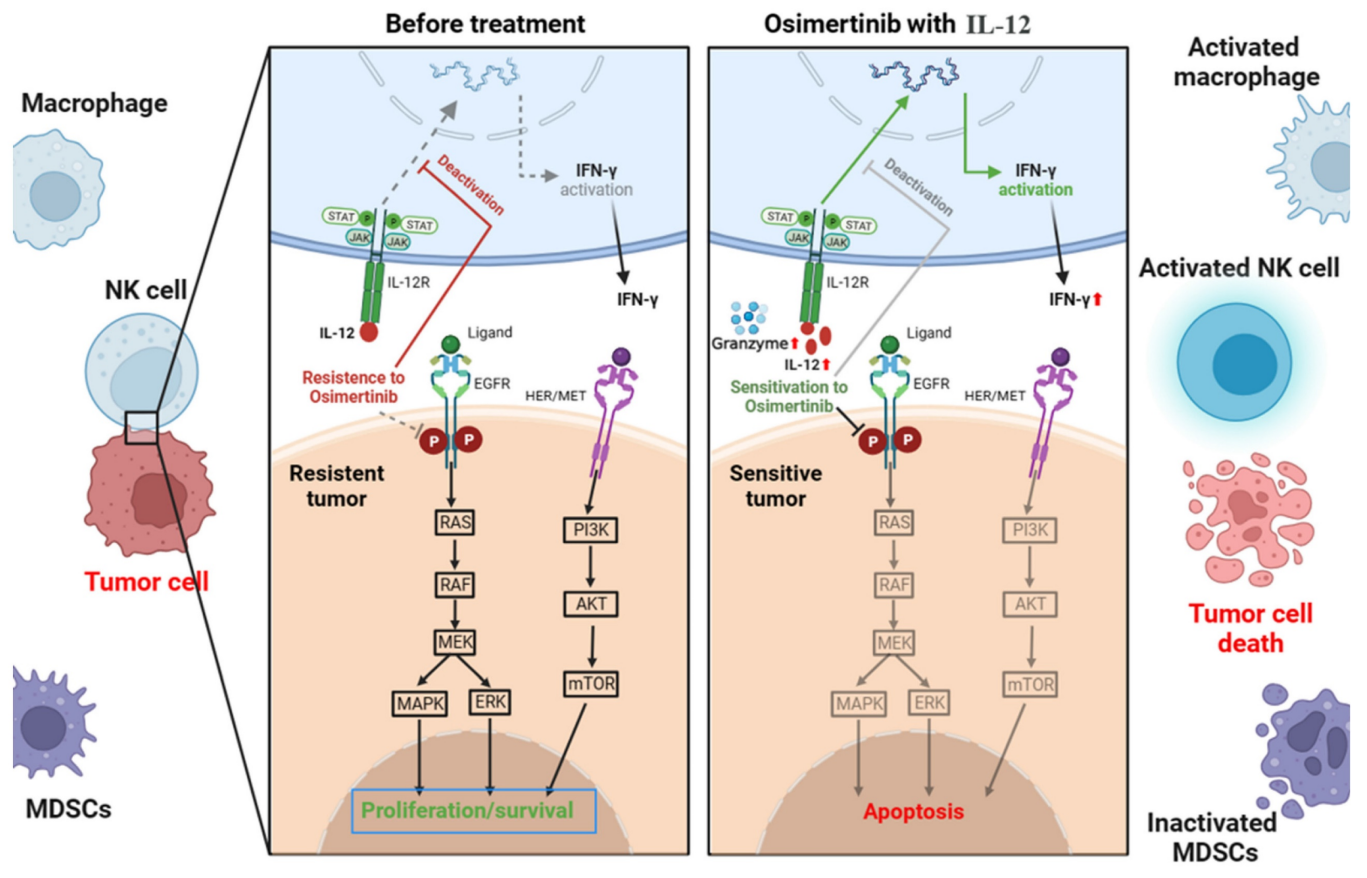
Mechanism of action of IL-12 in combination with Osimertinib in suppressing non-small cell lung cancer.

EGFR mutations induce changes in the immune microenvironment, resulting in a "non-inflammatory" tumor microenvironment with low immune cell infiltration, leading to resistance to Osimertinib. As depicted in Figure 11, IL-12 effectively increases immune cell infiltration, reduces immunosuppressive MDSCs and their subpopulations in tumor tissues, promotes the release of more cytotoxic factors (Granzyme B) and tumor-killing factor IFN-γ by immune cells, decreases the expression of PD-L1 on the surface of tumor cells, thereby improving the tumor microenvironment, restoring immune surveillance, and enhancing the sensitivity of cancer cells to Osimertinib.

The PI3K/protein kinase B (AKT)/mammalian target of rapamycin (mTOR) pathway, a crucial regulator of the cell cycle and proliferation, experiences dysregulation in 4-11% of patients progressing to osimertinib^14^, ^16^. Predominantly, genetic alterations manifest in PIK3CA (E454K, E542K, R88Q, N345K, and E418K), encoding the p100α protein, the catalytic subunit of PI3K 17, 18. Another frequent alteration found across various cancers is PTEN deletion, leading to heightened PI3K signaling and potentially contributing to osimertinib resistance 18. Our experiments demonstrate that in resistant tissues, there is an overexpression of p-mTOR, mTOR, PI3K, and AKT, aligning with the drug resistance mechanisms outlined in the literature. The co-administration of IL-12 with osimertinib significantly reduces the expression of these signaling molecules. IL-12 likely achieves this by activating immune responses, inhibiting the PI3K signaling pathway, thereby diminishing cellular proliferation and survival. Consequently, this reverses the observed drug resistance phenomenon.

The RAT sarcoma virus (RAS)/Raf murine sarcoma viral oncogene homolog B (RAF) pathway, also known as the mitogen-activated protein kinase (MAPK) pathway, plays a critical role in cell growth, division, and differentiation 19. Various genetic alterations within the MAPK pathway contribute to resistance against osimertinib^20^. Our experiments indicate that in osimertinib-resistant tissues, there is an increase in the expression of MAPK, consistent with the drug resistance mechanisms outlined in the literature. Importantly, the co-administration of IL-12 with osimertinib effectively reduces MAPK expression, potentially achieved by directly inhibiting the RAF/MEK/MAPK signaling pathway. This reduction in MAPK expression slows down cell proliferation and contributes to the successful reversal of osimertinib resistance.

Epithelial-mesenchymal transition (EMT), a process in which epithelial cells undergo loss of polarity and adhesion capacities, acquiring a mesenchymal phenotype that enables cellular migration, has been identified as a mechanism contributing to osimertinib resistance 21. a process in which epithelial cells undergo loss of polarity and adhesion capacities, acquiring a mesenchymal phenotype that enables cellular migration, has been identified as a mechanism contributing to osimertinib resistance 21. In our experiments, the co-administration of IL-12 with osimertinib increased the expression of NF-κB. This increase may potentially inhibit the EMT process, slowing down the invasion and migration of tumor cells. IL-12, by enhancing NF-κB expression, likely acts on two fronts: suppressing the NF-κB-mediated EMT process and promoting heightened sensitivity of cells to osimertinib. In combination, these effects contribute to the reversal of drug resistance.

The combined treatment of IL-12 with osimertinib exerts multifaceted regulatory effects on the drug resistance mechanisms in non-small cell lung cancer (NSCLC). IL-12 intervenes directly in cellular proliferation and survival by inhibiting the PI3K/AKT/mTOR and RAF/MEK/MAPK signaling pathways. Simultaneously, IL-12 may indirectly influence cellular transformation and migration processes by modulating the NF-κB pathway, thereby effecting the reversal of osimertinib resistance. Furthermore, the co-administration of IL-12 with osimertinib may enhance efficacy and counteract resistance by inhibiting the PI3K/AKT/mTOR and RAF/MEK/MAPK pathways associated with drug resistance. Additionally, the observed inhibition of VEGFR1 by IL-12 combined with Osimertinib suggests that the combination therapy may achieve its antitumor effects by suppressing vascular growth-related receptors in tumors.

In an effort to further substantiate the specificity and potential universal applicability of the synergistic effects observed with IL-12 and Osimertinib in overcoming EGFR TKI resistance, we incorporated the BAF3 (EGFR L858R/T790M) cell line model into our study (Supplementary Figure 1). This model, distinct from our primary NSCLC focus, allowed us to explore the combination therapy's efficacy in a different cellular context. While Osimertinib alone significantly inhibited tumor growth in the BAF3 model, indicating its robust activity against EGFR mutant-driven proliferation, the addition of IL-12 did not enhance this antitumor effect. The tumor inhibition rates for the combination therapy across varying doses did not demonstrate a synergistic interaction, which stands in contrast to the pronounced efficacy observed in the NSCLC models.

This comparative analysis underscores the complex nature of tumor microenvironments and their potential influence on therapeutic outcomes. The differential responses between BAF3 and H1975 models highlight the necessity of considering cellular and microenvironmental contexts in the development and application of combination therapies. The findings from the BAF3 model serve to delineate the boundaries of IL-12's synergistic potential with Osimertinib, reinforcing the importance of targeted therapeutic strategies that are tailored to specific tumor types and their unique resistance mechanisms.

In conclusion, despite the limitations of IL-12 monotherapy due to safety concerns 22, the therapeutic benefits of IL-12 combined with Osimertinib demonstrate significant advantages in enhancing efficacy and reducing potential toxicity and resistance risks. This combination strategy provides new possibilities for overcoming limitations of single treatment approaches, offering robust support for further optimization of NSCLC treatment strategies and improving patient survival rates. However, the translation of these findings from murine models to clinical applications requires more in-depth research and experimental validation.

## Supplementary Material

Supplementary appendices, data, and figure.

## Figures and Tables

**Table 1:** Tumor Weight and Tumor Inhibition Rate in Different Experimental Groups of OSi-Resistant NSCLC Models.

Group (n=8)	rmIL-12	Tumor Weight (g, mean±SD)	IRTW (%)	Spleen Index
Negative Control	—	1.13±0.15	—	1.61±0.20
Osimertinib	—	0.89±0.04	21.99	1.16±0.07
rmIL-12	450 ng	0.47±0.04**	58.97	1.50±0.19
Combined Low	50 ng	0.55±0.04*#	51.57	1.09±0.09
Combined Middle	150 ng	0.50±0.03**#	55.99	1.55±0.26
Combined High	450 ng	0.43±0.04**#	61.86	1.31±0.08

Note: Osimertinib was administered by oral gavage at a dose of 5 mg/kg/day, five times a week, for three weeks. rmIL-12 was administered by intraperitoneal and subcutaneous injection twice a week for three weeks. *, **: Compared to the NC group, P < 0.05, P < 0.01; #: Compared to the osimertinib group, P < 0.05.

**Table 2:** Tumor Weight and Tumor Inhibition Rate in Different Experimental Groups of Osi-Sensitive NSCLC Models.

Group	Drug & Administration	Dose	Tumor Weight (g, mean±SD)	IRTW (%)	Spleen Index
Negative Control	SubQ: 2x/week Gavage: 5x/week	——	1.04±0.17	—	1.70±0.19
rmIL-12 Low	rmIL-12 SubQ: 2x/week	50 ng	0.95±0.06	9.49	1.64±0.14
rmIL-12 Middle		150 ng	0.85±0.12	18.96	1.47±0.11
IL-12 High		450 ng	0.78±0.22	25.66	1.47±0.11
Osimertinib Low	Osimertinib Gavage: 5x/week	0.625 mg/kg	0.83±0.18	20.71	1.34±0.10
Osimertinib High		1.25 mg/kg	0.62±0.18	40.82	1.37±0.16
Combined Low	Osimertinib 0.625 mg/kg Gavage: 5x/week + rmIL-12 SubQ: 2x/week	IL-12 50 ng	0.56±0.10	46.76	1.40±0.14
Combined Middle		IL-12 150 ng	0.49±0.05*	52.63*	1.24±0.10
Combined High		IL-12 450 ng	0.47±0.09*	55.17*	1.19±0.12

Note: *: Compared to the NC group, P < 0.05.
