# Supplementary material for: Synergistic Anti-Tumor Efficacy Achieved by Reversing Drug Resistance through the Regulation of the Tumor Immune Microenvironment with IL-12 and Osimertinib Combination Therapy
Source: J Cancer. 2024 Jun 17;15(14):4534–50. doi: 10.7150/jca.95407 (PMC11242341; doi:10.7150/jca.95407)
Supplement: Supplementary file 1 — Supplementary appendices, data, and figure. [file jcav15p4534s1.pdf]

Statistical Data Appendix on the Synergistic Effects of IL-12 in the Treatment of Osimertinib-Resistant Non-Small Cell Lung Cancer H1975 (L858R/T790M/C797S)

Appendix 1 ： Individual Body Weight Data of Mice on D1 (g)

No.	NC （Control）	Osimertinib	IL-12	Combined Low Dose	Combined Med Dose	Combined High Dose
1	20.00	20.60	18.70	22.30	20.00	19.30
2	18.60	19.50	23.10	20.80	20.00	20.70
3	19.50	19.60	20.70	21.70	18.70	22.10
4	20.20	18.40	19.10	18.10	18.00	20.30
5	19.00	19.30	18.70	21.40	19.60	19.40
6	20.00	19.10	20.20	20.80	20.10	22.80
7	21.10	18.50	18.50	19.50	20.00	18.60
8	21.40	18.80	19.10	18.90	19.00	18.40
Mean	19.98	19.23	19.76	20.44	19.43	20.20
SD	0.96	0.71	1.56	1.46	0.78	1.60

Appendix 2 ： Individual Tumor Volume Data of Mice on D1 （mm^3）

No.	NC （Control）	Osimertinib	IL-12	Combined Low Dose	Combined Med Dose	Combined High Dose
1	94.61	111.93	104.82	82.45	72.70	128.46
2	102.00	115.14	144.53	70.38	86.66	118.34
3	60.52	101.35	86.41	132.44	119.36	87.68
4	101.68	136.91	141.85	115.55	72.33	78.52
5	121.28	117.05	90.85	162.26	157.59	159.36
6	96.73	105.02	92.37	89.99	146.16	152.92
7	84.06	76.68	79.19	98.59	71.46	66.30
8	156.81	87.87	103.65	98.36	96.28	84.72
Mean	102.21	106.50	105.46	106.25	102.82	109.54
SD	8.86	5.86	7.83	9.37	10.87	11.16

Appendix 3 ： Individual Body Weight Data of Mice on D4 (g)

No.	NC （Control）	Osimertinib	IL-12	Combined Low Dose	Combined Med Dose	Combined High Dose
1	20.00	20.30	18.10	20.00	19.30	23.50
2	20.00	19.90	20.00	21.10	20.00	20.20
3	21.10	19.10	20.58	20.80	18.50	18.60
4	18.40	19.50	18.70	17.10	17.50	19.30
5	20.00	18.70	19.40	21.50	20.00	19.70

6	21.10	18.80	20.00	20.60	20.00	22.80
7	20.70	19.00	17.70	19.20	21.00	19.10
8	20.00	20.40	19.90	19.70	18.60	18.30
Mean	20.16	19.46	19.30	20.00	19.36	20.19
SD	0.87	0.67	1.02	1.39	1.11	1.93

Appendix 4 ： ndividual Tumor Volume Data of Mice on D4 （mm^3）

No.	NC （Control）	Osimertinib	IL-12	Combined Low Dose	Combined Med Dose	Combined High Dose
1	129.27	231.85	121.59	121.01	105.78	237.66
2	122.96	253.84	157.26	140.28	129.01	210.25
3	138.50	180.53	143.17	188.02	206.10	190.15
4	209.01	150.28	230.00	166.86	139.71	151.57
5	175.24	132.06	147.14	224.28	246.43	224.88
6	130.12	156.54	216.00	180.55	223.59	155.26
7	127.50	110.73	89.63	114.59	116.25	82.82
8	222.58	104.63	212.78	194.61	179.33	105.06
Mean	156.90	165.06	164.70	166.28	168.27	169.70
SD	12.65	17.14	15.82	12.09	16.77	17.72

Appendix 5 ： Individual Body Weight Data of Mice on D8 (g)

No.	NC （Control）	Osimertinib	IL-12	Combined Low Dose	Combined Med Dose	Combined High Dose
1	20.00	20.50	18.50	19.80	19.00	22.20
2	19.60	20.00	21.40	20.60	19.30	20.00
3	21.70	19.80	20.00	19.60	18.30	18.30
4	18.70	18.90	19.50	17.10	17.20	18.80
5	19.47	18.40	18.50	21.10	19.80	19.60
6	21.00	20.00	20.00	20.20	17.80	23.70
7	20.80	18.30	17.10	18.30	20.30	18.60
8	20.00	18.90	20.00	18.10	18.20	18.40
Mean	20.16	19.35	19.38	19.35	18.74	19.95
SD	0.96	0.83	1.31	1.38	1.05	1.98

Appendix 6 ： Individual Tumor Volume Data of Mice on D8 （mm^3）

No.	NC （Control）	Osimertinib	IL-12	Combined Low Dose	Combined Med Dose	Combined High Dose
1	345.69	286.73	150.57	188.63	163.22	300.49
2	155.66	532.80	343.54	341.20	285.88	355.94
3	312.72	405.04	406.19	433.98	266.15	394.64

4	691.51	417.91	297.49	260.61	230.32	361.47
5	335.04	258.70	186.12	475.01	461.83	384.49
6	256.50	202.46	505.63	213.81	449.88	228.20
7	449.75	177.21	294.65	217.56	249.68	123.04
8	574.38	468.49	221.23	162.33	309.49	221.38
Mean	390.16	343.67	300.68	286.64	302.05	296.21
SD	55.05	41.19	37.22	37.03	32.98	30.60

Appendix 7 : Individual Body Weight Data of Mice on D11 (g)

No.	NC （Control）	Osimertinib	IL-12	Combined Low Dose	Combined Med Dose	Combined High Dose
1	21.70	22.30	19.20	20.00	19.20	21.50
2	19.90	20.00	22.00	20.80	20.00	20.00
3	23.00	20.00	19.90	20.00	18.40	18.40
4	19.80	19.00	18.90	17.80	17.20	18.90
5	19.50	20.00	18.80	21.20	19.50	19.40
6	18.00	20.90	20.00	19.20	18.20	24.60
7	20.90	18.80	16.40	18.60	20.20	18.70
8	20.00	19.50	20.90	18.80	18.40	18.80
Mean	20.35	20.06	19.51	19.55	18.89	20.04
SD	1.51	1.12	1.65	1.16	1.02	2.09

Appendix 8 : Individual Tumor Volume Data of Mice on D11 （mm^3）

No.	NC （Control）	Osimertinib	IL-12	Combined Low Dose	Combined Med Dose	Combined High Dose
1	642.12	698.61	295.10	298.81	328.50	590.93
2	386.83	636.01	412.65	399.74	395.15	328.77
3	788.50	611.55	531.90	707.88	379.96	873.04
4	1204.22	1049.62	656.05	703.79	501.81	786.72
5	637.82	387.19	432.34	650.71	986.70	601.23
6	444.34	440.20	615.24	479.65	567.88	470.40
7	1214.18	295.83	391.02	419.05	518.18	238.71
8	954.62	874.75	435.37	362.33	769.32	540.03
Mean	784.08	624.22	471.21	502.75	555.94	553.73
SD	100.38	79.95	38.22	51.25	70.12	67.28

Appendix 9 : Individual Body Weight Data of Mice on D15 (g)

No.	NC （Control）	Osimertinib	IL-12	Combined Low Dose	Combined Med Dose	Combined High Dose
1	22.00	21.90	19.00	20.20	19.80	21.10
2	21.00	19.50	21.70	20.90	19.30	20.00

3	23.70	20.00	19.80	20.30	18.20	18.60
4	20.10	19.40	19.30	18.70	17.80	18.70
5	19.70	20.00	20.00	20.90	19.40	18.70
6	21.30	20.70	21.10	20.40	18.30	24.80
7	21.30	18.90	16.30	18.40	20.00	15.30
8	20.80	19.50	21.70	18.80	18.90	18.00
Mean	21.24	19.99	19.86	19.83	18.96	19.40
SD	1.23	0.94	1.78	1.03	0.80	2.75

Appendix 10 ： Individual Tumor Volume Data of Mice on D15 （mm^3）

No.	NC （Control）	Osimertinib	IL-12	Combined Low Dose	Combined Med Dose	Combined High Dose
1	1008.35	1384.38	241.48	420.35	281.17	475.44
2	714.14	894.72	599.22	527.13	365.66	310.64
3	1168.00	856.15	643.43	668.73	361.07	703.99
4	1751.45	1113.42	543.39	798.71	503.83	655.48
5	1170.15	693.94	598.19	776.41	850.44	844.08
6	778.37	864.38	753.33	471.18	539.51	458.42
7	1329.11	686.03	466.22	322.37	687.16	289.87
8	1687.41	1191.38	513.66	317.35	430.98	547.03
Mean	1200.87	960.55	544.86	537.78	502.48	535.62
SD	120.17	78.12	47.54	60.42	59.69	60.78

Appendix 11 ： Individual Body Weight Data of Mice on D18 (g)

No.	NC （Control）	Osimertinib	IL-12	Combined Low Dose	Combined Med Dose	Combined High Dose
1	22.30	22.60	20.00	21.30	20.20	21.70
2	21.60	20.70	23.80	22.40	20.40	20.20
3	22.60	21.10	20.00	21.20	19.10	19.50
4	21.40	20.60	20.00	17.30	18.30	19.20
5	20.40	20.10	20.00	22.20	20.00	19.60
6	22.50	22.30	21.60	22.20	19.30	25.30
7	22.20	20.10	15.60	21.40	20.80	21.10
8	21.40	20.60	22.00	18.80	19.60	17.10
Mean	21.80	21.01	20.38	20.85	19.71	20.46
SD	0.74	0.95	2.37	1.83	0.80	2.39

Appendix 12 ： Individual Tumor Volume Data of Mice on D18 （mm^3）

No.	NC （Control）	Osimertinib	IL-12	Combined Low Dose	Combined Med Dose	Combined High Dose
1	1358.61	1605.69	271.05	665.16	276.32	480.00
2	906.67	1211.40	598.50	685.07	412.62	431.99

3	1464.38	918.96	563.79	582.64	398.97	760.07
4	2129.88	1534.48	794.77	804.43	447.45	737.55
5	1260.77	1194.46	517.94	731.75	568.39	496.77
6	832.08	965.39	1056.62	687.24	590.82	396.91
7	1552.93	833.43	467.07	460.65	729.00	336.86
8	2127.52	1423.41	673.27	531.97	691.12	603.46
Mean	1454.11	1210.90	617.88	643.61	514.34	530.45
SD	153.68	92.12	73.99	35.33	49.41	49.23

Appendix 13 : Individual Body Weight Data of Mice on D21 (g)

No.	NC (Control)	Osimertinib	IL-12	Combined Low Dose	Combined Med Dose	Combined High Dose
1	22.30	22.10	20.30	21.50	20.60	20.90
2	20.70	20.30	24.00	22.70	20.50	20.50
3	23.40	20.50	20.40	21.10	19.30	19.50
4	20.60	20.80	21.30	23.70	18.60	18.80
5	20.80	20.70	20.50	23.00	20.00	19.40
6	22.00	21.20	22.00	20.00	19.40	25.50
7	22.00	20.30	15.70	14.90	20.60	21.00
8	21.80	20.40	22.40	18.70	20.00	15.00
Mean	21.70	20.79	20.83	20.70	19.88	20.08
SD	0.96	0.61	2.42	2.86	0.72	2.91

Appendix 14 : Individual Tumor Volume Data of Mice on D21 (mm^3)

No.	NC (Control)	Osimertinib	IL-12	Combined Low Dose	Combined Med Dose	Combined High Dose
1	2014.10	1871.06	406.64	820.18	539.85	640.78
2	1882.86	1518.33	609.12	1458.87	711.00	488.93
3	2394.06	1288.18	788.28	1050.36	375.21	1054.42
4	3321.77	2270.74	936.90	1508.73	775.70	962.47
5	1748.53	1753.99	566.26	729.78	909.58	764.15
6	1104.78	857.33	758.94	808.56	667.81	754.46
7	2095.25	1361.71	698.77	1234.71	802.20	260.22
8	3135.25	1776.94	1103.00	905.05	538.33	596.16
Mean	2212.08	1587.29	733.49	1064.53	664.96	690.20
SD	230.50	135.72	68.98	95.97	54.60	80.45

Appendix 17: Tumor Weight, Tumor Weight Inhibition Rate (IRTW%) Statistical Data

Groups	№1	№2	№3	№4	№5	№6	№7	№8	Mean	S.E	IRTW%
Control	1.0010	0.7500	1.0750	2.0858	0.7788	0.6406	1.1291	1.6150	1.1344	0.1547	—

IL-12	0.9065	0.7778	0.8044	1.1018	1.0026	0.7829	0.7640	0.9399	0.8850	0.0392	21.99
Osi	0.2649	0.4186	0.4190	0.6366	0.4760	0.5879	0.3570	0.5638	0.4655	0.0397	58.97**
Combined Low	0.4592	0.6967	0.5864	0.6764	0.4315	0.7073	0.3953	0.4424	0.5494	0.0416	51.57*#
Combined Med	0.3595	0.4611	0.4027	0.4219	0.5201	0.6333	0.6360	0.5593	0.4992	0.0332	55.99**#
Combined High	0.3797	0.3379	0.6413	0.5698	0.5492	0.3358	0.2275	0.4199	0.4326	0.0446	61.86**#

Note: *, **: Compared with the solvent control group, P<0.05, P<0.01;
#: Compared with the Osimertinib monotherapy group, P<0.05.

Statistical Data Appendix on the Synergistic Effects of IL-12 in the Treatment of Osimertinib-Sensitive Non-Small Cell Lung Cancer H1975 (L858R/T790M/C797S)

Appendix 1: Individual Body Weight Data of Mice on D1 (g)

No.	NC（Control）	IL-12-Low	IL-12-Med	IL-12-High	Osi-Low	Osi-Low	Combined Low	Combined Med	Combined High
1	17.50	22.00	20.00	20.70	20.00	22.10	22.20	22.90	21.70
2	21.00	20.00	24.00	20.00	20.00	22.30	21.00	20.70	20.00
3	21.30	21.10	21.20	20.00	20.00	22.00	20.90	20.00	22.20
4	22.80	22.00	21.70	21.40	22.90	20.60	19.10	19.20	21.20
5	21.20	20.00	21.60	21.30	21.70	20.00	20.80	22.30	20.60
6	20.00	19.00	21.00	21.40	21.50	21.30	19.10	20.00	19.90
7	21.00	19.40	20.90	20.10	20.00	21.50	22.40	21.80	19.80
8	20.70	19.00	20.00	24.20	21.60	21.10	20.00	22.30	21.30
Mean	20.69	20.31	21.30	21.14	20.96	21.36	20.69	21.15	20.84
SD	0.53	0.44	0.45	0.49	0.39	0.28	0.44	0.48	0.32

Appendix 2: Individual Tumor Volume Data of Mice on D1 (mm^3)

No.	NC（Control）	IL-12-Low	IL-12-Med	IL-12-High	Osi-Low	Osi-Low	Combined Low	Combined Med	Combined High
1	86.77	45.66	41.13	149.15	54.77	139.26	148.23	53.79	79.02
2	72.13	128.41	81.07	132.02	119.38	132.18	48.98	70.13	85.26
3	76.20	59.26	62.00	51.98	44.45	112.06	199.75	93.26	110.15
4	126.72	65.02	61.59	109.63	59.79	70.15	71.09	146.70	43.75
5	86.58	99.21	176.71	96.74	140.67	80.94	115.94	184.82	143.03
6	116.98	141.36	201.89	133.02	101.96	55.15	55.35	77.86	105.15
7	99.23	149.04	73.26	82.13	154.28	114.87	80.32	81.96	91.56
8	133.28	111.04	84.17	42.30	105.58	71.56	74.99	89.74	131.54

Mean	99.73	99.88	97.73	99.62	97.61	97.02	99.33	99.78	98.68
SD	8.25	13.94	20.67	13.75	14.46	11.14	18.41	15.45	11.06

Appendix 3: Individual Body Weight Data of Mice on D5 (g)

No.	NC（Control）	IL-12-Low	IL-12-Med	IL-12-High	Osi-Low	Osi-Low	Combined Low	Combined Med	Combined High
1	18.60	21.70	21.00	21.30	19.30	22.80	23.60	22.50	23.50
2	20.80	20.00	24.80	21.50	20.90	22.50	21.30	20.50	20.50
3	21.70	21.50	21.90	20.60	20.90	22.50	20.50	21.80	22.00
4	21.70	22.40	21.40	21.90	22.50	21.00	20.50	20.00	22.80
5	21.30	20.00	21.70	22.00	22.00	21.10	21.30	22.00	21.10
6	20.00	19.20	21.90	22.10	22.00	21.60	19.50	20.00	19.50
7	20.00	20.00	20.00	20.80	20.00	20.90	21.70	23.40	20.70
8	21.70	18.80	20.00	24.60	22.20	20.90	20.00	23.10	21.60
Mean	20.73	20.45	21.59	21.85	21.23	21.66	21.05	21.66	21.46
SD	0.40	0.45	0.53	0.44	0.41	0.29	0.45	0.48	0.46

Appendix 4: Individual Tumor Volume Data of Mice on D5 （mm^3）

No.	NC（Control）	IL-12-Low	IL-12-Med	IL-12-High	Osi-Low	Osi-Low	Combined Low	Combined Med	Combined High
1	113.49	90.02	76.73	277.34	126.54	209.34	158.05	152.34	58.85
2	98.77	246.85	128.22	185.55	129.49	218.64	70.44	76.85	67.61
3	199.85	97.18	82.87	55.76	82.47	126.51	246.96	102.97	184.80
4	208.51	85.27	65.42	301.07	100.45	169.89	96.30	176.70	49.84
5	105.31	147.24	150.53	192.24	201.60	140.54	212.33	154.77	203.90
6	291.04	233.93	368.00	281.85	238.61	147.39	110.97	82.25	137.09
7	262.80	86.98	75.46	93.16	299.64	117.94	119.26	89.54	38.24
8	247.59	196.02	213.04	128.00	200.13	224.22	68.19	106.09	223.48
Mean	190.92	147.94	145.03	189.37	172.37	169.31	135.31	117.69	120.48
SD	26.93	24.30	36.44	32.64	26.53	15.14	23.15	13.45	26.84

Appendix 5: Individual Body Weight Data of Mice on D7 (g)

No.	NC（Control）	IL-12-Low	IL-12-Med	IL-12-High	Osi-Low	Osi-Low	Combined Low	Combined Med	Combined High
1	19.30	22.00	21.10	21.70	19.10	23.20	23.80	22.10	23.30
2	20.80	21.00	25.70	22.00	20.70	18.90	21.80	21.30	20.70
3	22.00	22.10	22.60	21.80	21.10	22.60	20.00	21.70	21.80
4	21.50	22.10	20.50	22.00	22.10	21.70	21.10	20.70	23.10

5	21.60	21.00	21.80	21.50	21.90	21.50	21.40	22.10	21.60
6	20.00	20.00	22.20	22.60	22.10	22.20	20.00	20.10	20.00
7	18.00	20.50	20.00	21.10	20.00	22.00	21.60	23.50	21.10
8	22.30	19.50	21.00	25.00	23.20	21.20	20.60	23.30	22.00
Mean	20.69	21.03	21.86	22.21	21.28	21.66	21.29	21.85	21.70
SD	0.53	0.35	0.63	0.43	0.47	0.45	0.43	0.42	0.40

Appendix 6: Individual Tumor Volume Data of Mice on D7（mm^3）

No.	NC（Control）	IL-12-Low	IL-12-Med	IL-12-High	Osi-Low	Osi-Low	Combined Low	Combined Med	Combined High
1	115.13	156.97	71.47	390.56	99.06	356.63	237.42	97.08	141.49
2	344.88	402.10	242.33	188.31	150.08	265.10	58.11	140.72	84.09
3	229.73	226.03	148.60	62.50	109.65	150.81	207.54	145.45	301.68
4	519.96	152.92	162.43	594.22	88.28	194.28	121.35	179.30	37.58
5	330.60	283.55	231.18	292.35	270.00	95.17	186.62	123.41	289.89
6	330.51	346.68	482.00	434.03	242.19	156.83	112.68	111.20	203.78
7	337.97	365.12	189.84	58.00	413.06	132.24	124.02	136.49	41.94
8	362.47	433.90	228.94	164.01	322.75	210.54	72.66	144.72	260.40
Mean	321.41	295.91	219.60	273.00	211.88	195.20	140.05	134.80	170.11
SD	40.76	38.38	42.41	67.20	42.12	29.46	22.68	8.80	38.54

Appendix 7: Individual Body Weight Data of Mice on D11 (g)

No.	NC（Control）	IL-12-Low	IL-12-Med	IL-12-High	Osi-Low	Osi-Low	Combined Low	Combined Med	Combined High
1	20.00	21.80	22.20	22.30	19.30	23.60	23.50	21.50	23.00
2	20.00	21.40	26.00	21.50	20.00	17.20	22.30	21.80	20.80
3	23.00	21.40	23.10	21.80	22.20	22.70	21.20	21.90	21.30
4	18.90	21.40	20.50	21.20	22.20	21.60	20.00	20.00	23.80
5	22.00	21.30	22.00	21.80	22.20	21.00	22.00	22.00	22.30
6	20.00	20.00	23.20	23.50	23.20	22.30	20.00	20.00	19.90
7	17.10	20.00	20.90	21.60	21.40	22.60	20.60	23.00	21.70
8	22.80	20.00	21.50	25.10	23.40	21.60	20.00	22.60	22.50
Mean	20.48	20.91	22.43	22.35	21.74	21.58	21.20	21.60	21.91
SD	0.72	0.27	0.61	0.46	0.51	0.69	0.46	0.39	0.44

Appendix 8: Individual Tumor Volume Data of Mice on D11（mm^3）

No.	NC（Control）	IL-12-Low	IL-12-Med	IL-12-High	Osi-Low	Osi-Low	Combined Low	Combined Med	Combined High

1	206.62	533.43	142.33	745.76	338.48	1084.91	793.94	210.49	229.38
2	459.28	658.23	900.34	285.67	552.73	392.02	115.79	344.60	144.07
3	469.26	365.29	420.18	202.83	246.23	344.25	526.27	291.69	1170.61
4	1032.49	478.68	496.58	1223.86	241.40	458.40	162.91	386.22	98.54
5	510.48	435.55	647.47	358.48	596.57	262.27	579.46	188.94	547.16
6	458.64	793.27	1157.48	902.49	514.99	390.76	207.18	171.11	440.96
7	680.81	689.04	470.10	147.04	887.17	309.11	383.78	259.83	80.25
8	684.71	1369.73	403.79	546.76	836.42	260.53	159.85	330.45	465.43
Mean	562.79	665.40	579.78	551.61	526.75	437.78	366.15	272.92	397.05
SD	85.49	112.46	112.43	134.04	87.33	95.53	87.25	27.75	127.41

Appendix 9: Individual Body Weight Data of Mice on D14 (g)

No.	NC (Control)	IL-12-Low	IL-12-Med	IL-12-High	Osi-Low	Osi-Low	Combined Low	Combined Med	Combined High
1	20.40	21.40	21.80	22.20	19.30	23.50	23.60	21.80	22.50
2	20.50	21.40	25.20	21.80	18.70	16.70	22.00	22.30	20.00
3	23.50	22.30	23.10	22.10	21.90	23.40	20.00	21.30	21.10
4	17.80	21.90	20.60	21.40	22.30	20.20	20.00	20.00	23.50
5	22.70	21.90	22.10	22.20	21.60	21.20	22.00	22.20	22.10
6	21.40	20.30	24.70	23.40	24.00	22.80	20.30	20.50	20.00
7	15.70	20.00	20.90	21.60	22.40	22.20	20.20	23.60	21.20
8	23.00	20.00	22.00	25.60	23.60	20.90	20.70	22.10	22.80
Mean	20.63	21.15	22.55	22.54	21.73	21.36	21.10	21.73	21.65
SD	0.96	0.33	0.59	0.49	0.66	0.79	0.46	0.40	0.46

Appendix 10: Individual Tumor Volume Data of Mice on D14（mm^3）

No.	NC (Control)	IL-12-Low	IL-12-Med	IL-12-High	Osi-Low	Osi-Low	Combined Low	Combined Med	Combined High
1	336.41	843.93	158.28	963.74	365.39	1349.21	826.98	346.71	378.86
2	568.99	1556.44	1185.42	489.98	723.04	493.01	191.59	685.92	216.25
3	776.99	697.42	928.22	287.79	558.58	446.57	698.72	360.76	1012.44
4	1873.64	728.55	909.14	1681.83	366.94	486.65	257.75	638.67	216.03
5	761.91	912.55	920.79	701.49	809.05	265.13	632.73	200.34	572.91
6	847.05	987.37	1769.76	1264.46	743.13	408.56	289.67	301.43	520.99
7	905.97	895.83	825.46	208.90	1177.41	152.49	623.72	471.51	90.55
8	970.28	1178.68	660.76	761.64	1097.81	251.83	318.23	362.67	711.48

Mean	880.16	975.10	919.73	794.98	730.17	481.68	479.92	421.00	464.94
SD	158.90	98.64	160.86	176.22	106.58	131.44	85.27	59.11	107.46

Appendix 11: Individual Body Weight Data of Mice on D18 (g)

No.	NC (Control)	IL-12-Low	IL-12-Med	IL-12-High	Osi-Low	Osi-Low	Combined Low	Combined Med	Combined High
1	19.50	22.50	22.00	22.70	19.20	25.10	23.80	22.50	22.10
2	20.70	21.20	24.50	22.30	17.60	17.50	22.30	21.80	20.80
3	24.00	23.20	24.20	21.80	21.70	24.40	21.30	21.30	21.10
4	17.80	22.20	21.10	22.40	22.30	21.00	20.40	20.90	23.40
5	22.60	21.80	22.50	21.70	21.30	22.10	21.80	21.90	23.30
6	22.00	20.70	24.60	23.80	24.20	22.10	20.00	21.20	20.00
7	15.80	18.80	21.00	21.30	22.70	22.70	19.40	23.90	21.00
8	23.50	19.90	21.00	25.80	24.00	20.80	20.00	23.00	21.20
Mean	20.74	21.29	22.61	22.73	21.63	21.96	21.13	22.06	21.61
SD	1.02	0.51	0.57	0.51	0.80	0.83	0.52	0.36	0.43

Appendix 12: Individual Tumor Volume Data of Mice on D18 (mm^3)

No.	NC (Control)	IL-12-Low	IL-12-Med	IL-12-High	Osi-Low	Osi-Low	Combined Low	Combined Med	Combined High
1	545.51	1301.99	280.58	1804.53	503.46	2318.71	882.50	660.24	555.08
2	876.95	1633.40	1784.84	852.23	931.34	823.47	301.31	753.98	343.26
3	1306.10	1132.64	1522.22	674.36	549.21	904.38	902.42	572.46	1415.30
4	1905.26	984.59	1175.75	2101.41	500.35	924.87	560.49	824.50	282.18
5	1274.77	1738.97	1106.68	786.64	1465.14	543.77	1274.27	415.83	540.43
6	1320.22	1244.51	1994.62	1532.55	1213.93	402.84	495.70	567.74	451.89
7	1880.26	1267.36	1182.89	347.23	1657.71	496.83	716.90	664.30	146.21
8	1364.26	1414.02	685.74	713.33	1711.93	361.96	391.92	637.27	930.43
Mean	1309.17	1339.69	1216.66	1101.53	1066.63	847.10	690.69	637.04	583.10
SD	161.23	88.23	197.96	221.33	182.74	224.52	113.29	43.92	144.67
IRTV%	--	-2.3	7.1	15.9	18.5	35.3	47.2	51.3	55.5

Appendix 13: Tumor Weight, Tumor Weight Inhibition Rate (IRTW%) Statistical Data

Groups	№1	№2	№3	№4	№5	№6	№7	№8	Mean	S.E	IRTW%
Control	0.4975	0.5526	1.031	1.5951	0.8105	0.9486	1.8591	1.0594	1.0442	0.17	--
IL-12-Low	0.8938	0.8568	0.8359	0.8489	1.2726	0.8935	1.1224	0.837	0.9451	0.06	9.49

IL-12-Mid	0.2073	1.1859	0.7036	1.2087	0.9058	1.1503	0.8176	0.5902	0.8462	0.12	18.96
IL-12-High	0.7805	0.636	0.405	2.1744	0.5821	1.0857	0.1775	0.3685	0.7762	0.22	25.66
Osi-Low	0.2546	0.6336	0.3224	0.39	1.1056	0.9866	1.5062	1.4249	0.8280	0.18	20.71
Osi-High	1.8367	0.6006	0.6296	0.5996	0.4373	0.3872	0.212	0.2403	0.6179	0.18	40.82
Combined Low	0.6161	0.1944	0.7493	0.5373	1.1	0.402	0.5827	0.2656	0.5559	0.10	46.76
Combined Med	0.5125	0.5224	0.5018	0.7225	0.2432	0.423	0.523	0.509	0.4947	0.05	52.63*
Combined High	0.4	0.2935	0.753	0.2699	0.7078	0.3616	0.1124	0.8468	0.4681	0.09	55.17*

Note: *: Compared with the solvent control group, P<0.05.

Appendix 14: Organ Weight Statistical Data Table - Spleen

Groups	№1	№2	№3	№4	№5	№6	№7	№8	Mean	S.E
Control	0.205	0.3	0.3545	0.4512	0.3541	0.4015	0.3823	0.3013	0.3437	0.03
IL-12-Low	0.4485	0.3189	0.3097	0.2315	0.4908	0.3995	0.276	0.3112	0.3483	0.03
IL-12-Mid	0.2449	0.2798	0.3874	0.3113	0.3474	0.3841	0.4445	0.2599	0.3324	0.02
IL-12-High	0.3854	0.3052	0.3131	0.4074	0.3171	0.4474	0.25	0.2473	0.3341	0.03
Osi-Low	0.1674	0.2716	0.311	0.254	0.2922	0.439	0.2564	0.3365	0.2910	0.03
Osi-High	0.5075	0.312	0.4093	0.2768	0.2418	0.3128	0.2115	0.153	0.3031	0.04
Combined Low	0.3052	0.2593	0.4365	0.2602	0.4298	0.2501	0.1812	0.2596	0.2977	0.03
Combined Med	0.2639	0.3454	0.1772	0.2356	0.3108	0.3518	0.2498	0.251	0.2732	0.02
Combined High	0.1507	0.2845	0.2773	0.2304	0.317	0.226	0.1951	0.3749	0.2570	0.03

Immunohistochemistry and Immunofluorescence Statistical Data Table of Tumor Tissue in H1975 (L858R/T790M/C797S) Non-Small Cell Lung Cancer in Mice

Appendix 1 Immunohistochemistry Statistical Data Table of CD56+ NK Cells in Tumor Tissue of H1975 (L858R/T790M/C797S) Non-Small Cell Lung Cancer in Mice

No.	Control	Osi	IL-12	Combined Low	Combined Med	Combined High
1-View1	91.2	96.9	93.3	119.4	134.5	127.4
1-View2	120.3	111.5	107.5	99.5	127.0	124.9
1-View3	99.7	114.8	114.5	134.0	125.2	142.6
2-View1	111.0	119.8	115.5	115.6	135.6	145.1
2-View2	116.4	127.5	120.5	136.8	113.5	124.6
2-View3	108.9	104.5	110.0	119.6	112.1	121.4
3-View1	102.1	116.2	132.0	–	–	–
3-View2	99.1	116.0	115.6	–	–	–
3-View3	91.3	101.6	119.9	–	–	–
Mean	104.4	112.1	114.3	120.8	124.7	131.0

Appendix 2 Immunohistochemistry Statistical Data Table of CD68+ Macrophages in Tumor Tissue of H1975 (L858R/T790M/C797S) Non-Small Cell Lung Cancer in Mice

No.	Control	Osi	IL-12	Combined Low	Combined Med	Combined High
1-View1	104.9	104.0	120.6	125.4	136.0	135.1
1-View2	114.5	111.4	112.0	132.2	132.9	161.1
1-View3	92.5	125.0	133.5	121.8	138.0	131.3
2-View1	85.7	112.0	121.7	120.0	127.6	142.0
2-View2	93.8	124.9	131.6	128.3	129.8	170.3
2-View3	114.3	123.2	122.7	126.5	135.1	133.4
3-View1	88.2	113.3	84.2	–	–	–
3-View2	89.7	100.5	93.4	–	–	–
3-View3	90.9	103.5	96.6	–	–	–
Mean	97.2	113.1	112.9	125.7	133.2	145.5

Appendix 3 Immunohistochemistry Statistical Data Table of CD45+ White Blood Cells in Tumor Tissue of H1975 (L858R/T790M/C797S) Non-Small Cell Lung Cancer in Mice

No.	Control	Osi	IL-12	Combined Low	Combined Med	Combined High
1-View1	86.45	112.44	107.47	109.75	121.17	136.54
1-View2	89.25	119.27	108.11	117.36	118.21	141.10
1-View3	101.29	100.56	113.82	116.62	116.68	140.24
2-View1	100.33	87.14	134.17	113.93	135.81	119.10
2-View2	88.88	103.53	118.40	122.05	126.90	131.00
2-View3	85.14	94.72	96.98	131.18	121.14	115.68
3-View1	99.41	88.49	82.39	–	–	–
3-View2	73.90	116.66	122.94	–	–	–
3-View3	85.84	114.87	97.13	–	–	–
Mean	90.06	104.18	109.05	118.48	123.32	130.61

Appendix 4 Immunofluorescence Statistical Data Table of CD11b+ Myeloid Leukocytes in Tumor Tissue of H1975

No.	Control	Osi	IL-12	Combined Low	Combined Med	Combined High
1-View1	28.77	41.39	45.31	54.22	70.99	88.41
1-View2	33.59	42.19	62.18	74.54	71.38	97.60
1-View3	29.87	48.13	44.32	88.91	67.72	74.00
2-View1	40.64	43.00	58.20	69.42	61.38	75.71
2-View2	36.21	60.08	43.59	43.54	68.31	56.01
2-View3	38.17	43.39	52.31	67.12	74.28	62.43
3-View1	50.59	51.03	38.79	–	–	–
3-View2	48.34	62.74	44.69	–	–	–
3-View3	48.45	78.26	50.06	–	–	–
Mean	39.40	52.25	48.83	66.29	69.01	75.69

Appendix 5 Immunofluorescence Statistical Data Table of CD86+ M1-Type Macrophages in Tumor Tissue of H1975 (L858R/T790M/C797S) Non-Small Cell Lung Cancer in Mice

No.	Control	Osi	IL-12	Combined Low	Combined Med	Combined High
1-View1	34.8	65.9	60.6	37.2	50.4	65.6
1-View2	51.5	65.3	70.2	59.5	53.3	58.2
1-View3	49.0	42.3	41.5	69.0	86.0	49.9
2-View1	38.6	36.8	51.4	54.5	63.9	64.9
2-View2	46.6	63.8	52.4	53.8	54.7	64.6
2-View3	50.7	57.0	41.7	71.2	57.9	85.2
3-View1	53.7	46.9	66.7	–	–	–
3-View2	39.4	47.3	54.8	–	–	–
3-View3	41.0	53.5	41.0	–	–	–
Mean	45.0	53.2	53.4	57.5	61.0	64.7

Appendix 6 Immunofluorescence Statistical Data Table of CD206+ M2-Type Macrophages in Tumor Tissue of H1975 (L858R/T790M/C797S) Non-Small Cell Lung Cancer in Mice

No.	Control	Osi	IL-12	Combined Low	Combined Med	Combined High
1-View1	26.7	22.0	26.3	20.8	30.4	35.9
1-View2	29.7	26.8	20.6	22.2	34.9	28.4
1-View3	21.3	29.7	25.6	38.7	14.4	16.8
2-View1	18.6	30.4	30.0	22.4	29.7	27.5
2-View2	28.1	24.4	27.3	27.6	37.7	18.2
2-View3	27.5	29.8	36.1	45.6	21.2	32.4
3-View1	29.3	24.6	36.7	–	–	–
3-View2	25.1	48.4	31.3	–	–	–
3-View3	32.2	24.2	25.1	–	–	–
Mean	26.5	28.9	28.8	29.6	28.0	26.5

Appendix 7 Immunohistochemistry Statistical Data Table of CD56+ NK Cells in Tumor Tissue of H1975 (L858R/T790M) Non-Small Cell Lung Cancer in Mice

No.	Control	Osi	IL-12	Combined Low	Combined Med	Combined High
1-View1	102.1	113.4	103.2	118.6	84.2	106.9
1-View2	91.0	111.2	127.0	113.0	87.2	88.6
1-View3	99.0	115.8	119.8	104.6	104.2	87.2
2-View1	86.2	96.9	114.9	107.1	94.1	98.3
2-View2	116.2	97.9	95.9	127.6	95.9	105.0
2-View3	87.6	109.2	97.6	121.4	108.3	94.4
3-View1	95.5	98.7	102.3	–	–	–
3-View2	92.4	106.0	108.3	–	–	–
3-View3	90.0	116.3	84.1	–	–	–
Mean	95.5	107.3	105.9	115.4	95.6	96.7

Appendix 8 Immunohistochemistry Statistical Data Table of CD68+ Macrophages in Tumor Tissue of H1975 (L858R/T790M) Non-Small Cell Lung Cancer in Mice

No.	Control	Osi	IL-12	Combined Low	Combined Med	Combined High
1-View1	86.4	87.7	91.1	104.1	92.8	95.4
1-View2	97.6	92.8	97.4	132.1	116.3	81.8
1-View3	93.6	102.2	113.9	126.1	102.1	133.5
2-View1	93.0	124.1	119.8	106.4	127.0	102.5
2-View2	89.0	110.7	108.6	146.5	103.6	89.2
2-View3	78.2	107.9	100.2	128.6	100.6	110.3
3-View1	104.6	99.3	112.5	–	–	–
3-View2	99.2	100.1	84.2	–	–	–
3-View3	103.7	125.7	99.6	–	–	–
Mean	93.9	105.6	103.1	124.0	107.1	102.1

Appendix 9 Immunohistochemistry Statistical Data Table of CD45+ White Blood Cells in Tumor Tissue of H1975 (L858R/T790M) Non-Small Cell Lung Cancer in Mice

No.	Control	Osi	IL-12	Combined Low	Combined Med	Combined High
1-View1	78.8	102.6	107.0	117.0	118.3	104.4
1-View2	87.5	115.4	83.2	117.1	112.8	101.7
1-View3	93.0	116.8	99.7	111.1	95.3	82.3
2-View1	63.2	82.3	127.0	107.3	99.6	100.8
2-View2	87.6	124.7	90.6	87.8	93.2	102.6
2-View3	96.1	100.3	92.6	116.6	114.8	86.1
3-View1	108.2	95.0	122.7	–	–	–
3-View2	91.7	106.6	77.6	–	–	–
3-View3	84.1	94.3	113.3	–	–	–
Mean	87.8	104.2	101.5	109.5	105.7	96.3

Ki56 Tumor Cell Proliferation Analysis

Appendix 1 Mouse H1975 (L858R/T790M/C797S) Non-Small Cell Lung Cancer Tumor Tissue Ki56 Analysis Tumor Cell Proliferation Data

No.	Control	Osi	IL-12	Combined Low	Combined Med	Combined High
1-View1	60.5	48.3	41.7	40.1	38.5	36.4
1-View2	62.4	50.2	38.6	36.8	36.9	32.4
1-View3	63.9	54.3	35.4	39.4	40.4	33.8
2-View1	62.1	51	47	36.9	41.4	37.3
2-View2	61.8	52.2	39.2	36.8	36.6	39
2-View3	60.1	48.5	52.2	39.7	37.2	39
3-View1	63	46.8	43.3	38	41.7	35.8
3-View2	64.5	45.7	51.7	35	44.5	32.2
3-View3	63.1	52.2	40.6	43	41.2	35.4
Mean	62.37777778	49.91111111	43.3	38.41111111	39.82222222	35.7

Appendix 2 Mouse H1975 (L858R/T790M) Non-Small Cell Lung Cancer Tumor Tissue Ki56 Analysis Tumor Cell Proliferation Data

No.	Control	Osi	IL-12	Combined Low	Combined Med	Combined High
1-View1	66.4	41.9	42.9	45.5	35.8	30.2
1-View2	64.6	45.6	45.6	46.3	32.6	34.2
1-View3	60.3	47.8	48.9	49.8	33.7	29.9
2-View1	68.7	49	43.5	49.7	48	38.3
2-View2	63.9	48.4	54.1	44.7	42.4	38.8
2-View3	67.4	42.4	47.4	51.7	30.9	33.8
3-View1	69.8	52.9	46.9	47	38.2	36.2
3-View2	67.7	45.3	50.8	49.7	39.2	37.6
3-View3	62.1	47.5	50.2	44.3	32.6	37.3
Mean	65.65555556	46.75555556	47.81111111	47.63333333	37.04444444	35.14444444

Tumor Cell TUNEL Apoptosis Analysis

Appendix 1 Mouse H1975 (L858R/T790M/C797S) Non-Small Cell Lung Cancer Tumor Tissue TUNEL Apoptosis Analysis Data

No.	Control	Osi	IL-12	Combined Low	Combined Med	Combined High
1-View1	47.3	63.6	62.1	60.4	72.4	79.5
1-View2	53.1	56.8	65.9	63.9	75.1	76.4
1-View3	52.4	58.9	70.3	68.3	73.8	80.3
2-View1	49.4	65	61.2	63.8	67	75.9
2-View2	52.7	64.4	59.5	60	73.9	76.2
2-View3	50.9	60.5	58.2	62.7	73.6	76.3
3-View1	49.6	60.2	55.5	64.1	65.6	78
3-View2	50.2	60.7	59.9	62.3	73.9	79.1
3-View3	52	60.1	56.4	62.4	69.2	78
Mean	50.844444	61.13333333	61	63.1	71.611111	77.74444444

Appendix 2 Mouse H1975 (L858R/T790M) Non-Small Cell Lung Cancer Tumor Tissue TUNEL Apoptosis Analysis Data

No.	Control	Osi	IL-12	Combined Low	Combined Med	Combined High
1-View1	47.9	58.9	48.7	49.3	72.4	76.3
1-View2	50.2	62.3	50.4	53.1	69.3	70.2
1-View3	54.3	63.7	52.3	54.9	75.8	77.8
2-View1	46.1	60.9	49.7	49.8	73.5	74.3

2-View2	53.2	61.5	51.5	51.7	70.8	74.4
2-View3	52	64.7	50.2	53.3	73.6	75
3-View1	52.3	60.7	52.1	49.8	72.4	71.5
3-View2	50.3	59	50.6	49.6	73.8	77
3-View3	51.5	62.8	51.5	54.2	70.2	71.2
Mean	50.866667	61.61111111	50.77777778	51.74444444	72.422222	74.18888889

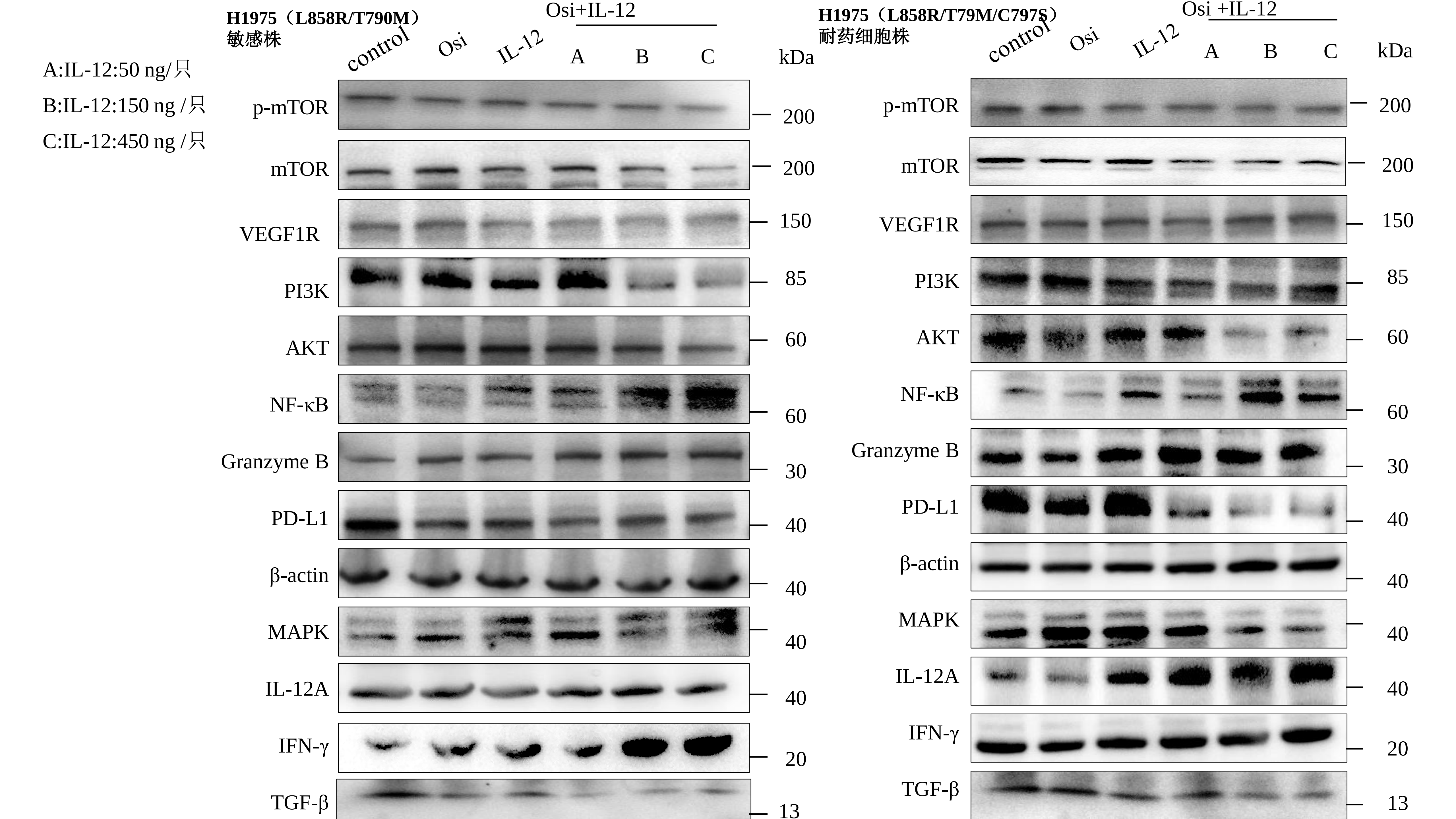
A:IL-12:50 ng/只
B:IL-12:150 ng /只
C:IL-12:450 ng /只
H1975（L858R/T790M）
敏感株
control
Osi
IL-12
Osi+IL-12
A
B
C
kDa
p-mTOR
200
mTOR
200
VEGF1R
150
PI3K
85
AKT
60
NF-κB
60
Granzyme B
30
PD-L1
40
β-actin
40
MAPK
40
IL-12A
40
IFN-γ
20
TGF-β
13
H1975（L858R/T79M/C797S）
耐药细胞株
control
Osi
IL-12
Osi +IL-12
A
B
C
kDa
p-mTOR
200
mTOR
200
VEGF1R
150
PI3K
85
AKT
60
NF-κB
60
Granzyme B
30
PD-L1
40
β-actin
40
MAPK
40
IL-12A
40
IFN-γ
20
TGF-β
13

A:IL-12:50 ng/只

B:IL-12:150 ng /只

C:IL-12:450 ng /只

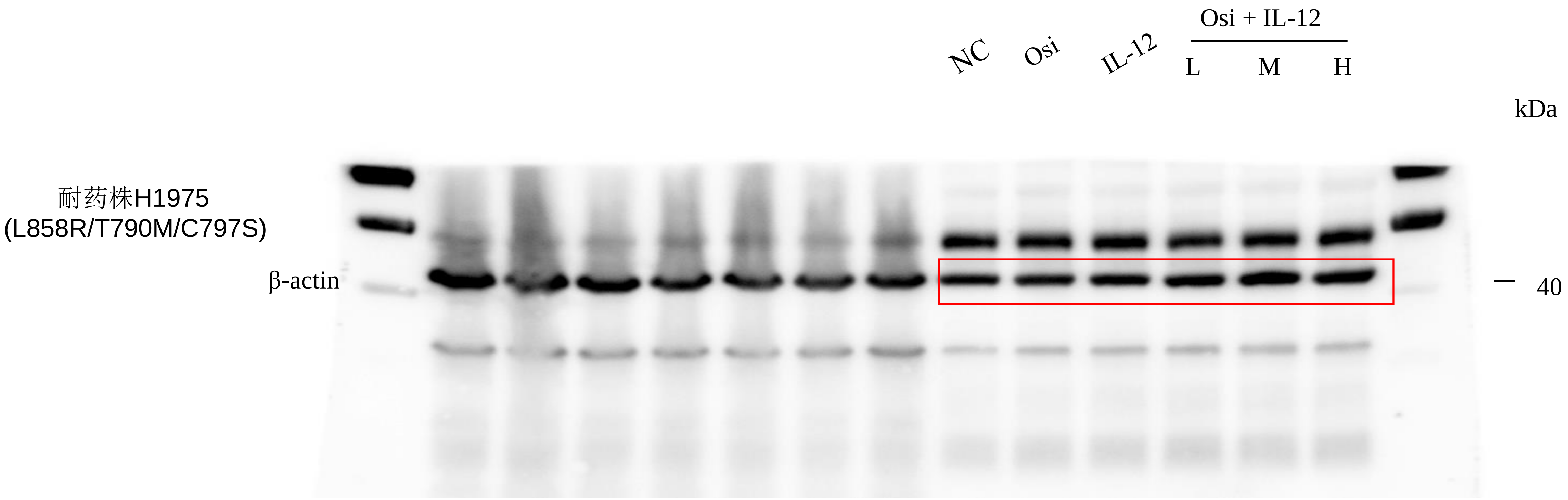

A:IL-12:50 ng/只

B:IL-12:150 ng /只

C:IL-12:450 ng /只

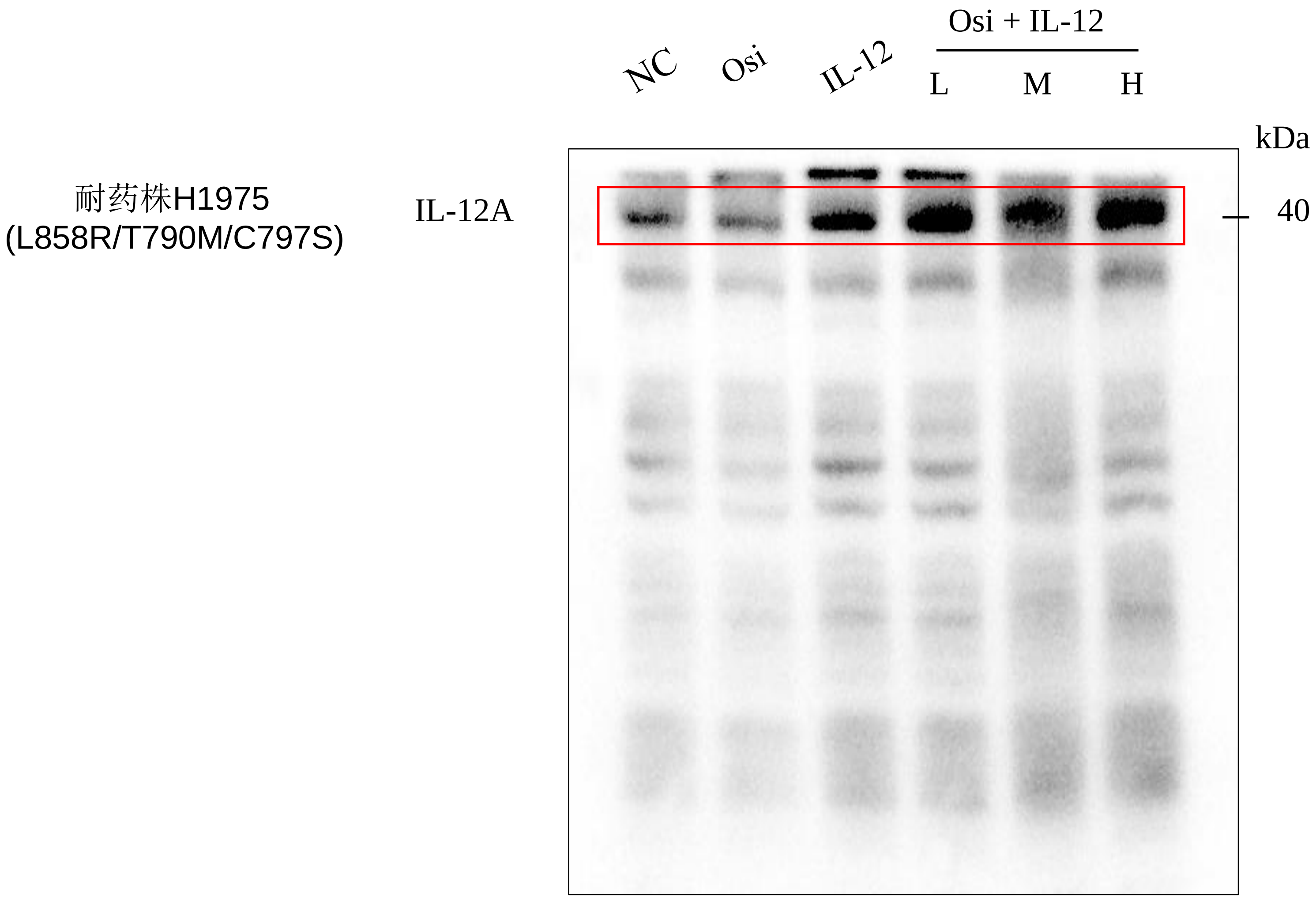

A:IL-12:50 ng/只

B:IL-12:150 ng /只

C:IL-12:450 ng /只

耐药株H1975
(L858R/T790M/C797S)

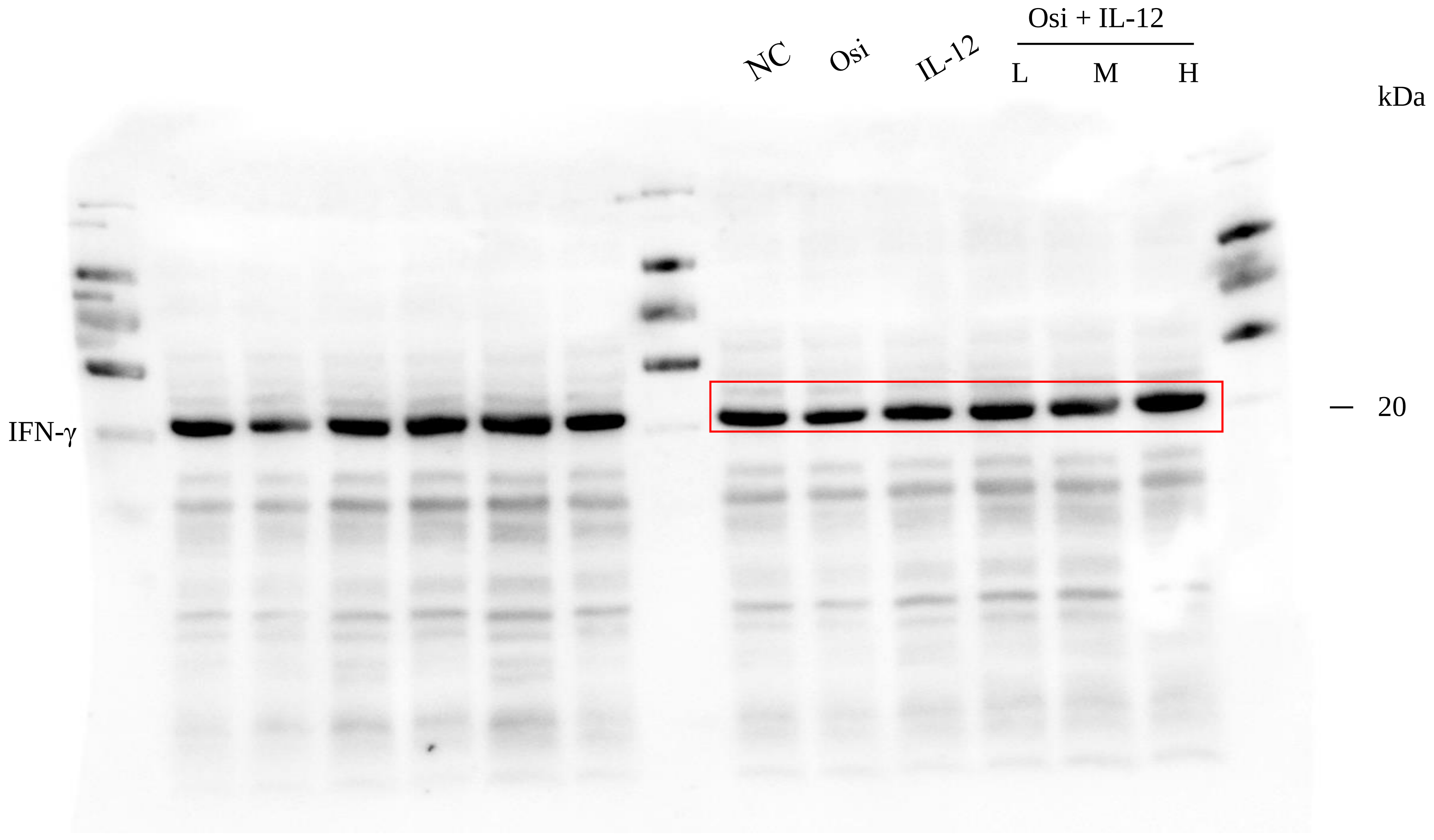

A:IL-12:50 ng/只

B:IL-12:150 ng /只

C:IL-12:450 ng /只

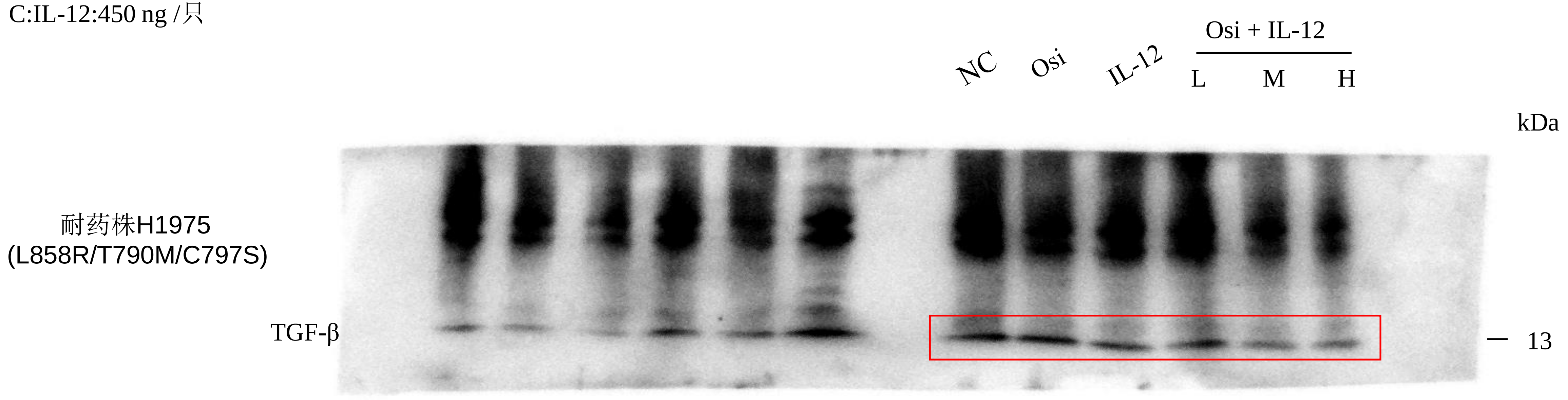

L:IL-12:50 ng/只

M:IL-12:150 ng /只

H:IL-12:450 ng /只

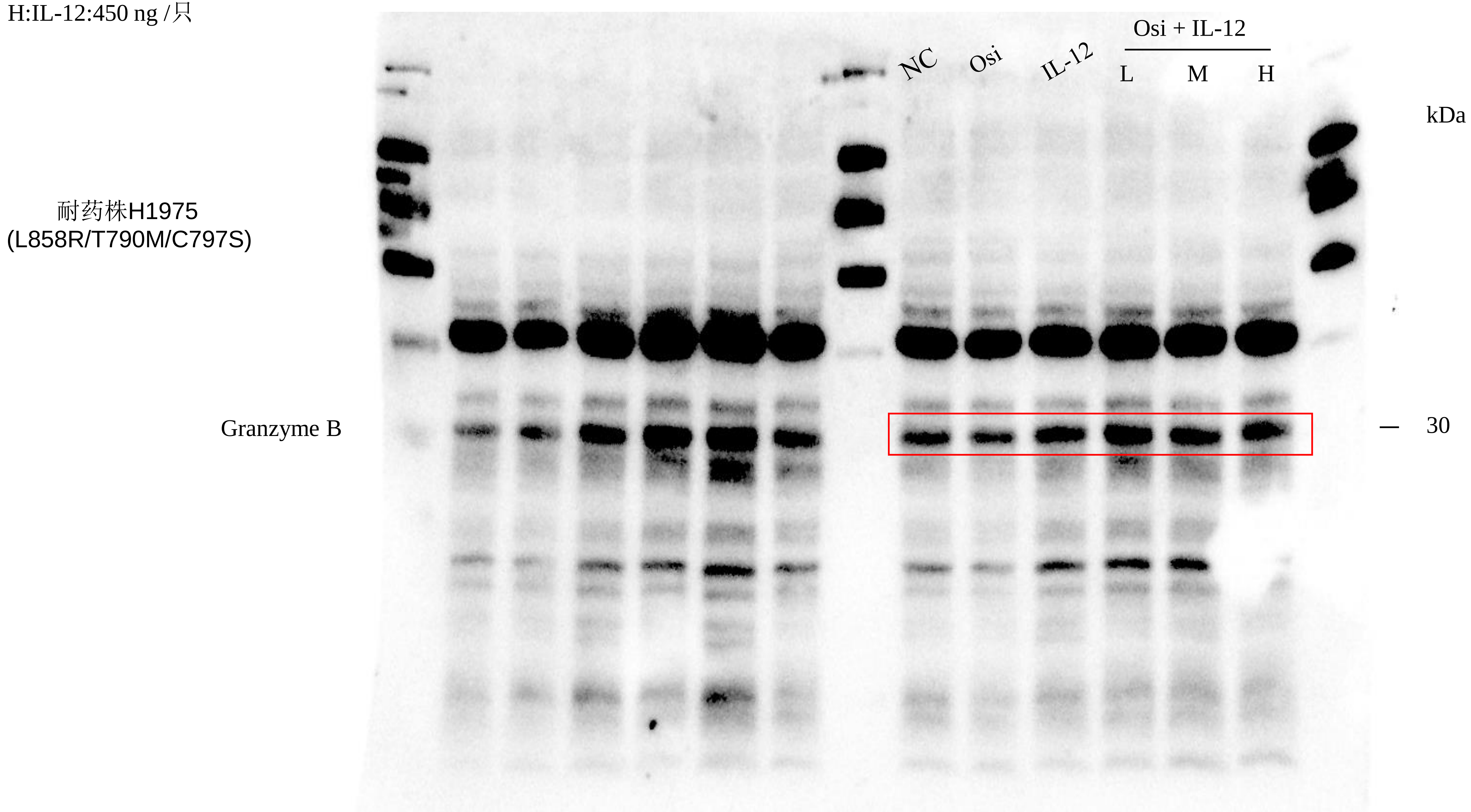

L:IL-12:50 ng/只

M:IL-12:150 ng /只

H:IL-12:450 ng /只

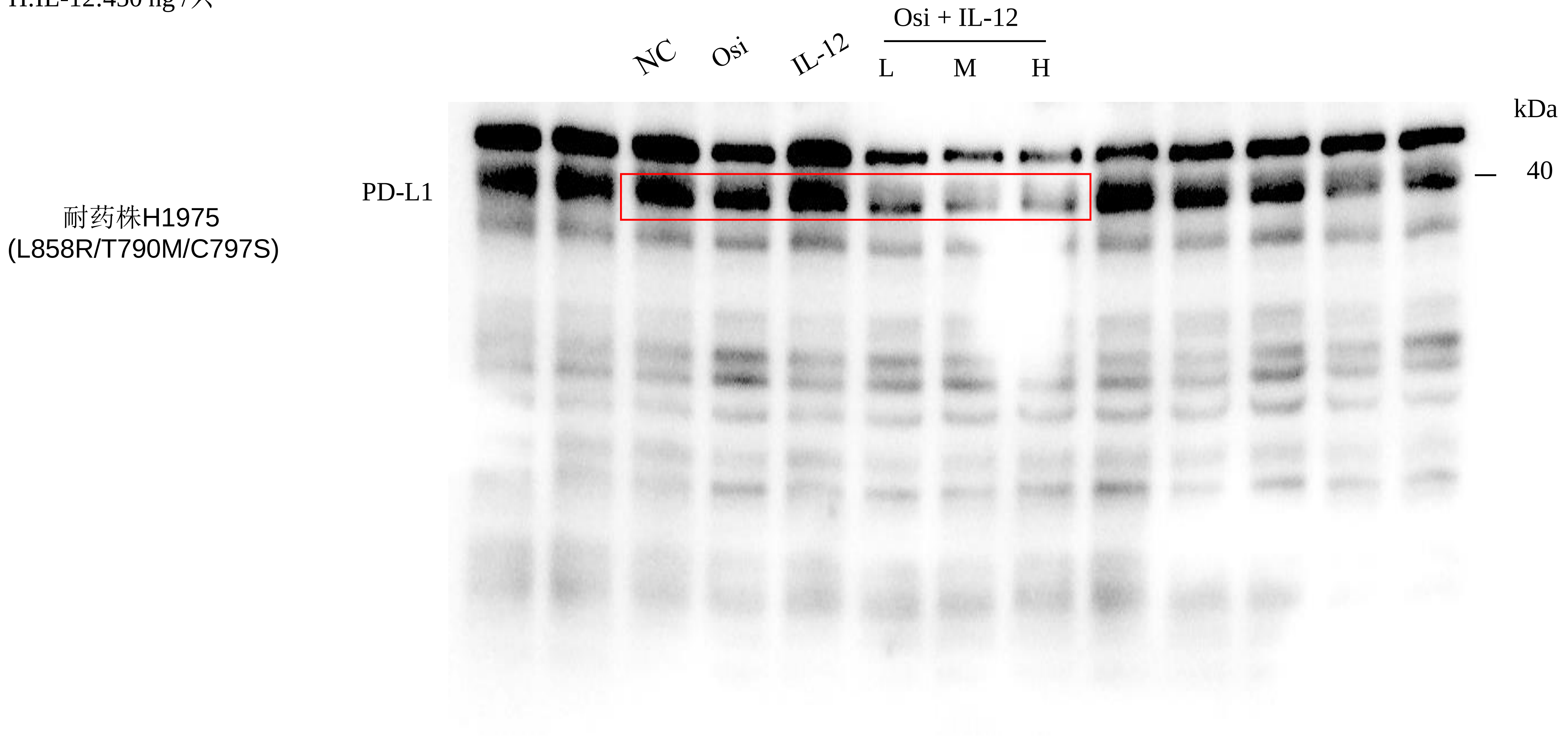

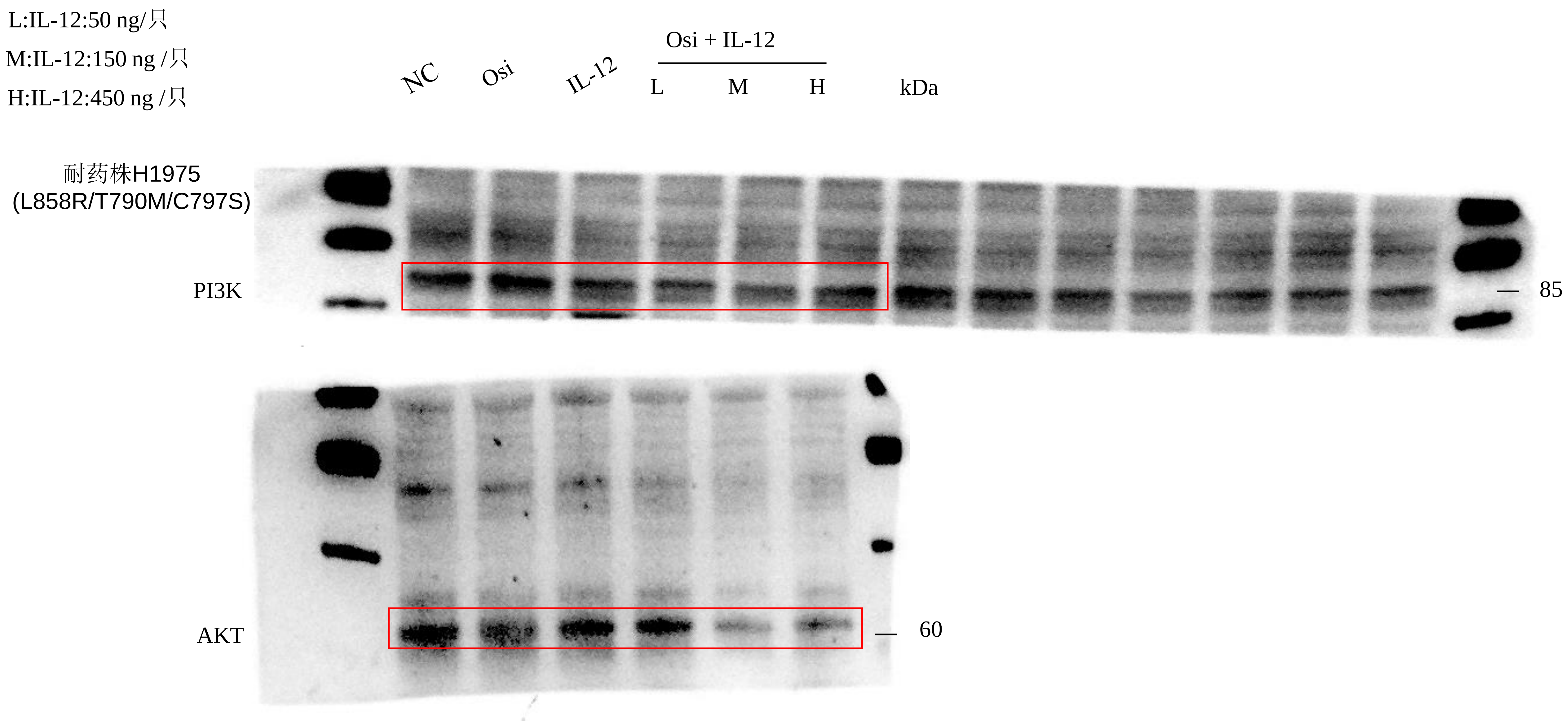
L:IL-12:50 ng/只
M:IL-12:150 ng /只
H:IL-12:450 ng /只
Osi + IL-12
NC
Osi
IL-12
L
M
H
kDa
耐药株H1975
(L858R/T790M/C797S)
PI3K
85
AKT
60

L:IL-12:50 ng/只
M:IL-12:150 ng /只
H:IL-12:450 ng /只

耐药株H1975
(L858R/T790M/C797S

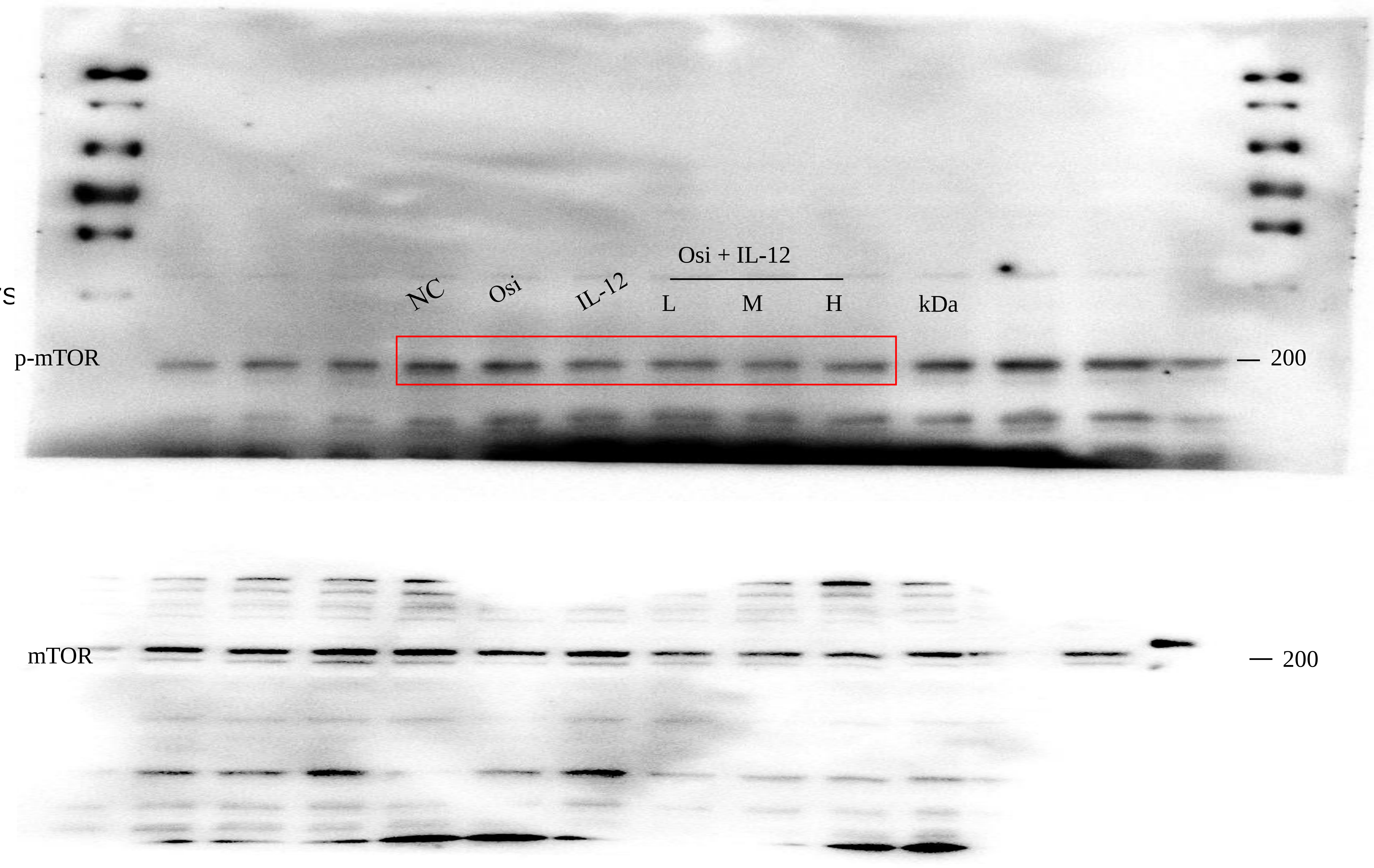

L:IL-12:50 ng/只

M:IL-12:150 ng /只

H:IL-12:450 ng /只

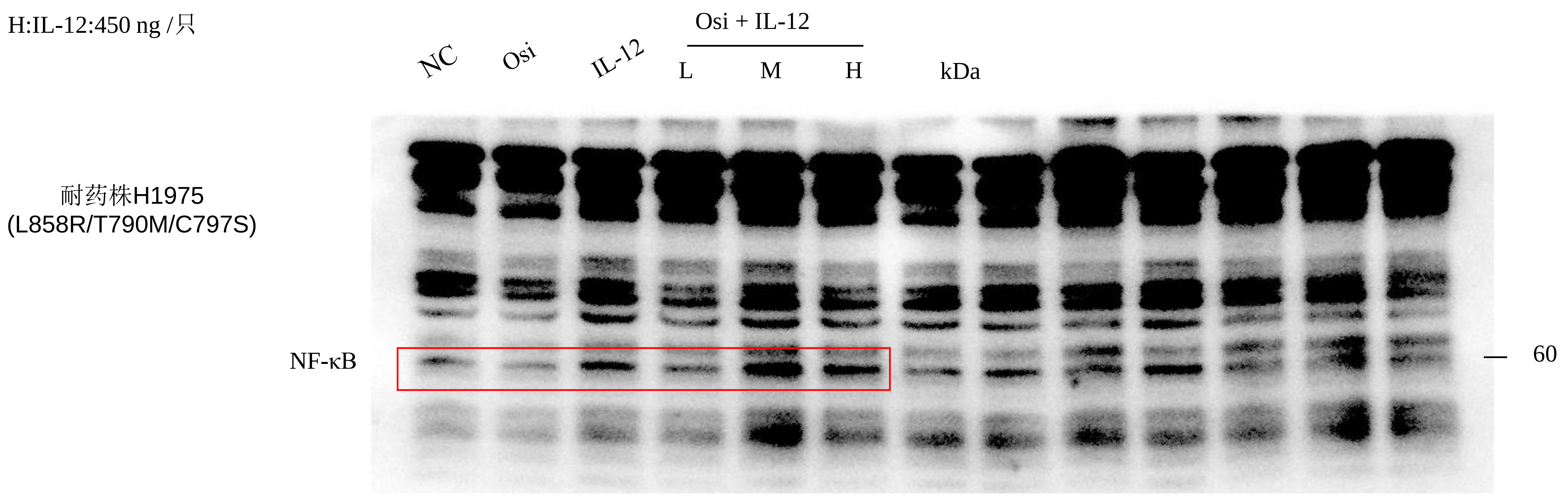

L:IL-12:50 ng/只

M:IL-12:150 ng /只

H:IL-12:450 ng /只

耐药株H1975
(L858R/T790M/C797S)

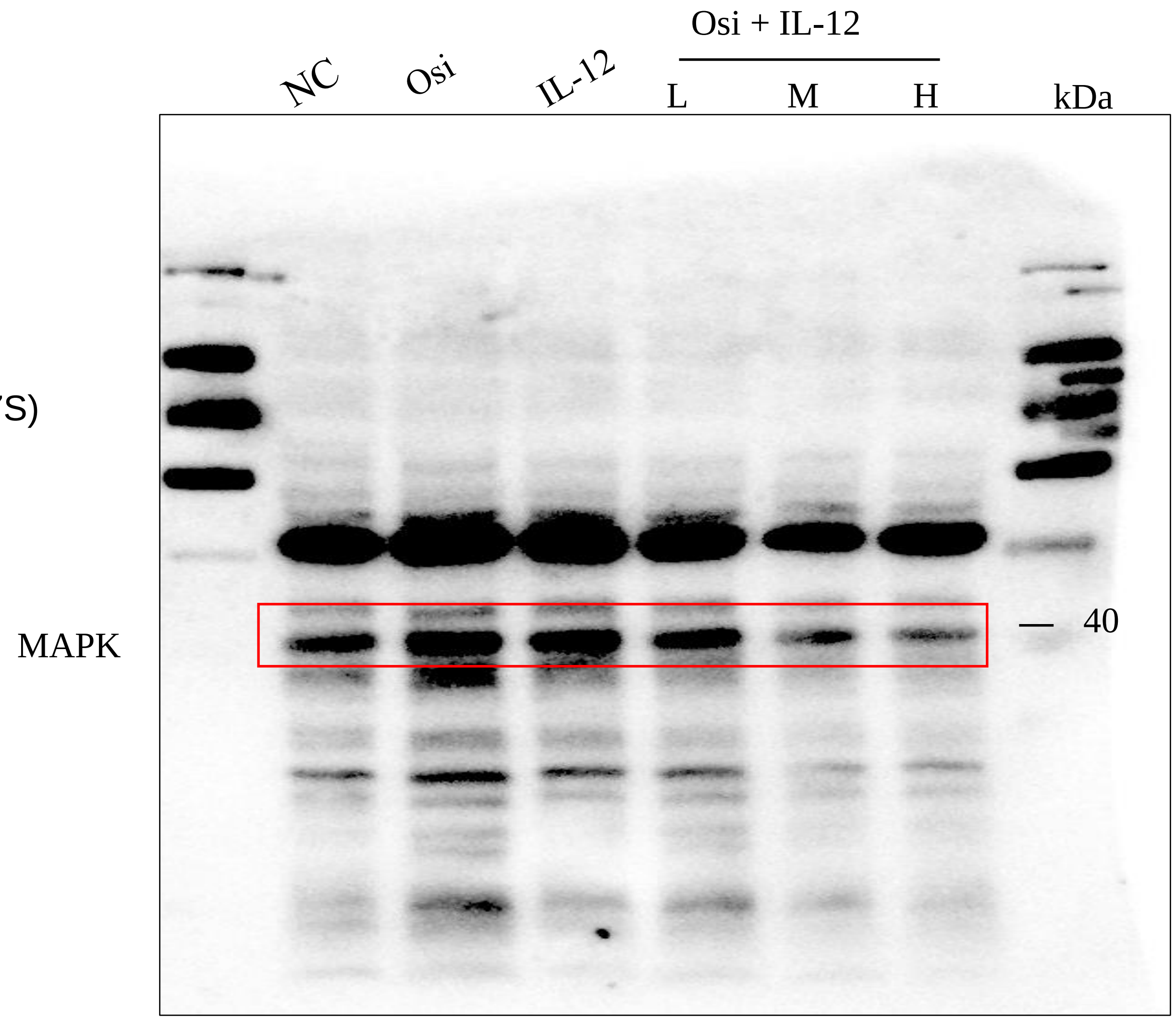

L:IL-12:50 ng/只

M:IL-12:150 ng /只

H:IL-12:450 ng /只

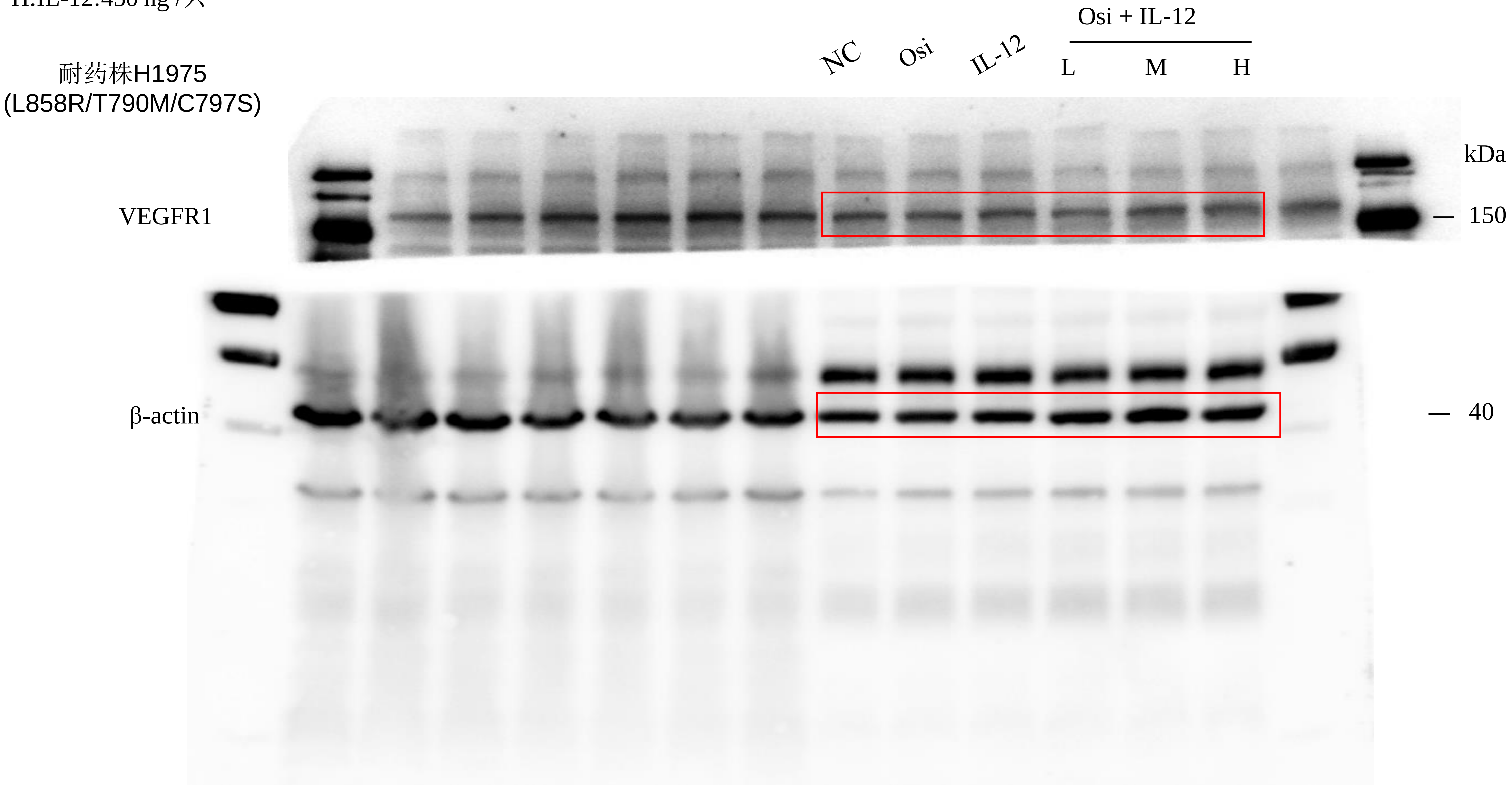

L:IL-12:50 ng/只

M:IL-12:150 ng /只

H:IL-12:450 ng /只

kDa

敏感株H1975
(L858R/T790M)

Osi + IL-12

NC Osi IL-12 L M H

β-actin

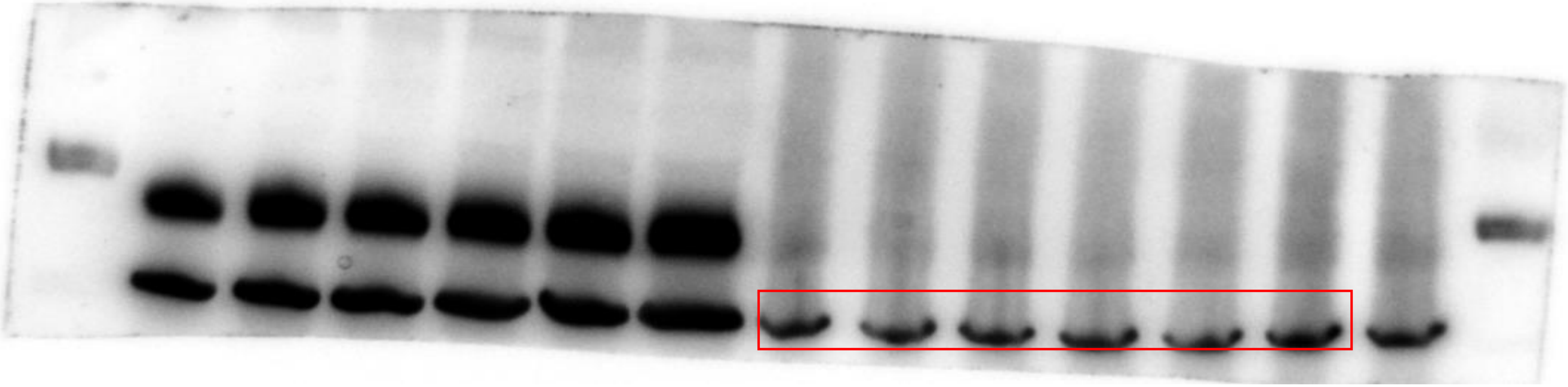

— 40

L:IL-12:50 ng/只

M:IL-12:150 ng /只

H:IL-12:450 ng /只

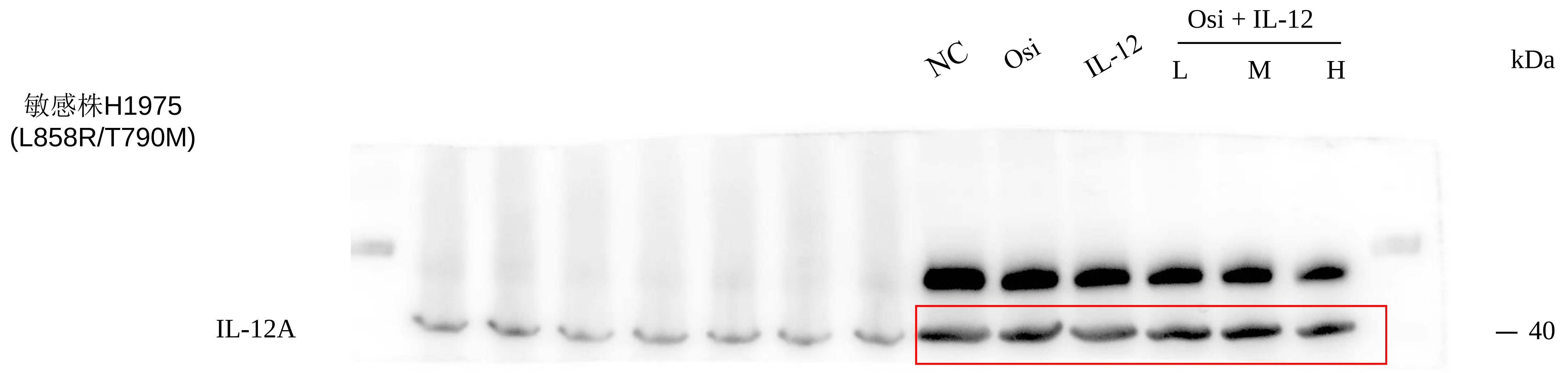

L:IL-12:50 ng/只

M:IL-12:150 ng /只

H:IL-12:450 ng /只

敏感株H1975
(L858R/T790M)

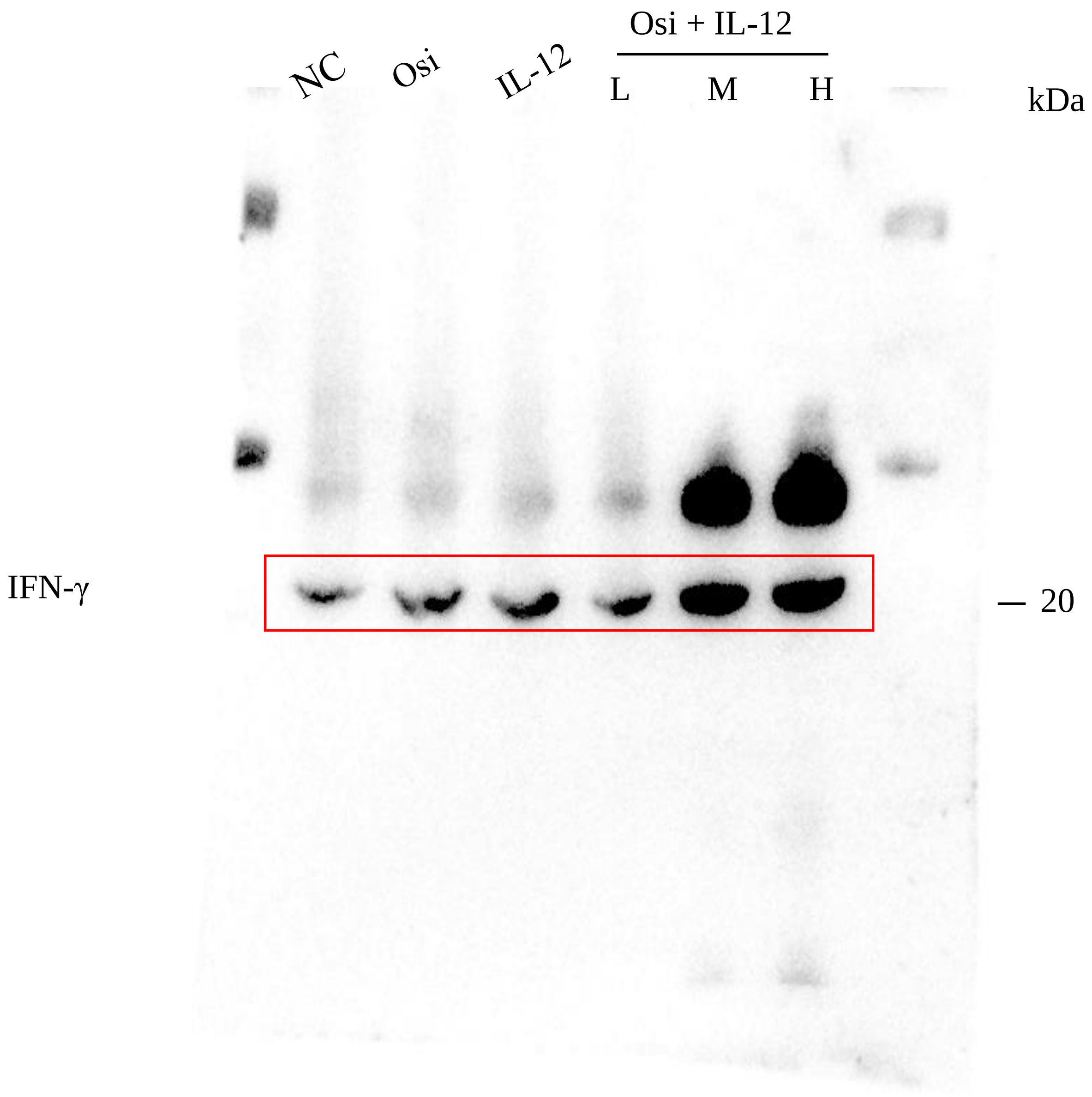

L:IL-12:50 ng/只

M:IL-12:150 ng /只

H:IL-12:450 ng /只

敏感株H1975
(L858R/T790M)

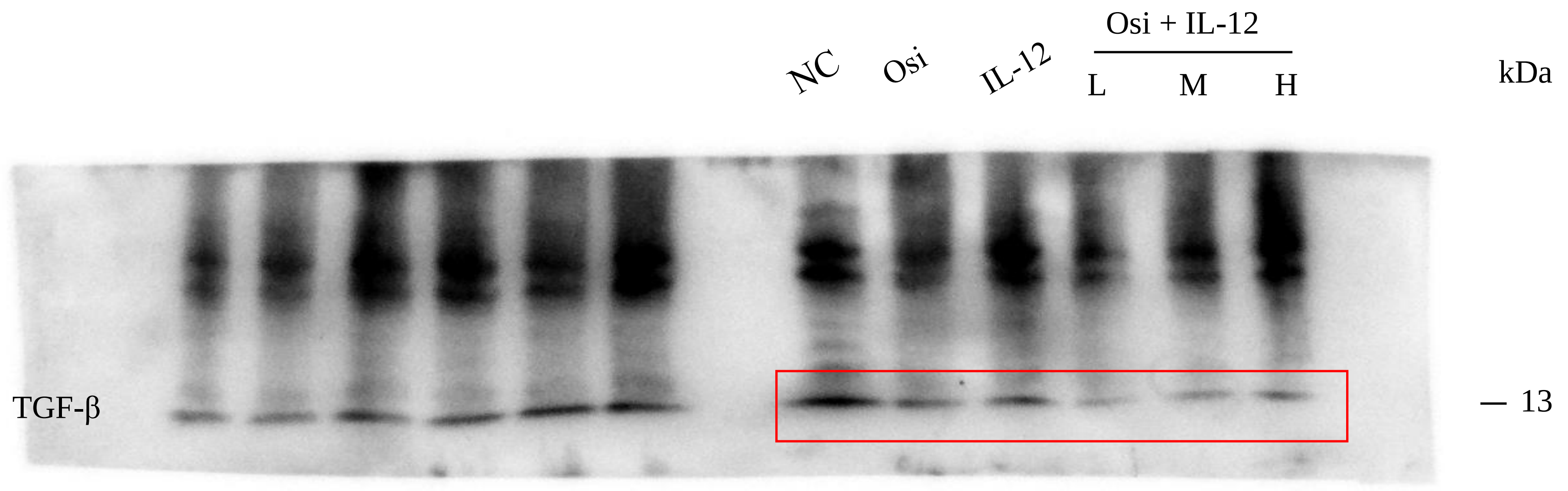

L:IL-12:50 ng/只

M:IL-12:150 ng /只

H:IL-12:450 ng /只

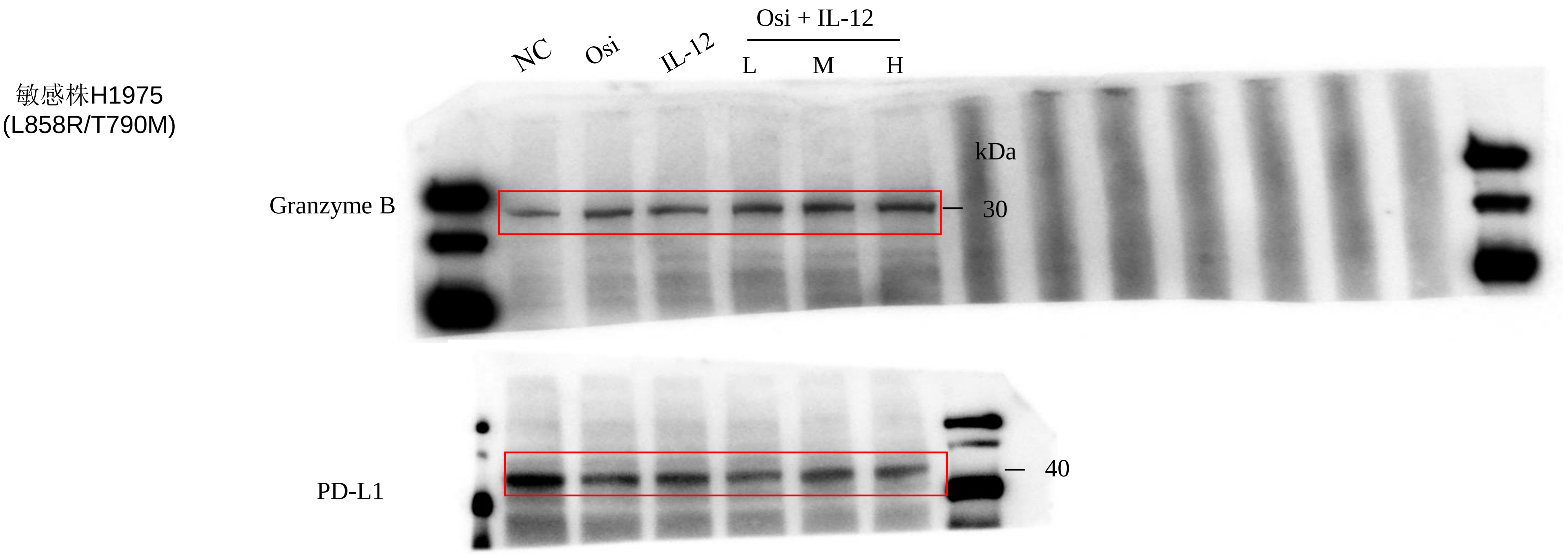

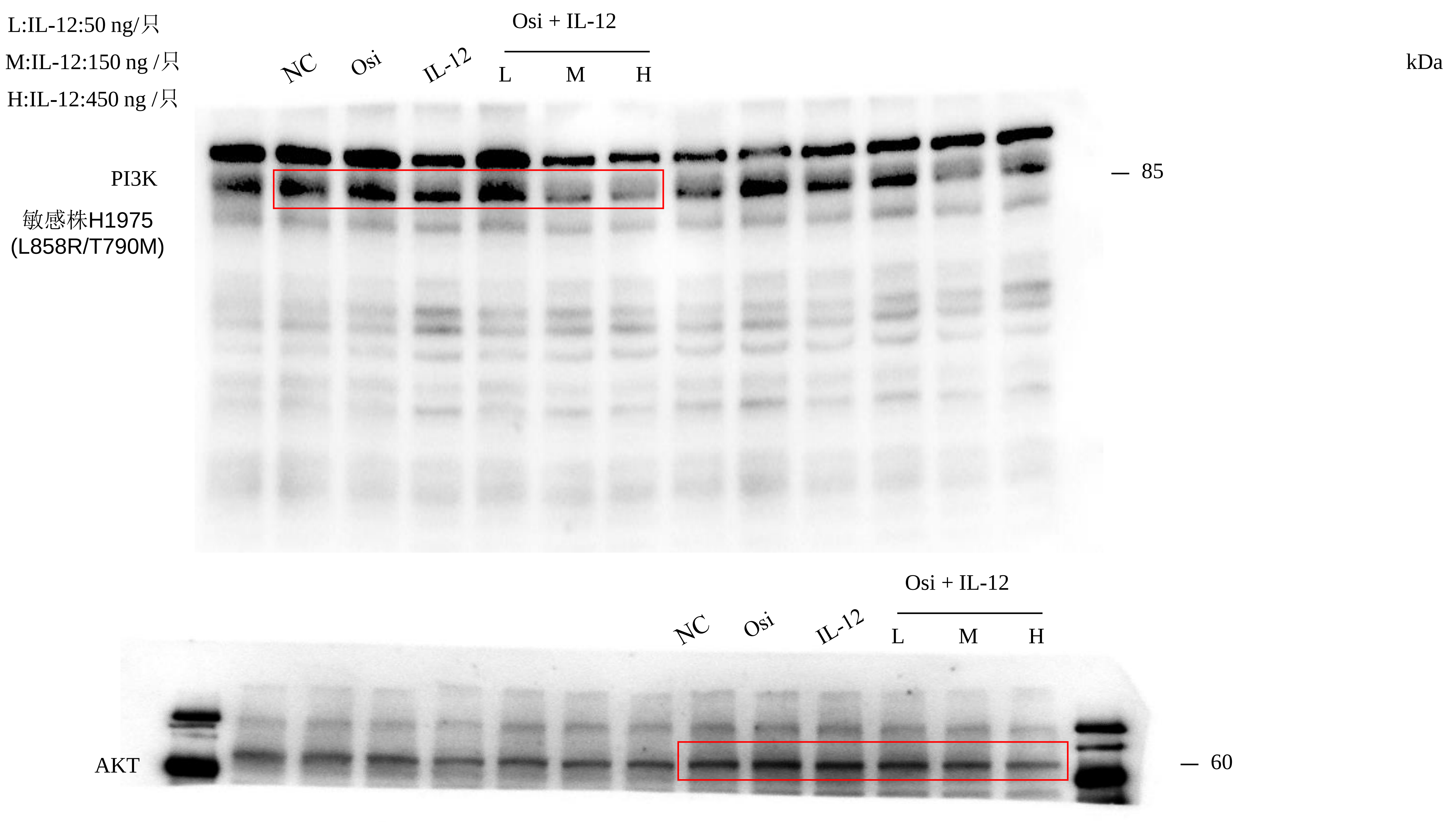
L:IL-12:50 ng/只
M:IL-12:150 ng /只
H:IL-12:450 ng /只
Osi + IL-12
NC
Osi
IL-12
L
M
H
kDa
PI3K
敏感株H1975
(L858R/T790M)
85
Osi + IL-12
NC
Osi
IL-12
L
M
H
AKT
60

L:IL-12:50 ng/只

M:IL-12:150 ng /只

H:IL-12:450 ng /只

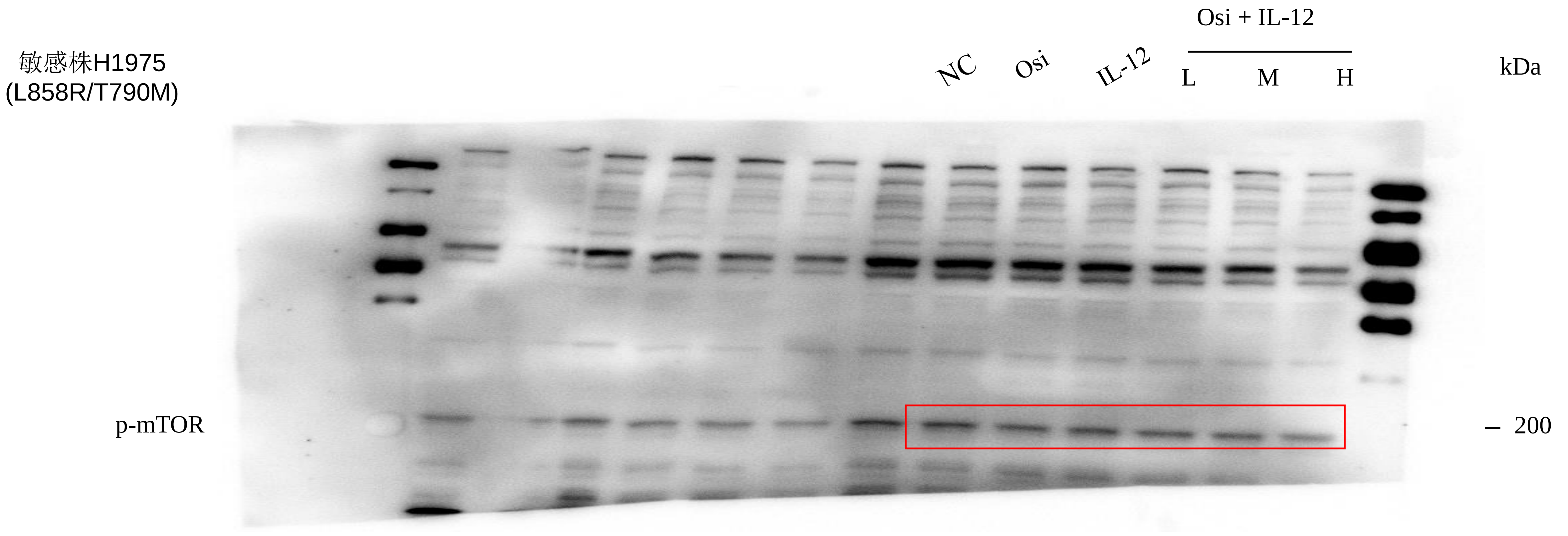

L:IL-12:50 ng/只

M:IL-12:150 ng /只

H:IL-12:450 ng /只

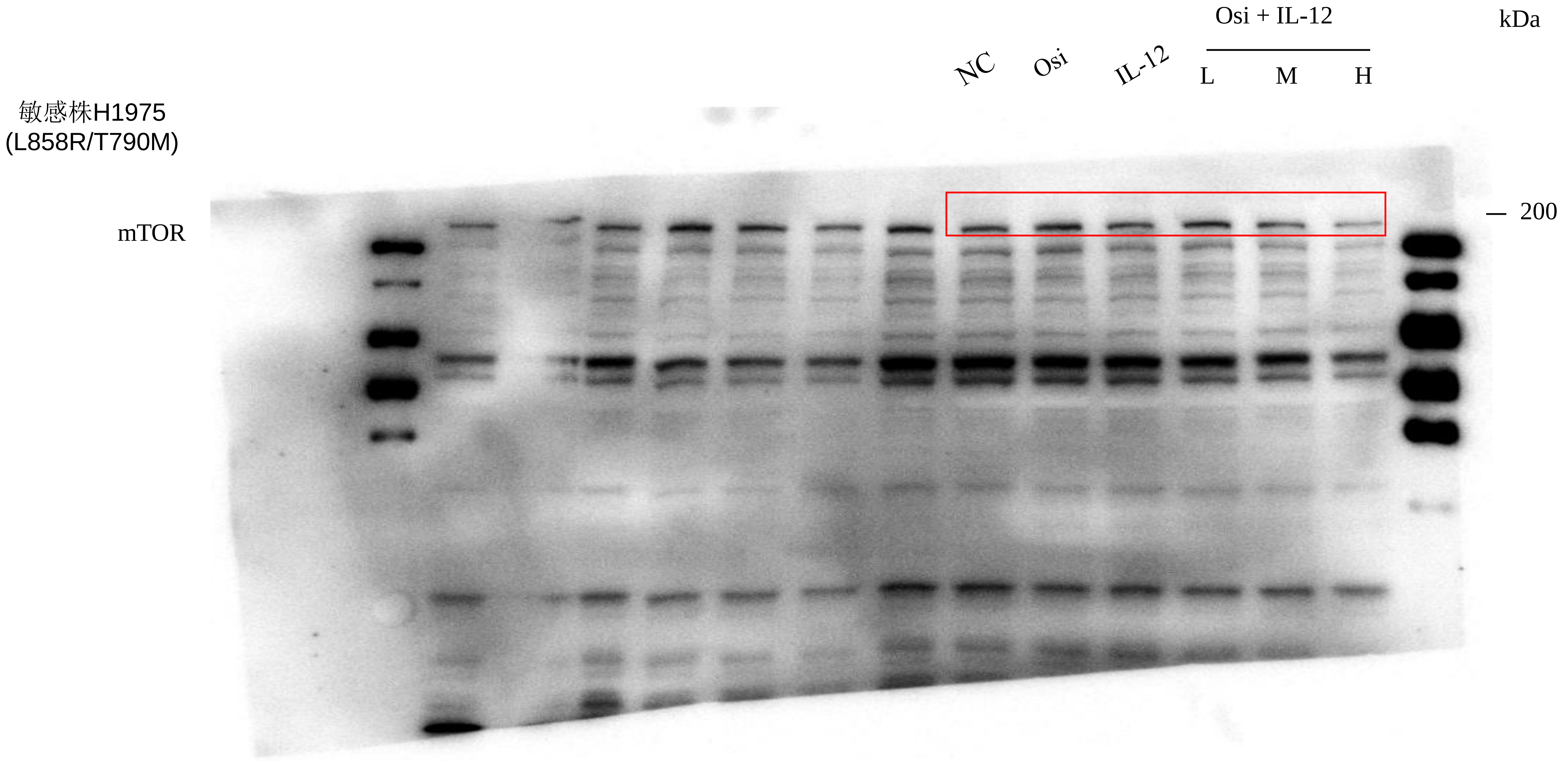

L:IL-12:50 ng/只
M:IL-12:150 ng /只
H:IL-12:450 ng /只

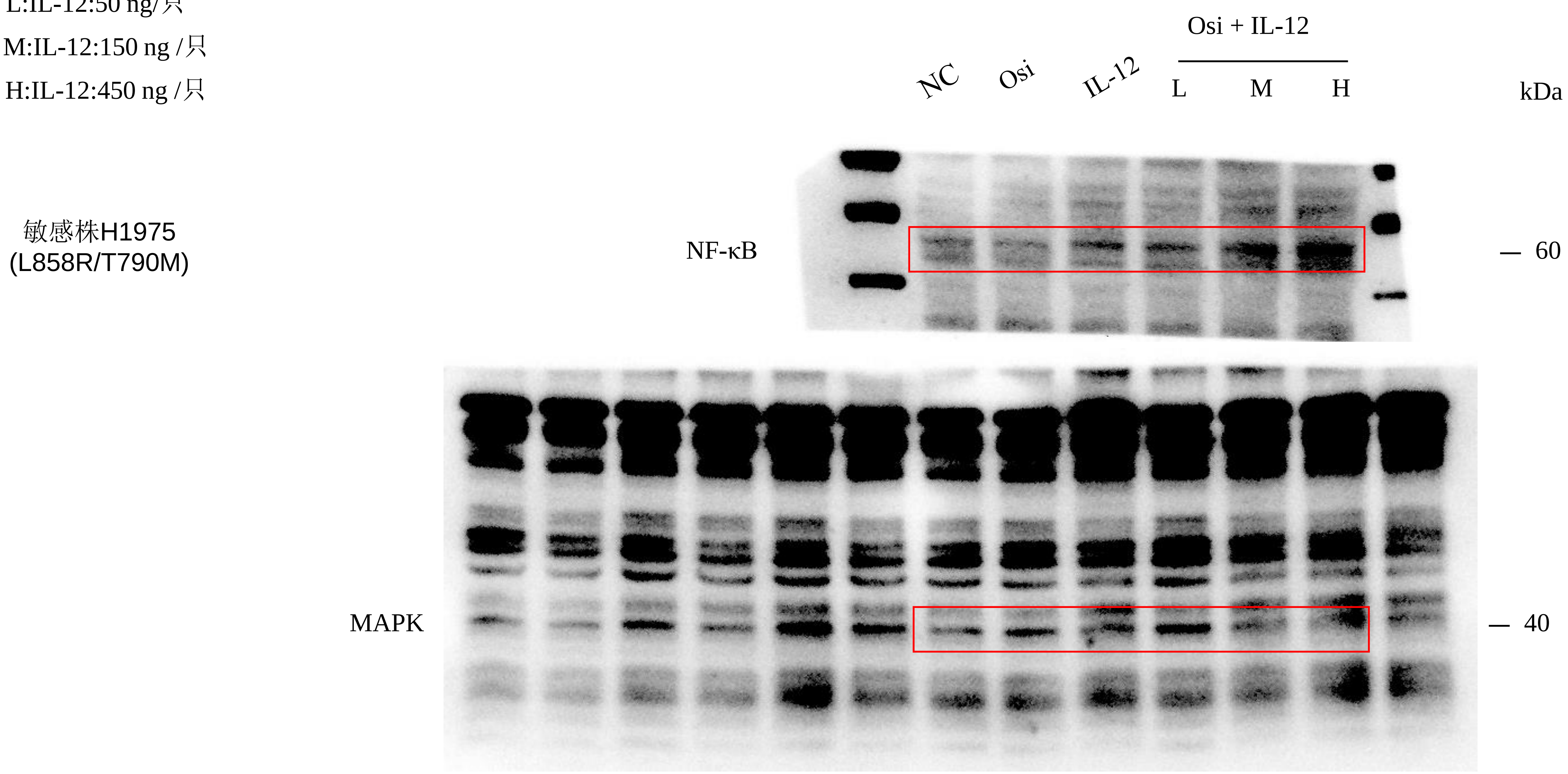

L:IL-12:50 ng/只

M:IL-12:150 ng /只

H:IL-12:450 ng /只

敏感株H1975
(L858R/T790M)

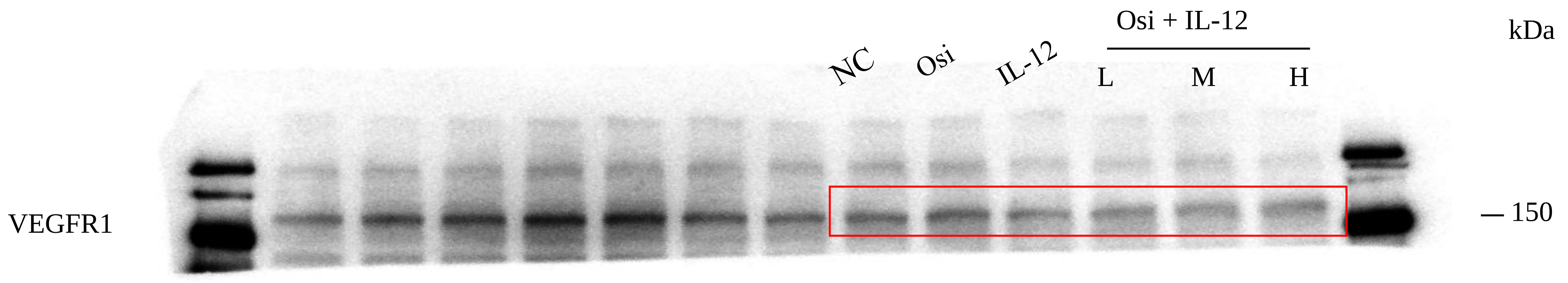

Figure 2

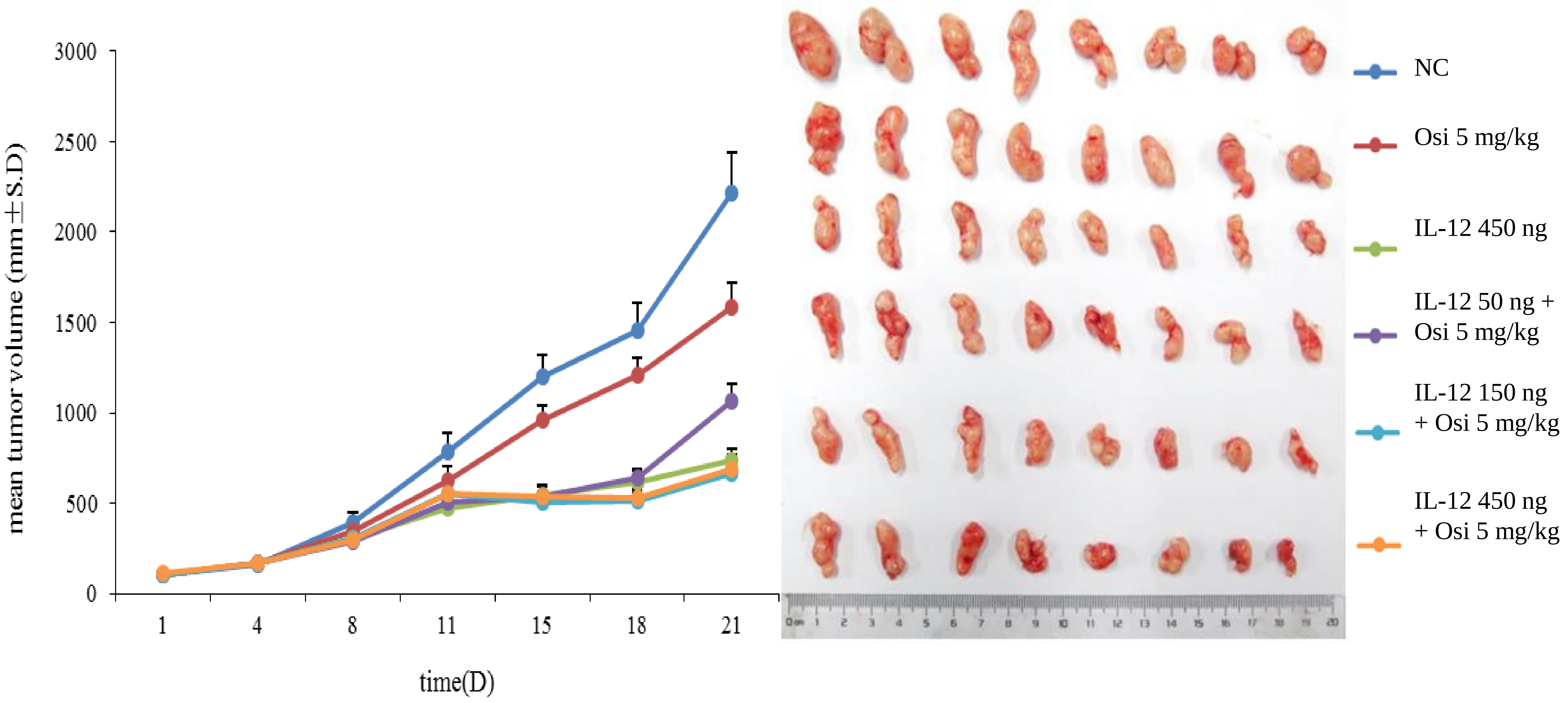
3000
2500
2000
1500
1000
500
0
mean tumor volume (mm±S.D)
1
4
8
11
15
18
21
time(D)
NC
Osi 5 mg/kg
IL-12 450 ng
IL-12 50 ng + Osi 5 mg/kg
IL-12 150 ng + Osi 5 mg/kg
IL-12 450 ng + Osi 5 mg/kg

Figure 2

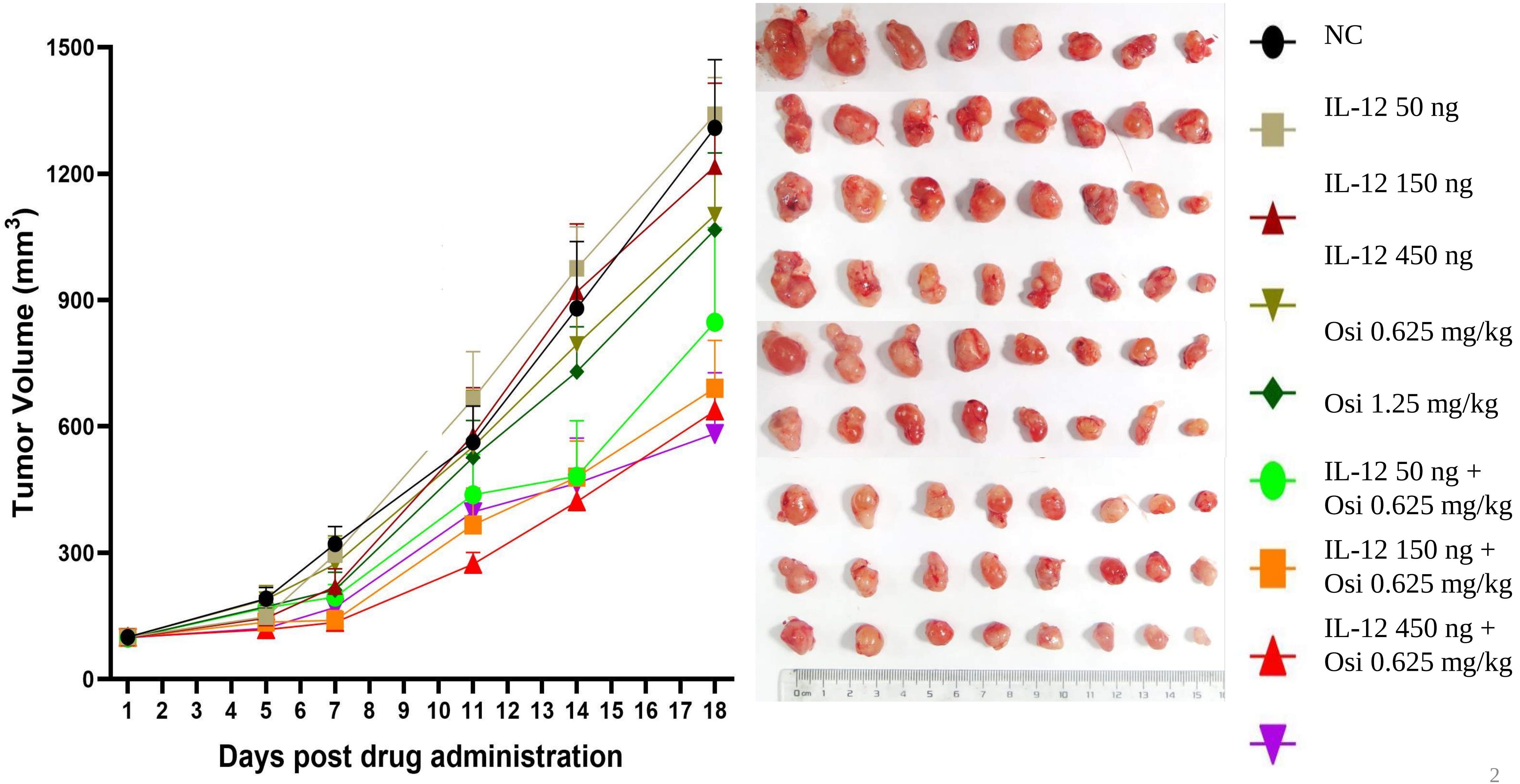

Tumor Volume (mm3)
1500
1200
900
600
300
0
1 2 3 4 5 6 7 8 9 10 11 12 13 14 15 16 17 18
Days post drug administration
NC
IL-12 50 ng
IL-12 150 ng
IL-12 450 ng
Osi 0.625 mg/kg
Osi 1.25 mg/kg
IL-12 50 ng + Osi 0.625 mg/kg
IL-12 150 ng + Osi 0.625 mg/kg
IL-12 450 ng + Osi 0.625 mg/kg

Figure 3

(a)

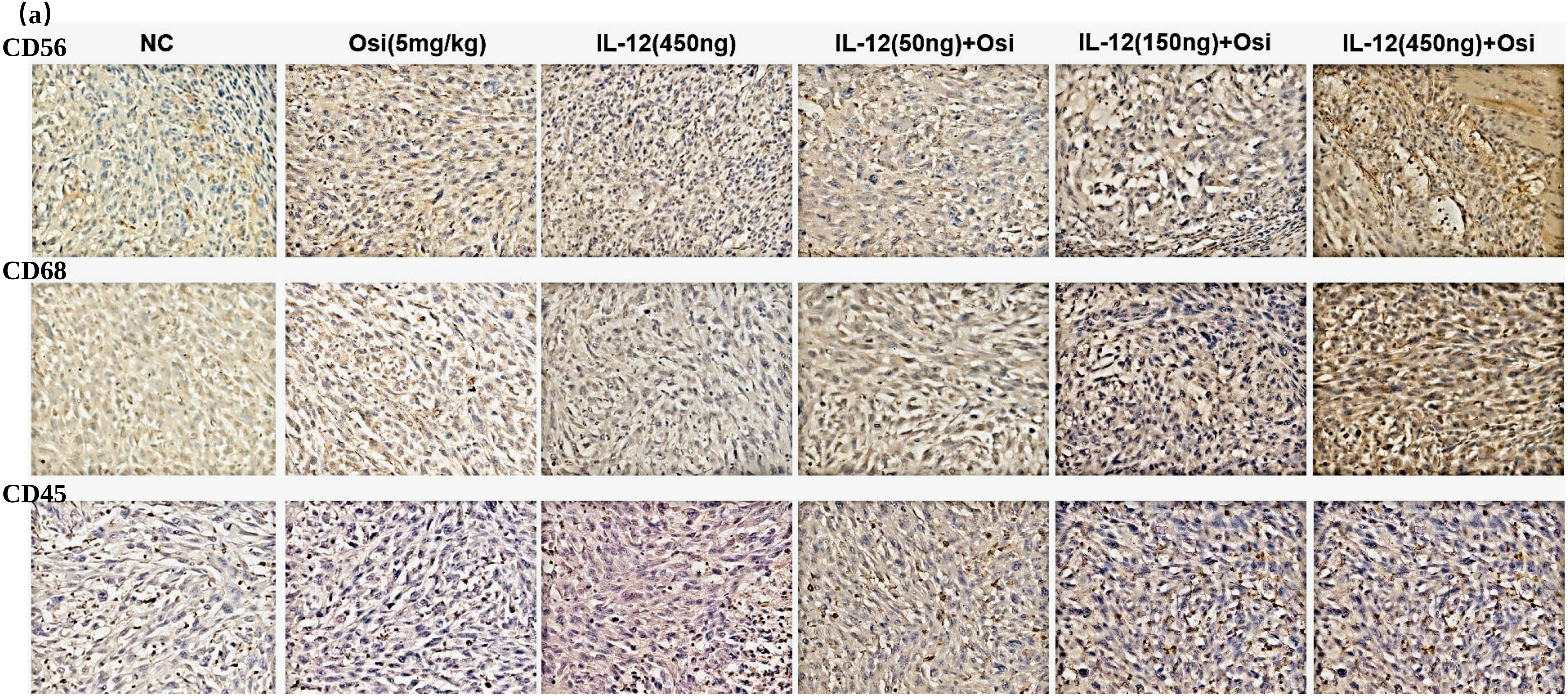

CD56
NC
Osi(5mg/kg)
IL-12(450ng)
IL-12(50ng)+Osi
IL-12(150ng)+Osi
IL-12(450ng)+Osi
CD68
CD45

Figure 3

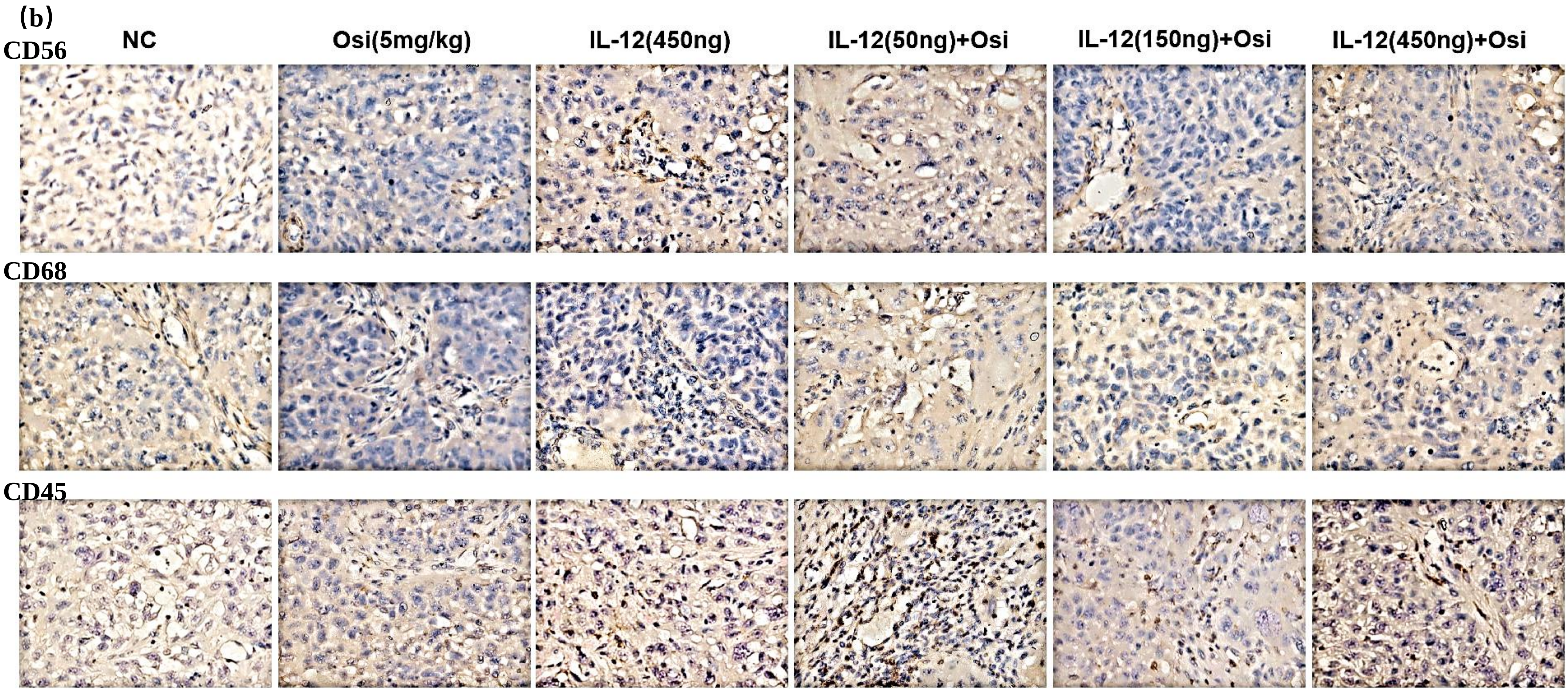
(b)
NC
Osi(5mg/kg)
IL-12(450ng)
IL-12(50ng)+Osi
IL-12(150ng)+Osi
IL-12(450ng)+Osi
CD56
CD68
CD45

Figure 3

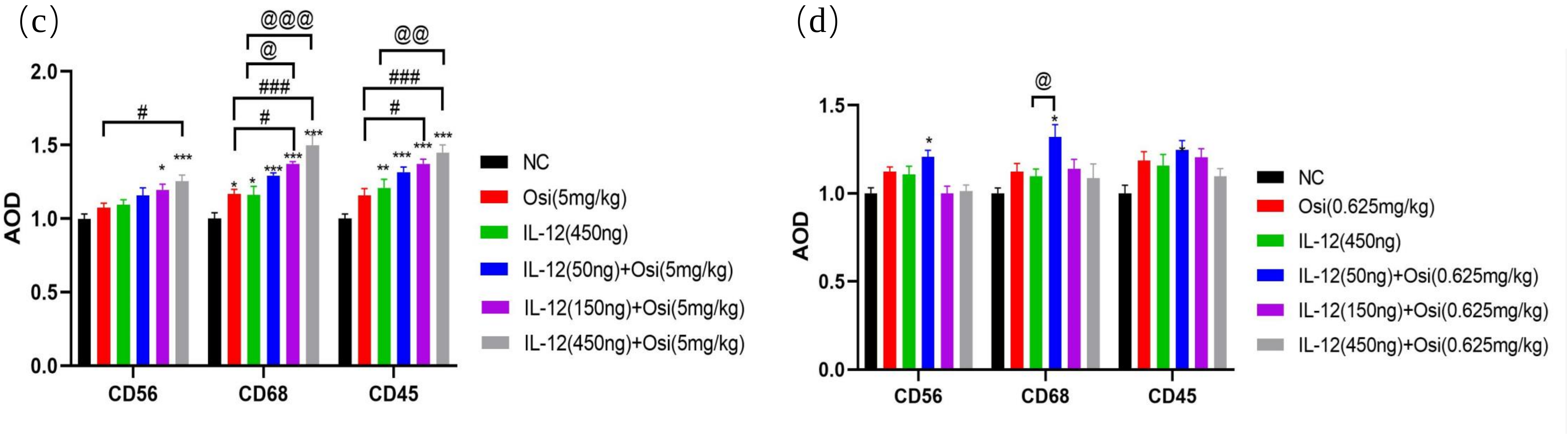
(c)
AOD
2.0
1.5
1.0
0.5
0.0
CD56
CD68
CD45
#
###
@
@@@
@@
*
**
***
NC
Osi(5mg/kg)
IL-12(450ng)
IL-12(50ng)+Osi(5mg/kg)
IL-12(150ng)+Osi(5mg/kg)
IL-12(450ng)+Osi(5mg/kg)
(d)
AOD
1.5
1.0
0.5
0.0
CD56
CD68
CD45
@
*
NC
Osi(0.625mg/kg)
IL-12(450ng)
IL-12(50ng)+Osi(0.625mg/kg)
IL-12(150ng)+Osi(0.625mg/kg)
IL-12(450ng)+Osi(0.625mg/kg)

Figure 4

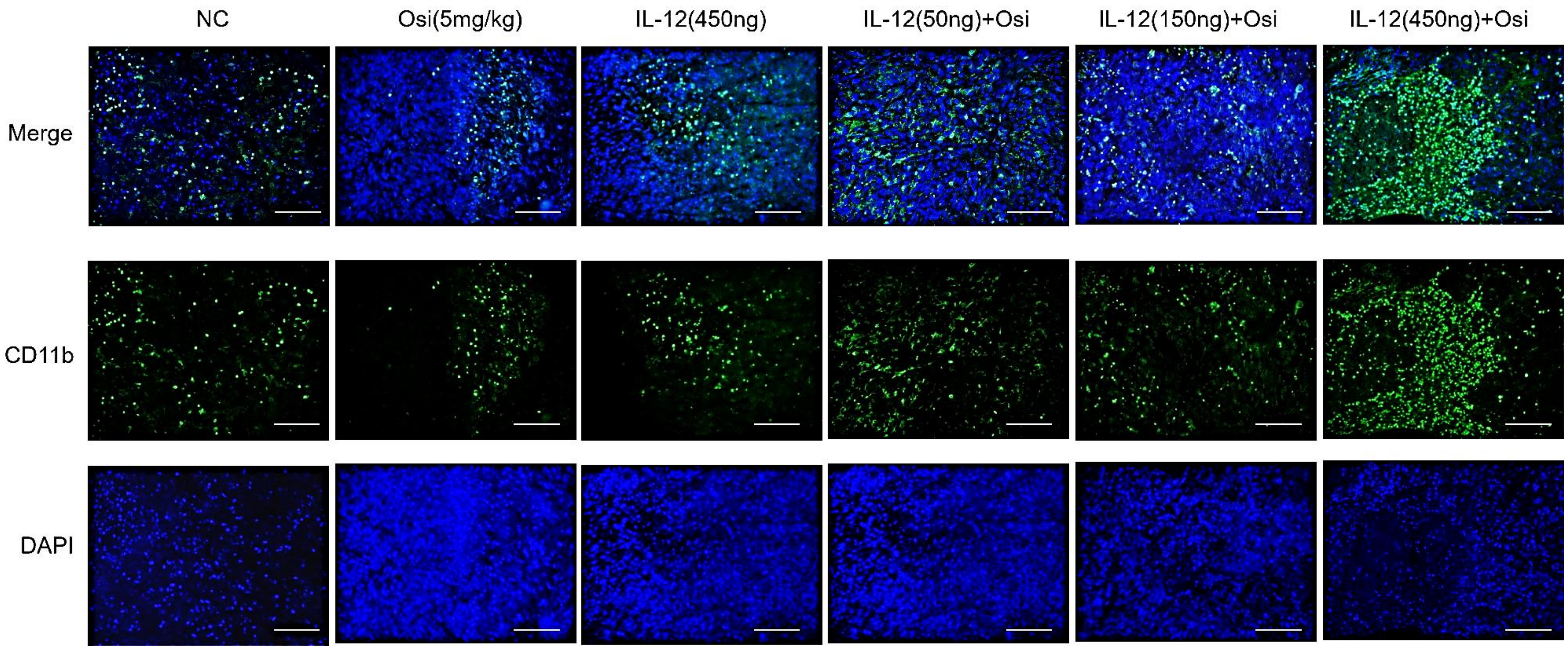
NC
Osi(5mg/kg)
IL-12(450ng)
IL-12(50ng)+Osi
IL-12(150ng)+Osi
IL-12(450ng)+Osi
Merge
CD11b
DAPI

Figure 4

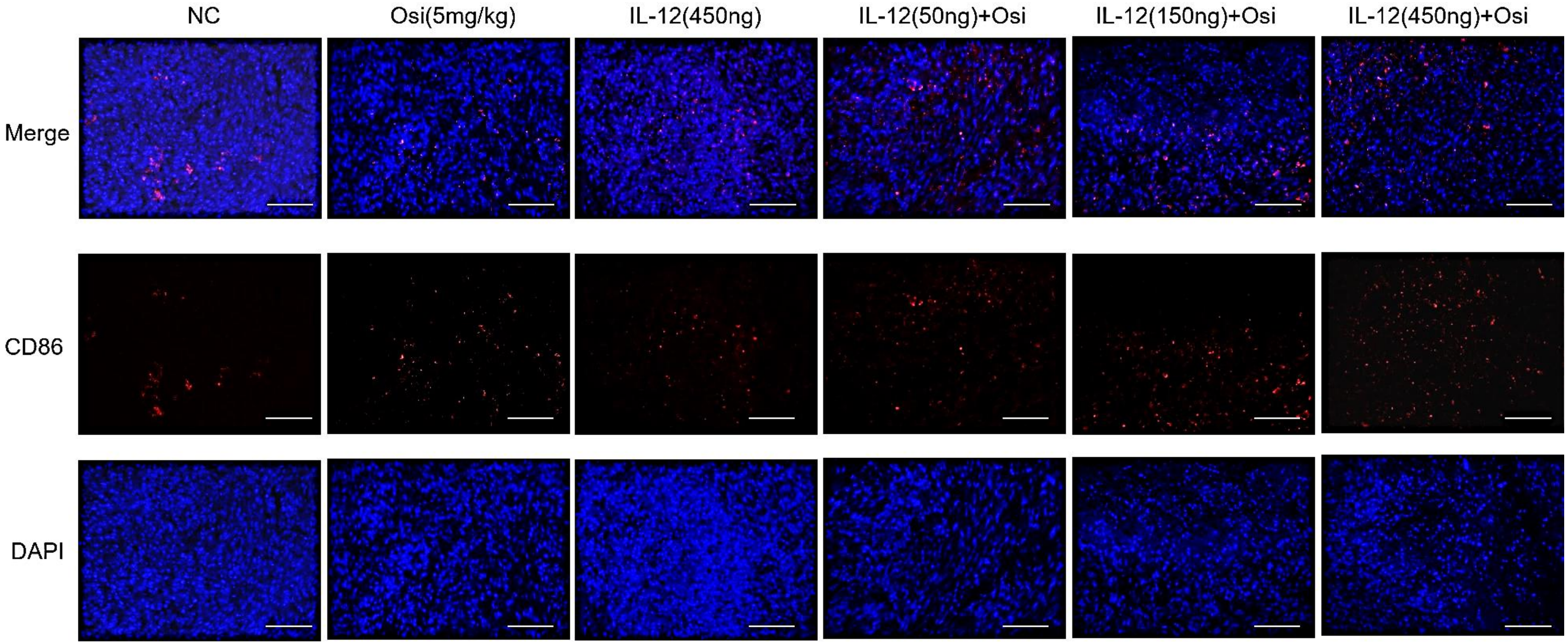
NC
Osi(5mg/kg)
IL-12(450ng)
IL-12(50ng)+Osi
IL-12(150ng)+Osi
IL-12(450ng)+Osi
Merge
CD86
DAPI

Figure 4

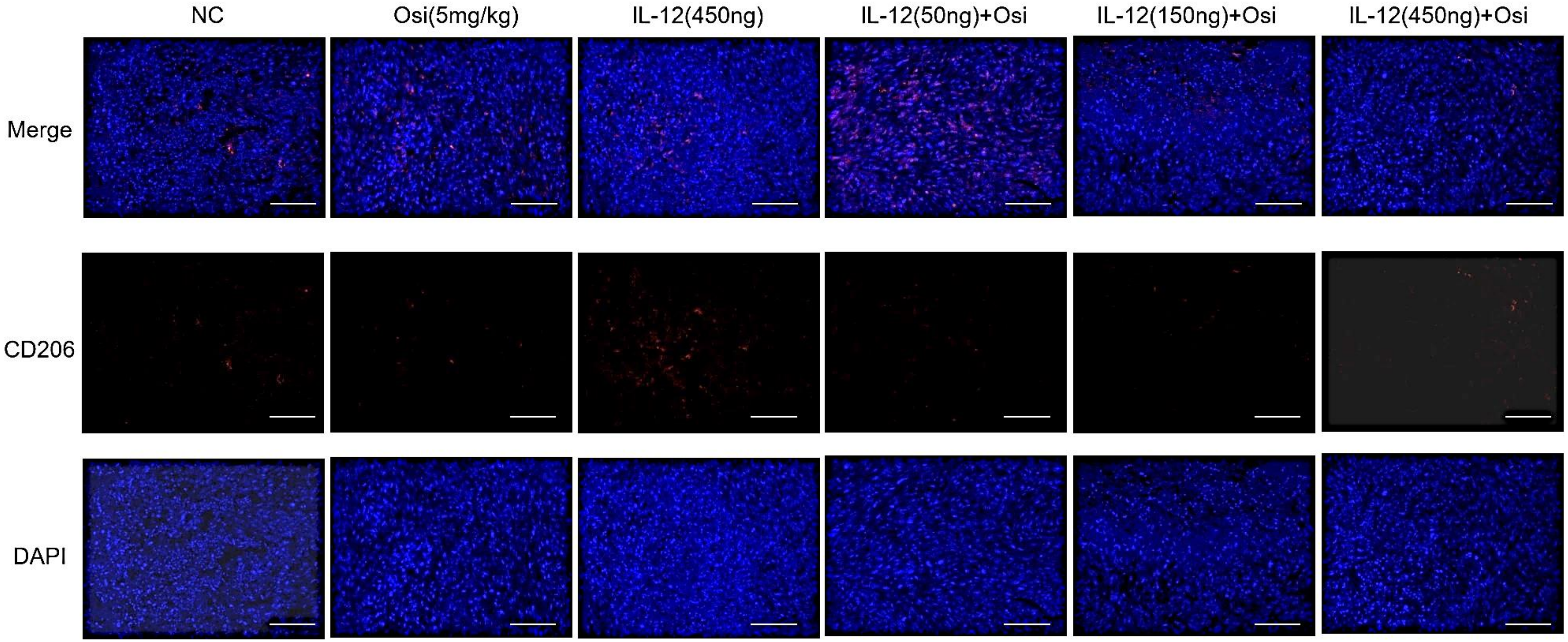
NC
Osi(5mg/kg)
IL-12(450ng)
IL-12(50ng)+Osi
IL-12(150ng)+Osi
IL-12(450ng)+Osi
Merge
CD206
DAPI

Figure 4

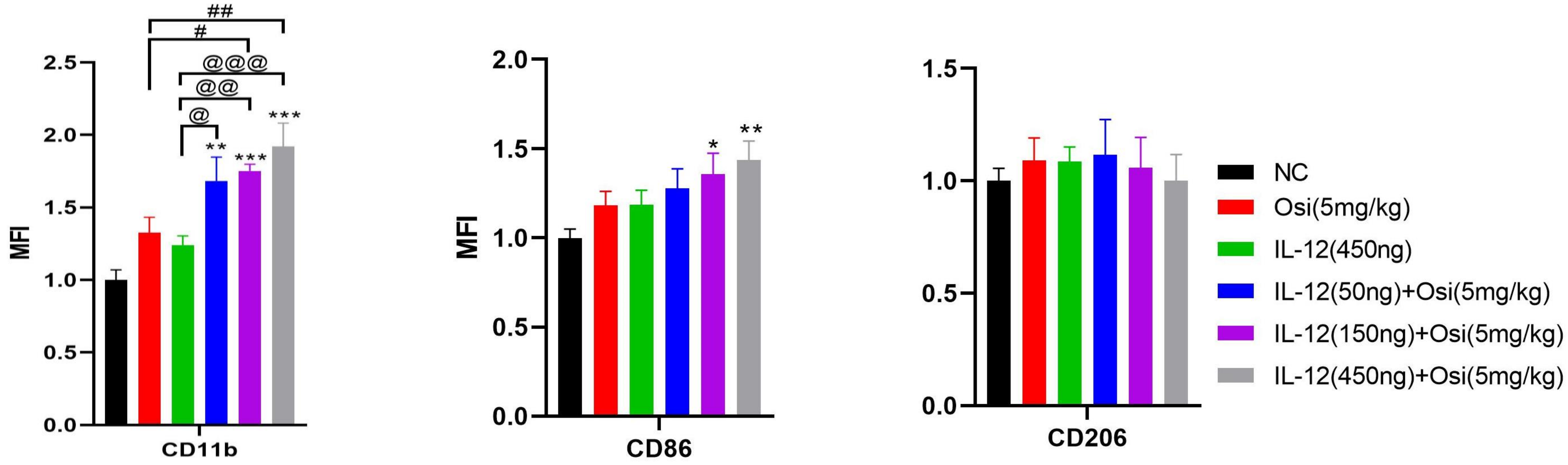

##
#
@@@
@@
@
***
**
***
2.5
2.0
1.5
1.0
0.5
0.0
MFI
CD11b
2.0
1.5
1.0
0.5
0.0
MFI
*
**
CD86
1.5
1.0
0.5
0.0
CD206
NC
Osi(5mg/kg)
IL-12(450ng)
IL-12(50ng)+Osi(5mg/kg)
IL-12(150ng)+Osi(5mg/kg)
IL-12(450ng)+Osi(5mg/kg)

Figure 5

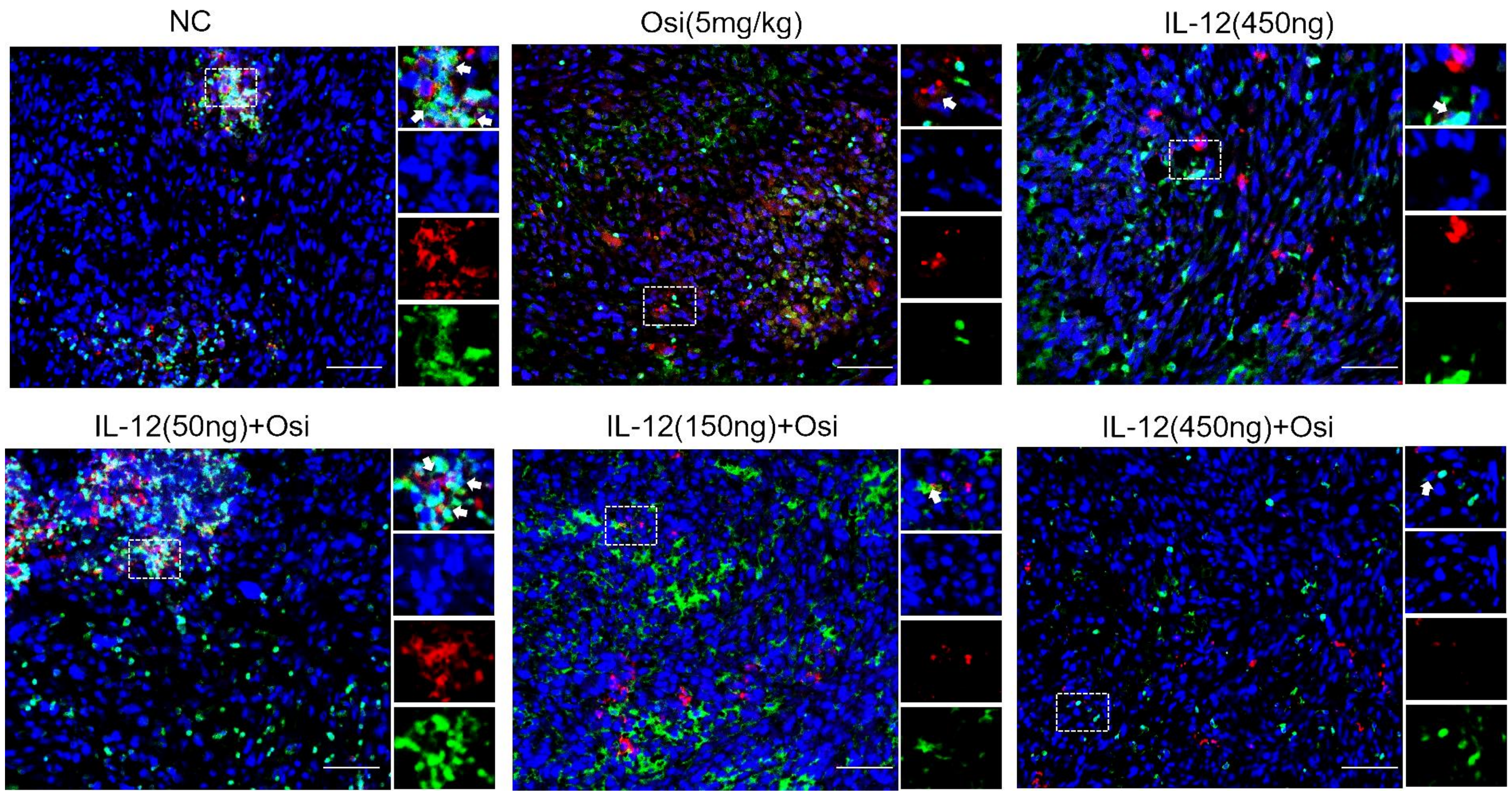
NC
Osi(5mg/kg)
IL-12(450ng)
IL-12(50ng)+Osi
IL-12(150ng)+Osi
IL-12(450ng)+Osi

Figure 6

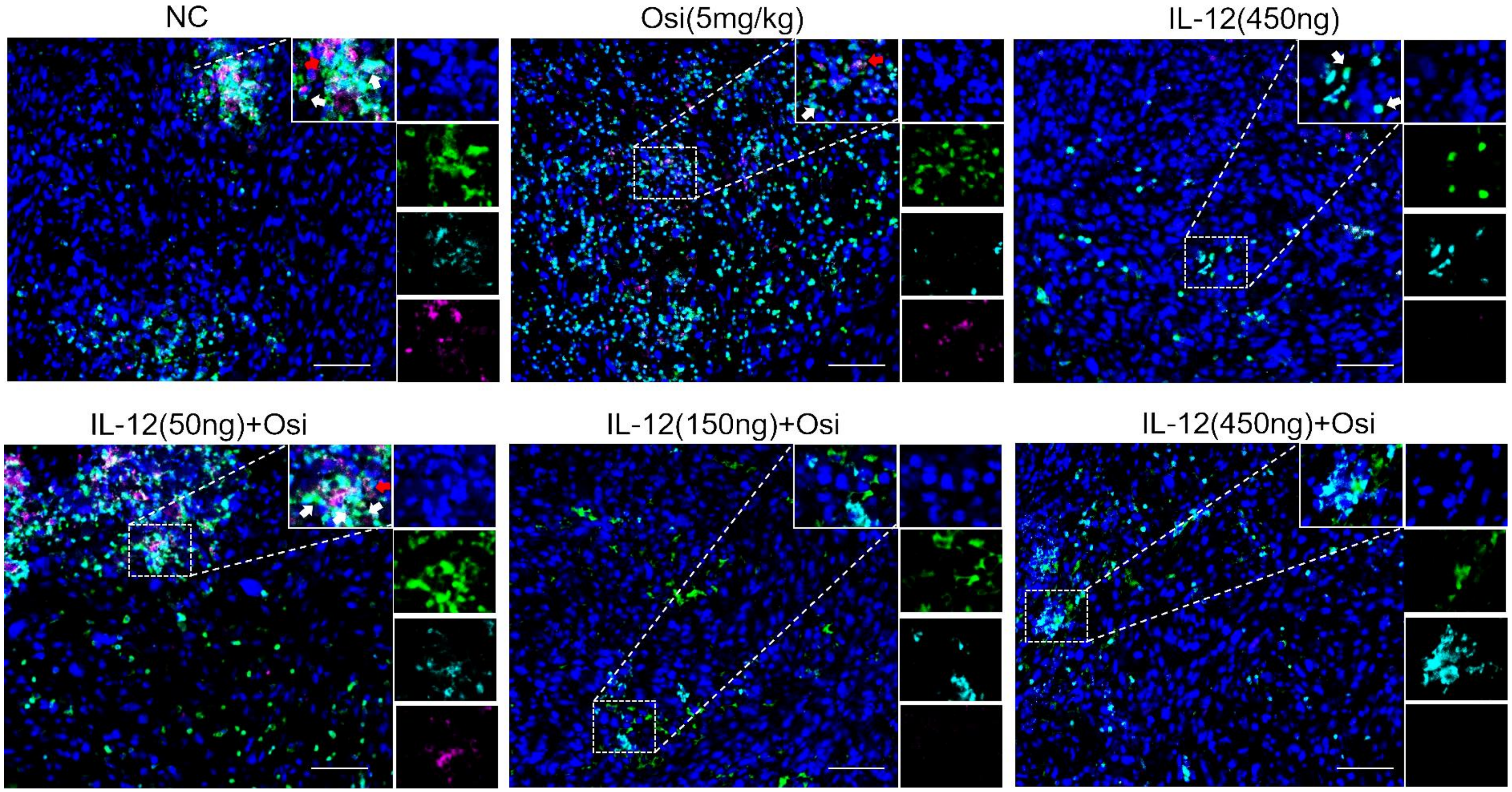
NC
Osi(5mg/kg)
IL-12(450ng)
IL-12(50ng)+Osi
IL-12(150ng)+Osi
IL-12(450ng)+Osi

Figure 9

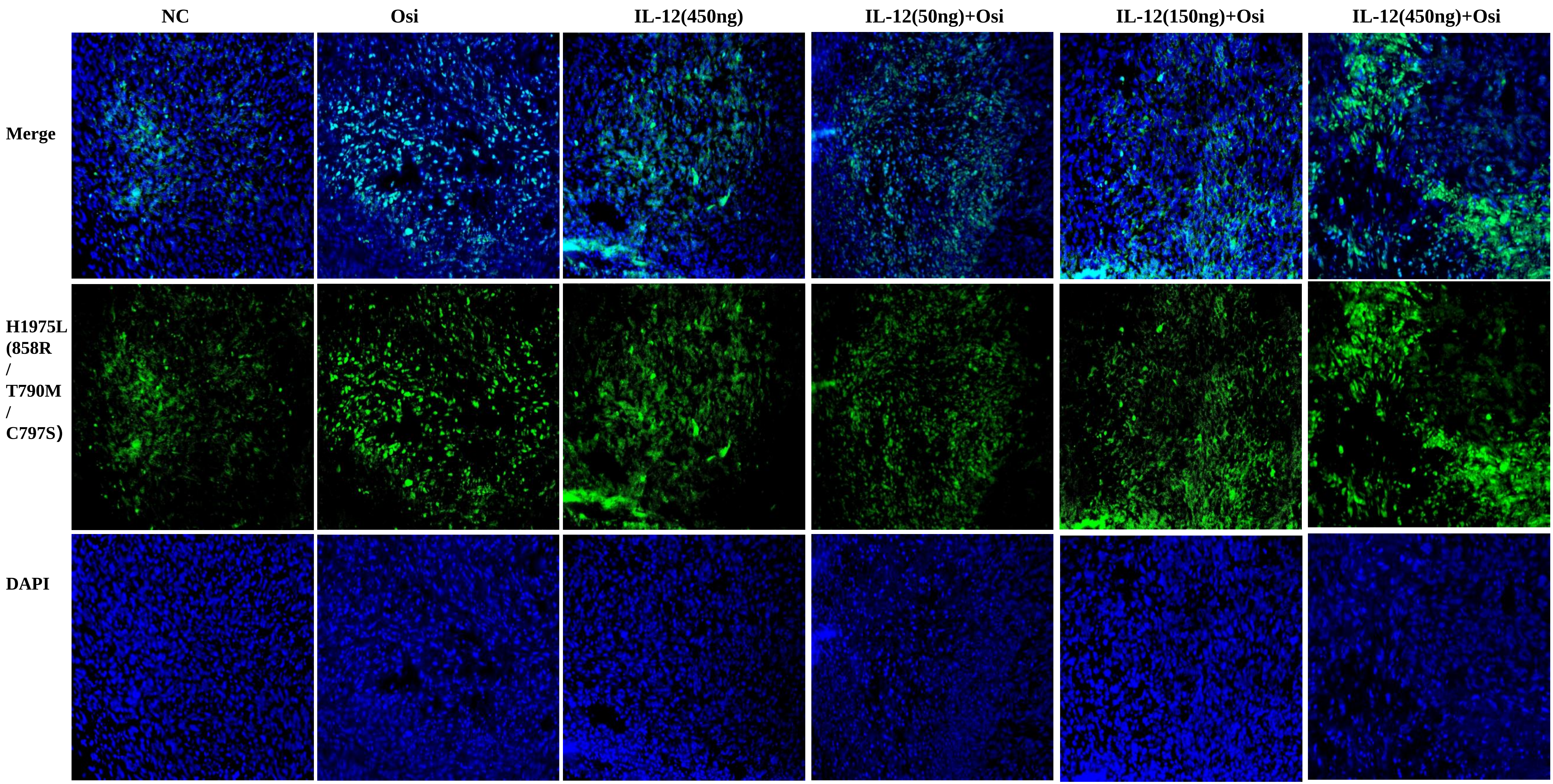
NC
Osi
IL-12(450ng)
IL-12(50ng)+Osi
IL-12(150ng)+Osi
IL-12(450ng)+Osi
Merge
H1975L
(858R
/
T790M
/
C797S)
DAPI

Figure 9

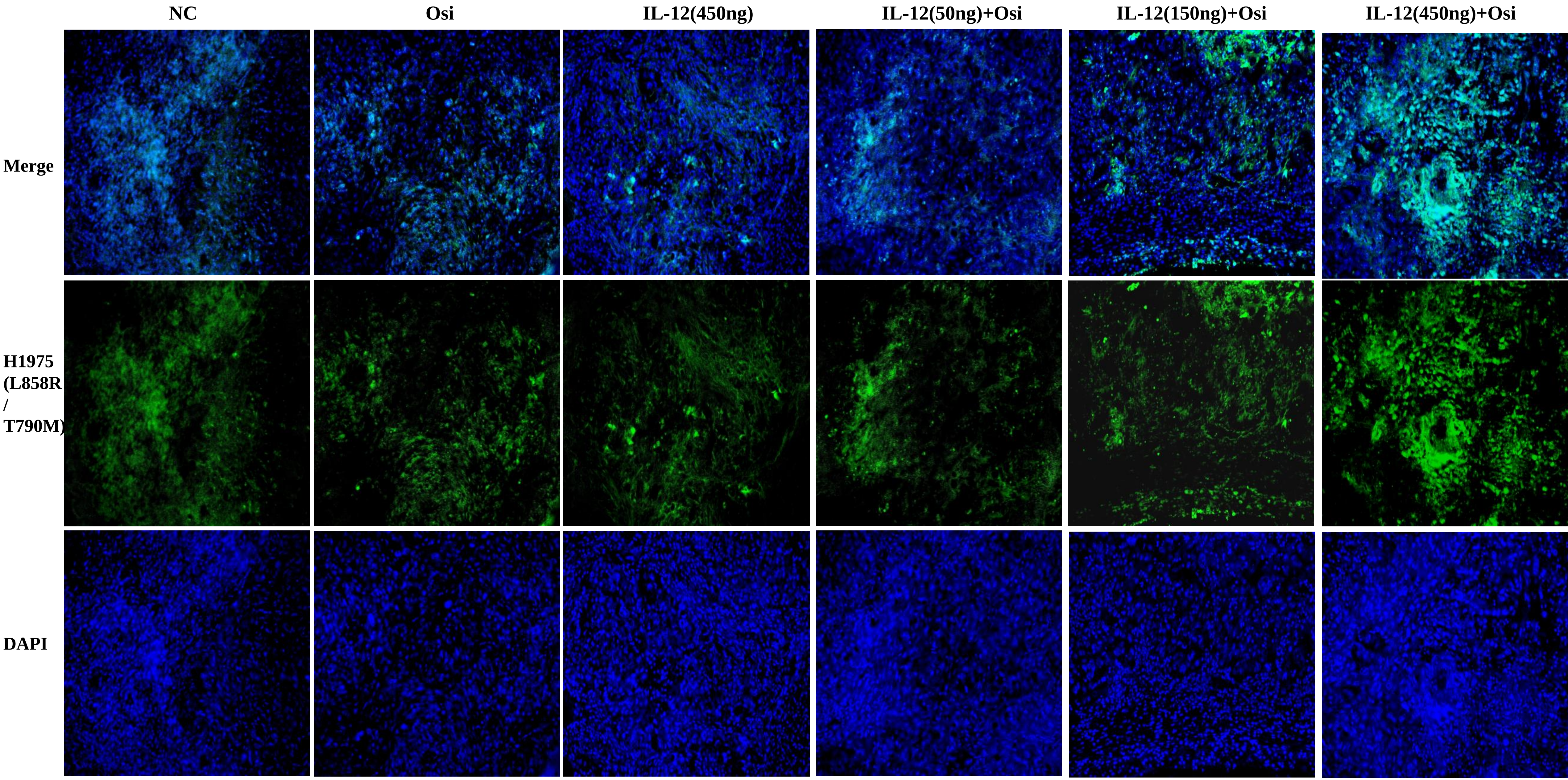
NC
Osi
IL-12(450ng)
IL-12(50ng)+Osi
IL-12(150ng)+Osi
IL-12(450ng)+Osi
Merge
H1975 (L858R/T790M)
DAPI

Figure 9

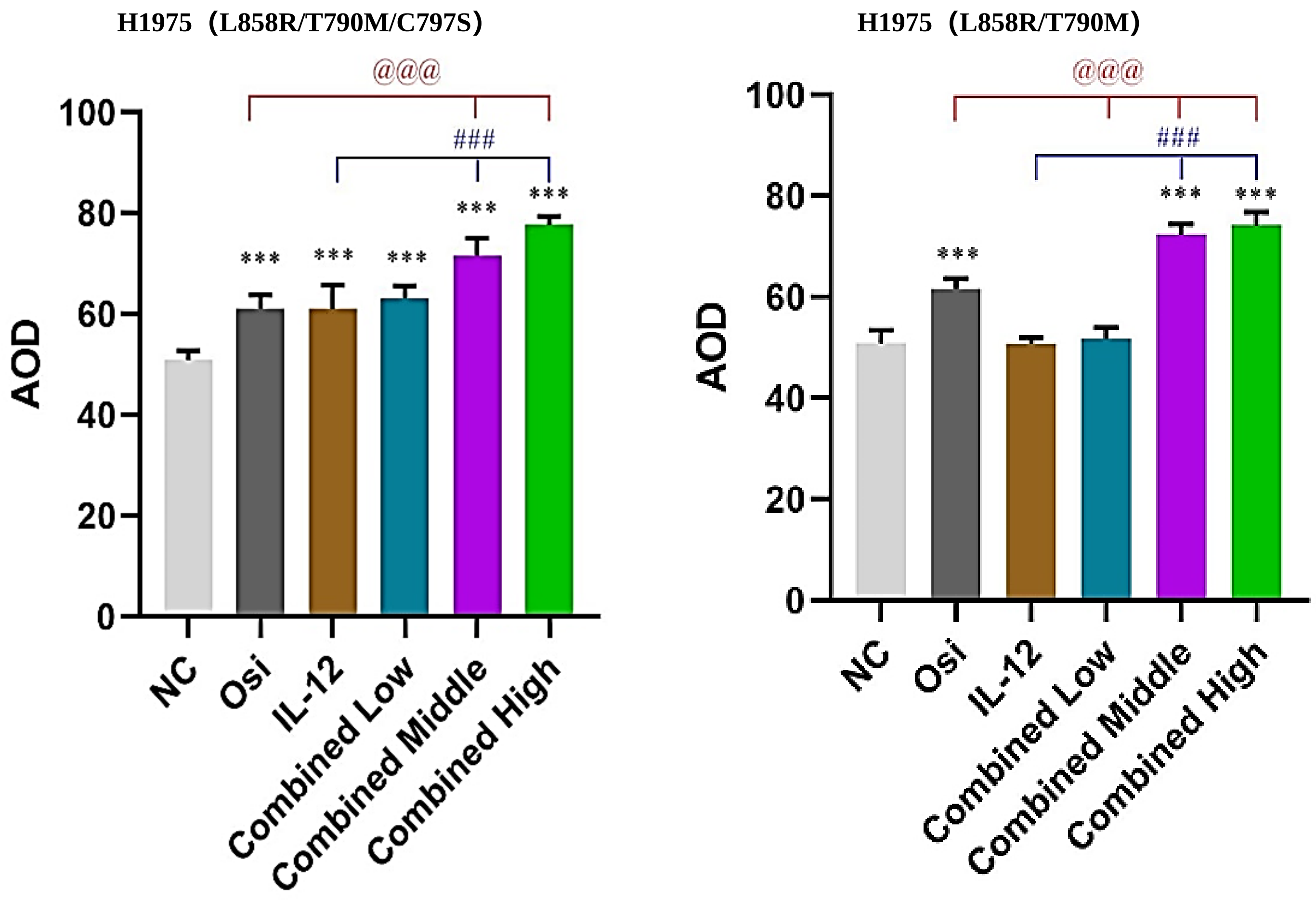
H1975（L858R/T790M/C797S）
H1975（L858R/T790M）
@@@
###
***
AOD
0
20
40
60
80
100
NC
Osi
IL-12
Combined Low
Combined Middle
Combined High

Figure 10

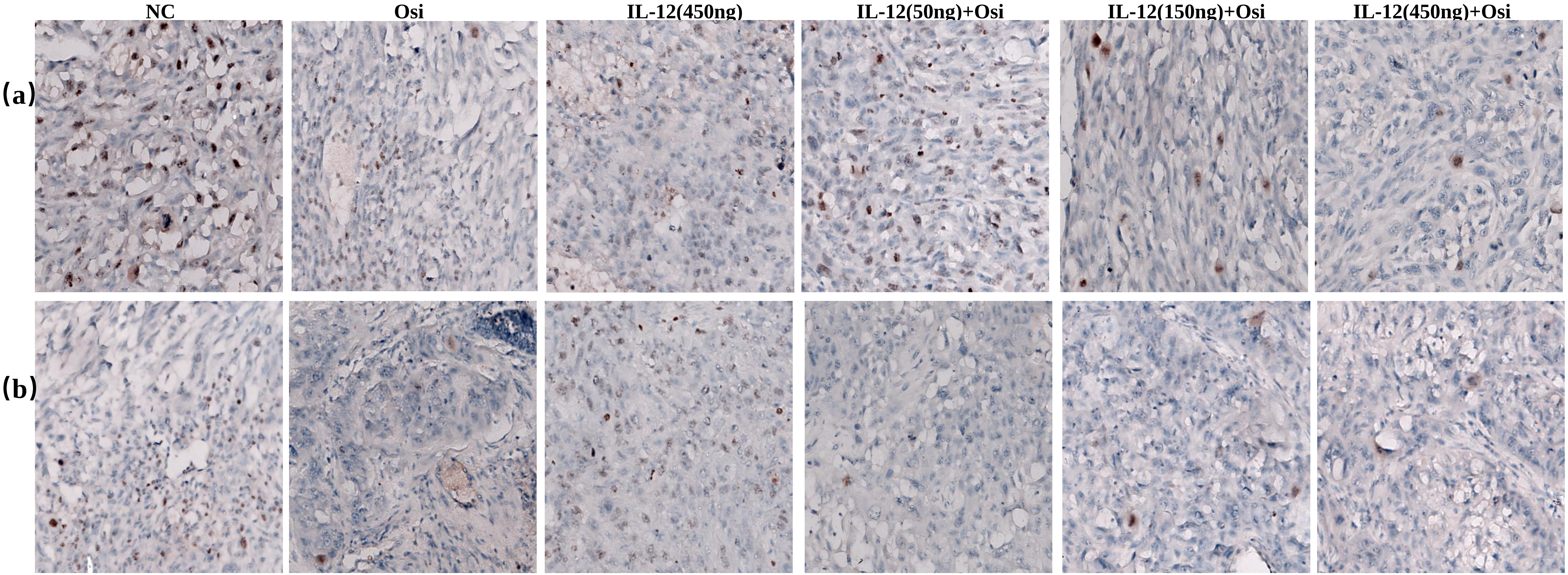
NC
Osi
IL-12(450ng)
IL-12(50ng)+Osi
IL-12(150ng)+Osi
IL-12(450ng)+Osi
(a)
(b)

Figure 10

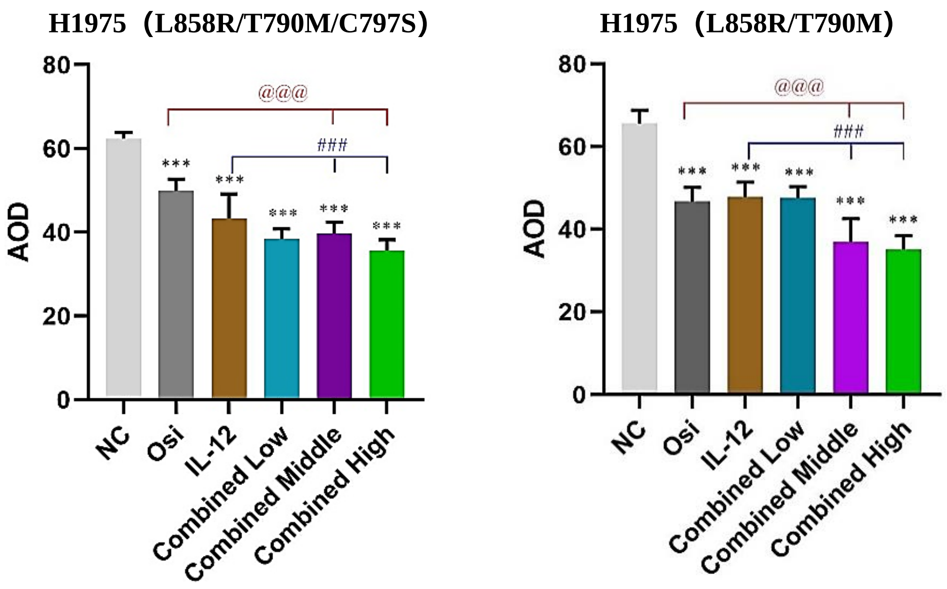

H1975（L858R/T790M/C797S）
AOD
80
60
40
20
0
@@@
###
***
NC
Osi
IL-12
Combined Low
Combined Middle
Combined High
H1975（L858R/T790M）
AOD
80
60
40
20
0
@@@
###
***
NC
Osi
IL-12
Combined Low
Combined Middle
Combined High

Supplementary Figures

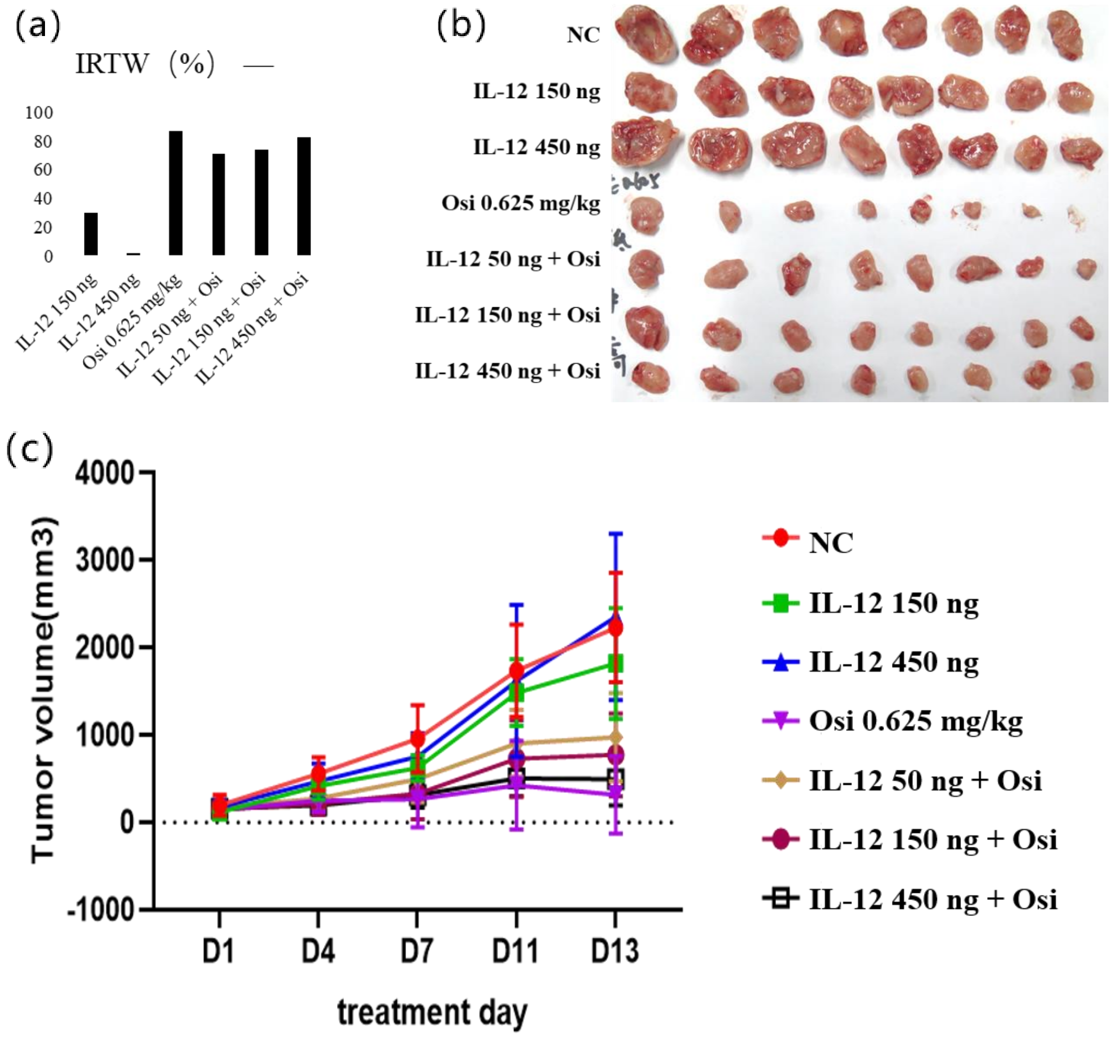

Supplementary Figure 1. Experiment with the BAF3 (EGFR L858R/T790M) subcutaneous xenograft mouse model. IL-12 was administered in two dosage groups: low and high, with dosages of 150ng per mouse and 450ng per mouse, respectively, while the dosage of Osimertinib (Osi), both as a single agent and in combination, was 0.625mg/kg. (a) The tumor inhibition rates of each group, (b) tumor dissection images at the end of the experiment, and (c) tumor volume curves for each group. Single-agent Osimertinib significantly inhibited the growth of BAF3 (EGFR L858R/T790M) subcutaneous xenografts, with a tumor inhibition rate of 86.6275% ($P<0.01$). When combined with the test substance KLT-1101, the tumor inhibition rates for the combination therapy in low, medium, and high dose groups were 70.6794%, 73.8918%, and 82.4757%, respectively. No enhanced efficacy was observed with IL-12 as a single treatment.
